# Clinical Evaluation of the BD FACSPresto^™^ Near-Patient CD4 Counter in Kenya

**DOI:** 10.1371/journal.pone.0157939

**Published:** 2016-08-02

**Authors:** Francis Angira, Benta Akoth, Paul Omolo, Valarie Opollo, Scott Bornheimer, Kevin Judge, Henok Tilahun, Beverly Lu, Imelda Omana-Zapata, Clement Zeh

**Affiliations:** 1 Kenya Medical Research Institute/US CDC Research and Public Health Collaboration, Kisumu, Kenya; 2 BD Biosciences, 2350 Qume Drive, San Jose, California, United States of America; 3 US Centers for Disease Control and Prevention (CDC-Kenya), Kisumu, Kenya; Asociacion Civil Impacta Salud y Educacion, PERU

## Abstract

**Background:**

The BD FACSPresto^™^ Near-Patient CD4 Counter was developed to expand HIV/AIDS management in resource-limited settings. It measures absolute CD4 counts (AbsCD4), percent CD4 (%CD4), and hemoglobin (Hb) from a single drop of capillary or venous blood in approximately 23 minutes, with throughput of 10 samples per hour. We assessed the performance of the BD FACSPresto system, evaluating accuracy, stability, linearity, precision, and reference intervals using capillary and venous blood at KEMRI/CDC HIV-research laboratory, Kisumu, Kenya, and precision and linearity at BD Biosciences, California, USA.

**Methods:**

For accuracy, venous samples were tested using the BD FACSCalibur^™^ instrument with BD Tritest^™^ CD3/CD4/CD45 reagent, BD Trucount^™^ tubes, and BD Multiset^™^ software for AbsCD4 and %CD4, and the Sysmex^™^ KX-21N for Hb. Stability studies evaluated duration of staining (18–120-minute incubation), and effects of venous blood storage <6–24 hours post-draw. A normal cohort was tested for reference intervals. Precision covered multiple days, operators, and instruments. Linearity required mixing two pools of samples, to obtain evenly spaced concentrations for AbsCD4, total lymphocytes, and Hb.

**Results:**

AbsCD4 and %CD4 venous/capillary (N = 189/ N = 162) accuracy results gave Deming regression slopes within 0.97–1.03 and R^2^ ≥0.96. For Hb, Deming regression results were R^2^ ≥0.94 and slope ≥0.94 for both venous and capillary samples. Stability varied within 10% 2 hours after staining and for venous blood stored less than 24 hours. Reference intervals results showed that gender—but not age—differences were statistically significant (p<0.05). Precision results had <3.5% coefficient of variation for AbsCD4, %CD4, and Hb, except for low AbsCD4 samples (<6.8%). Linearity was 42–4,897 cells/μL for AbsCD4, 182–11,704 cells/μL for total lymphocytes, and 2–24 g/dL for Hb.

**Conclusions:**

The BD FACSPresto system provides accurate, precise clinical results for capillary or venous blood samples and is suitable for near-patient CD4 testing.

**Trial Registration:**

ClinicalTrials.gov NCT02396355

## Introduction

Timely and appropriate initiation of antiretroviral treatment for HIV-positive subjects reduces morbidity and mortality associated with infections [1, 2]. Moreover, eligibility for antiretroviral therapy for HIV/AIDS and monitoring progression of the disease commonly have been based on the number of CD4+ T lymphocytes in a patient’s venous blood [3–7]. Determination of CD4+ T lymphocytes is usually done at central laboratories, requiring collection and transportation of the blood from healthcare facilities where patients are referred for HIV care and/or treatment. Depending on the laboratory capacity, the CD4 results may be available between 2–14 or more days [8] after blood sampling, delaying the start of treatment for newly diagnosed patients and increasing the risk of loss-to-follow-up [9, 10]. Introduction of point-of-care CD4+ cell counters can improve access to quick and reliable CD4+ T-cell counts in HIV-positive patients. Access to care and enabling initiation of treatment during a single clinic visit increase the efficiency and effectiveness for monitoring and staging of HIV patients [10–14].

Absolute CD4+ cell count (AbsCD4) is a robust surrogate marker for immune competence in HIV-infected adults. However, percentage of CD4+ cells in the lymphocyte population (%CD4) has been considered a reliable surrogate marker for children less than 5 years of age, since the AbsCD4 count varies more than %CD4 due to the lymphocyte development cycle [15, 16]. Anemia is a hematological abnormality that may result from the use of antiretroviral treatment (ART) with zidovudine (ZDV) [17] or as a concomitant condition [18]. Anemia has been defined as a surrogate marker for HIV/AIDS disease progression in pregnant women [19]. Determination of hemoglobin (Hb) concentration is used as surrogate marker of anemia. The availability of AbsCD4, %CD4, and Hb concentration from a single blood sample offers an integrated diagnostic result for more comprehensive management of HIV-infected patients in resource-limited settings, closer to the patient’s residence, potentially reducing loss-to-follow-up, promoting initiation of treatment, and improving treatment compliance.

The BD FACSPresto Near-Patient CD4 Counter is a new system containing unit-dose cartridges that automatically perform sample preparation from a single drop of venous or capillary blood, and a small and portable instrument that automatically analyzes the sample and reports AbsCD4, %CD4, and Hb results. The BD FACSPresto^™^ Cartridge Kit includes the following: the BD FACSPresto^™^ Cartridge, BD Microtainer^®^ Contact-Activated Lancet, sterile alcohol prep pads, plastic adhesive bandage, sterile non-woven sponge, and transfer pipettes. The BD FACSPresto Cartridge (“cartridge”) contains dried fluorochrome-conjugated antibody reagents (CD4 PE-Cy^™^5/CD3 APC/CD45RA APC/ CD14 PE) and integrated reagent QC, eliminating the need for refrigeration of reagents. The BD FACSPresto uses fluorescence microscopy and absorbance spectroscopy to interrogate the cartridge, has embedded software to analyze the results, and includes integrated instrument quality control. The duration of the whole process, from blood application onto the cartridge to results, is approximately 23 minutes.

It is hypothesized that the CD4, %CD4 and Hb results from BD FACSPresto system and cartridge are accurate, robust, reproducible and linear when using blood within 24 h of draw. The primary objective of this investigation is to demonstrate the performance of the BD FACSPresto system is similar to the performance of commercially available standard of care instruments and reagents for enumeration of CD4 and Hb using matched venous and capillary blood specimens from both HIV-positive and HIV-negative subjects. The secondary objective shows that the BD FACSPresto CD4/ %CD4 and Hb results from venous and capillary blood are comparable.

Evaluation of the BD FACSPresto system comprised five sub-studies: accuracy, stability, precision, linearity, and reference intervals, at two sites, the HIV laboratory of the collaboration between the Kenya Medical Research Institute (KEMRI), and the US Centers for Disease Control (CDC) in Kisumu, Kenya (KEMRI/CDC), and at BD Biosciences, San Jose, California, USA. The clinical evaluation of the BD FACSPresto system required specimens prospectively collected from representative cohorts under care of a healthcare institution in Kisumu, Kenya, where there is a high prevalence of HIV infections that are diagnosed and monitored using the BD FACSCalibur^™^ system, the standard of care or predicate system for CD4. BD Biosciences completed the FACSPresto linearity and precision analytical studies.

## Materials and Methods

At KEMRI/CDC, ethical approval was obtained from the KEMRI/CDC Ethics Review Committee for evaluation of accuracy and stability protocols in May 12, 2013; amended twice (September 26, 2013 and April 17, 2014) to increase the cohort and include HIV-negative subjects for the reference intervals protocol. Study subject enrollment started in December 2013 and was completed by August 2014. The study design, sample size and data analysis procedures described were derived from the Clinical Laboratory Standards Institute (CLSI) EP09-A2-IR accuracy and EP24-A2 bias estimation guidelines [20, 21]. The stability study tested the effects of blood storage and blood staining time on the observed results. Evidence of assay precision (20-run repeatability, within-run, and total precision), linearity, and normal reference intervals for AbsCD4, %CD4, and Hb were established in accordance with CLSI guidelines EP05-A2 for precision [22], EP06-A for linearity [23], and EP28-A3c for reference intervals [24]. The determination of the patient cohort sample size was based on exceeding by 25% the minimum enrollment recommended in the CLSI guidelines [21, 24] within the assay range.

Specimens used for the linearity study, executed at BD Biosciences, were procured under the BD In-House Blood Donor program with the oversight of the Institutional Review Board. All protocol procedures were conducted under Good Clinical Practices [25] and Good Laboratory Practices guidelines to ensure safety and privacy of subjects participating in the study and compliance with approved protocols.

The testing for accuracy, stability, and reference intervals was carried out at KEMRI/CDC. Subjects attending to the HIV clinic for CD4 testing were approached, if they were interested and met the inclusion criteria and none of the exclusion criteria (S1 Table) per protocol.

All subjects provided written informed consent prior to their participation in the study. Minors (<18 years of age) were distinguished by legal definition between “mature” and “non-mature.” Mature minors were married, parents, or heads of household, and could consent to participate in the study as they would do for HIV counseling and testing in Kenya [26], the KEMRI Ethics Committee approved the method of informed consent used for mature minors. Non-mature minors required written informed consent from the parents/guardians, followed by a private discussion with the minors to explain the study, followed by the minors’ written consent.

For subjects enrolled in studies at KEMRI/CDC, both venous and capillary blood specimens were collected from the same subject by trained phlebotomists. Capillary blood collection was done in accordance with the CLSI H04-A6 document [27], using a BD Microtainer contact-activated blue lancet (1.5-mm width x 2.0-mm depth; BD, Franklin Lakes, NJ). One large drop of capillary blood was transferred directly onto the cartridge to fill the cartridge inlet (~25–30 μL). Venous blood was collected in tubes with EDTA anticoagulant (BD Vacutainer^®^ K2, BD, Franklin Lakes, NJ) and sufficient blood was added to fill the cartridge inlet (~25μL-30 μL) using the provided transfer pipette. For both capillary and venous specimens, cartridges were prepared in duplicate and per the manufacturer’s instructions. After blood draw, subjects were observed to ensure the bleeding through the site of skin puncture during venipuncture or finger-stick procedures had ceased; the subjects were dismissed concluding their participation in the study. There were no follow-up visits scheduled and no results from the study were reported to clinicians. T3he phlebotomists at the sites were blinded to the testing laboratory procedures, and the laboratory technologists were blinded to the specimen collection and enrollment procedures.

Precision and linearity testing was conducted at BD Biosciences. For linearity, the venous blood specimens were procured from the In-House Donor, the donor involvement was limited to the blood draw procedures. For precision, duplicate control samples were used, and for linearity, triplicate manipulated venous samples were used, as described in more detail as follows. In each case the samples were transferred directly onto the cartridge using the provided transfer pipette per the manufacturer’s instructions.

**Fig 1:**
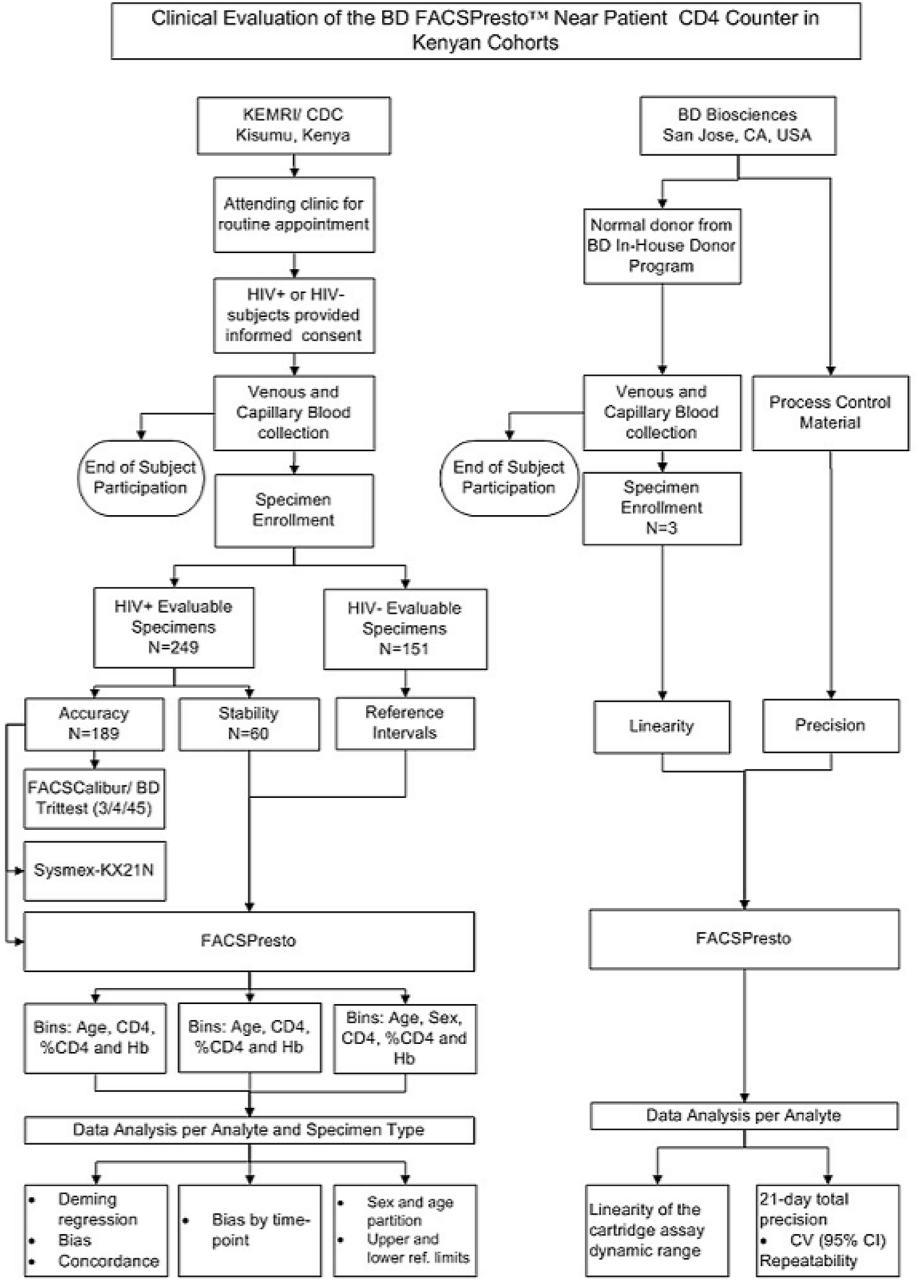
Clinical Evaluation of the BD FACSPresto^™^ Near Patient CD4 Counter Flowchart. The clinical evaluation of the BD FACSPresto was carried out at KEMRI/CDC and BD Biosciences comprised of the five sub-studies depicted in the flowchart. KEMRI/CDC evaluated the FACSPresto system using venous and capillary blood specimens from subjects attending to the clinic for routine visit. Two analytical sub- studies using normal blood and process control materials were completed at BD Biosciences.

The KEMRI/CDC Ethics Committee and BD Biosciences considered the procedures for venous or capillary blood draw involved minimal risk to the subjects during the evaluation of an *in-vitro* device; in addition, the results from the study were delinked from the patient information and not reported to the clinicians due to the investigational nature of the BD FACSPresto system, consequently no medical or clinical decisions were derived from the clinical study results. KEMRI/CDC or BD Biosciences had no requirement for mandatory registration of the minimal risk clinical studies prior to the subject or specimen enrollment. The authors confirm that all ongoing Accuracy or Method Comparison testing for this system is registered in ClinicalTrials.gov. Fig 1 illustrates the studies conducted at KEMRI/CDC and Biosciences.

### Accuracy

The accuracy evaluation assessed the equivalency of results between the BD FACSPresto system and results obtained using a standard-of-care predicate method for CD4 enumeration (BD FACSCalibur flow cytometer, BD Tritest CD3/CD4/CD45 reagent with BD Trucount tubes, and BD Multiset software). The reference system for measurement of Hb concentration was the Sysmex^™^ Automated Hematology Analyzer KX-21N (referred as Sysmex KX-21N). Instruments used in the different studies included nine BD FACSPresto systems, two BD FACSCalibur systems, and two Sysmex KX-21N hematology analyzers, as well as six different lots of cartridges.

Venous and capillary blood samples were tested on the BD FACSPresto system, and venous blood was further tested in duplicate using two reference or predicate methods. The first reference method was for determination of AbsCD4 and %CD4: BD Tritest CD4/CD45/CD3 reagent in BD Trucount tubes with data acquired on a BD FACSCalibur flow cytometer and analyzed using BD Multiset software (BD Biosciences, San Jose, CA), operated according to the manufacturer’s instructions. The second reference method was for determination of Hb concentration in venous blood using a Sysmex KX-21N system (Sysmex America, Mundelein, IL), operated according to the manufacturer’s instructions. Venous blood was tested within 6 hours of collection on BD FACSCalibur and BD FACSPresto systems, and on a Sysmex KX-21N system. Capillary blood was always transferred immediately to the cartridge, and both capillary and venous blood samples were incubated in the cartridges between 18 minutes and 2 hours.

On each day of testing, the BD FACSPresto instrument was turned on, the instrument QC test was automatically performed, and the results printed. CD4 and Hb external quality controls were run on the corresponding instruments before testing patient samples. BD^™^ Multi-check low and normal (BD Biosciences, San Jose, CA) quality controls for CD4 were used for the BD FACSCalibur system. CD Chex^™^ Plus BC low and normal (Streck, Omaha, NE) quality controls for CD4 were used for the BD FACSPresto system. Three levels of Hb controls (low, normal, and high levels) were used in the Sysmex (EIGHTCHECK-3WP-X-TRA Sysmex, Kobe, Japan). For BD FACSPresto, three levels of Eurotrol 301 (level 1, 2, and 3; Eurotrol B.V., Ede, The Netherlands) Hb control were also used. The BD FACSPresto system was used in several other sub-studies (described as follows), and the control procedures described were followed on a daily basis. In addition, specimens were enrolled by bins to ensure statistical representation across the assay ranges for each parameter (S1 File). The bins and ranges for CD4, %CD4, and Hb are shown in Table 1.

**Table 1:** Accuracy Results by Specimen Type and Bins.

Parameter	Specimen Type	Bins*	N	Min	Max	Mean	SD	Mean %sim	%sim SD
**AbsCD4 (cells/μL)**	**Venous**	50–250	29	55	247	149.1	59.7	102.5	8.6
		250–500	59	255	498	380.9	72	99.2	4.7
		500–1,000	76	503	997	685.6	147.4	99.3	3.3
		1,000–4,000	25	1,020	2,478	1,423.8	393.6	100.1	2.4
		Sub-total	189						
	**Capillary**	50–250	12	69	242	154.5	60.8	108.7	10.3
		250–500	54	262	494	382.1	65.3	102.5	6.7
		500–1,000	68	510	998	703.7	137.4	103.8	5.7
		1,000–4,000	28	1,003	2,474	1,397.5	459.5	102.7	6.4
		Sub-total	162						
**%CD4 (%)**	**Venous**	5–20	53	5.1	19.9	14.7	3.7	100.8	6
		20–35	98	20.2	35	27.9	4.4	102.4	3
		35–60	36	35.5	53.8	41.5	4.1	101.4	2
		Sub-total	187						
	**Capillary**	5–20	48	5.9	19.8	14.9	3.6	99.5	4.6
		20–35	88	20.2	34.9	27.4	4.5	100.7	3.8
		35–60	24	35.1	56.2	40.4	5.1	100.9	2.5
		Sub-total	160						
**Hb (g/dL)**	**Venous**	2–9	34	3	8.9	6.7	2.2	100.4	8
		9–12	78	9	11.9	10.9	0.7	97.8	1.6
		12–17	66	12	16.6	13.7	1.2	98.1	1.5
		17–20	6	17.3	18.7	18.1	0.5	99.1	1.5
		Sub-total	184						
	**Capillary**	2–9	18	4.7	8.9	7.8	1.4	100.8	3.8
		9–12	70	9	11.9	10.8	0.8	98.9	2.1
		12–17	73	12	16.9	13.7	1.3	99.5	2.5
		17–20	1	17.1	17.1	17.1	NA	99.7	NA
		Sub-total	162						

*Bins based on BD FACSPresto system results. The valid results from first replicate were used to generate the summary statistics.

### Stability

The stability sub-study was designed to evaluate the effects of venous blood storage up to 24 hours after draw using the cartridges or age of blood, and the effects of the time of the stain or age of stain on both venous and capillary blood at 18 minutes and 2 hours after loading the blood onto the cartridge. This study was performed exclusively on the BD FACSPresto system. Venous blood was tested at four different time points (V0, V1, V2, and V3) and capillary blood at two time points (C0 and C1). Time zero, V0 and C0, were the reference conditions for “fresh” venous or capillary sample post-draw (testing was performed within 6 hours for venous or within 2 hours for capillary) and within 30 minutes incubation time in the cartridge for sample staining, from addition of the blood onto the cartridge to instrument interrogation. Time points V1 and C1 extended the incubation time to 120 minutes (±15 minutes). Two more time points for venous blood were studied, time point V2 had between 18 and 30 minutes incubation time with venous blood 22–24 hours after collection and, time point V3 had 120 minutes (±15 minutes) incubation time with blood 22–24 hours after collection (S1 File).

### Precision

The BD FACSPresto precision evaluation consisted of two studies. The first was a 20-run repeatability study, and the second consisted of a 21-day evaluation of within-run and total precision. For the 20-run repeatability study, one operator used one BD FACSPresto instrument and one lot of cartridges to run 20 replicates. In the 21-day precision study, one lot of process control materials for CD4 (two levels) and one lot of Hb controls (three levels) were tested by three operators, in three instruments, using three different lots of cartridges, two runs per day, with a total of 42 runs for the duration of the study.

All precision testing was carried out with commercially available control materials: CD Chex Plus BC, low and normal levels, and three levels of Eurotrol 301 Hb control. These materials were used as controls and samples for both studies, the 20-run repeatability and 21-day precision (S2 File).

### Linearity

For linearity testing, normal whole blood specimens were obtained by separating and recombining cell and plasma blood fractions to prepare two sample pools (high and low). Low and high pools were mixed to obtain 11 equally spaced concentrations over a range 20%–30% wider than the anticipated measuring range. While the instrument reports %CD4, estimation of %CD4 linearity was not included in this analysis. %CD4 is an indirect measurement derived from the total lymphocyte (Lymphs) and the AbsCD4 cell populations. Linearity for Lymphs was evaluated (a special software mode was used, since lymph results are not available in the ordinary BD FACSPresto). Linearity was calculated for AbsCD4, Lymphs, and Hb using one lot of the cartridges. Each concentration was run in triplicate. Measured results were compared to the anticipated results per dilution to establish the assay linearity (S3 File).

### Reference Intervals

Specimens from enrolled HIV-negative healthy subjects, free of hematological abnormalities were grouped by age and gender addressing the reference intervals for CD4, %CD4 and Hb three analytes of the BD FACSPresto cartridge. Venous and capillary blood samples were run in duplicate within 18–120 minutes incubation time to determine the reference intervals (S1 File).

### Statistical Analysis

Study design and analysis methods are in accordance with CLSI guidelines EP09-A2-IR[21] and EP24-A2[20] for accuracy, EP05-A2 [22] for precision, EP06-A [23] for linearity, and C28-A3c [24] for reference intervals. Data collected on Case Report Forms and instrument files was integrated in the study-specific database. Only evaluable samples were included in the analysis using statistical software packages (SAS^®^ v9.3, SAS Institute, Cary, NC; Analyse-it^®^ v2.22, Analyse-it Software Ltd, Leeds, UK; Microsoft^®^ Excel^®^ v12.0, Microsoft, US, and CBstat5 v5.1.0, US). For accuracy, stability, and reference intervals, the first replicate with valid results was used for statistical analysis; the second replicate was used as an outlier check to ensure the quality of the data. Outliers were identified and investigated[21], and no outliers were removed from analysis.

#### Accuracy

The venous and capillary results from the BD FACSPresto system were analyzed independently against the venous results from BD FACSCalibur or Sysmex predicate systems (S4 File). Agreement of BD FACSPresto venous and capillary and predicate results was assessed by Deming regression [28] and by Bland-Altman analysis [29] (absolute bias, relative bias, and limits of agreement). Weighted Deming regression was used to stabilize the variance in the regression and obtain an accurate estimate of the intercept. The weight adopted in this analysis was the inversed average squared. [30]. Weighted Deming regression [28, 30] was only performed on AbsCD4, since the variance increases as the counts increase over a large range. Ordinary Deming or un-weighted Deming regression was performed on CD4% and Hb since the values cover a much smaller range and thus can be expected to have relatively similar variance.[31–33]. Predicted bias intervals from Deming regression were also reported around recommended cutoffs for AbsCD4 counts[30] of 200, 350, 500, and 750 cells/μL; for %CD4[31] at 15%, 20%, 25%; and Hb[30] at 4, 5, 10.5, and 17 g/dL. In addition, concordance analysis was performed around these cutoffs, and exact confidence intervals (CIs) were reported using the Wilson score method [34, 35]. Finally, venous blood and capillary blood Deming regression analyses for AbsCD4, %CD4, and Hb between were included. Analysis included estimation of the percentage similarity value (%sim) and standard deviation [21, 36]. A small number of samples were excluded from analysis due to instrument errors described below.

#### Stability

The stability study tested the relative bias between samples run at”time zero” (fresh capillary blood or venous blood <6 hours post-draw and <30 minutes incubation time in the cartridge) vs later time points of incubation or (venous) blood age. Venous and capillary blood time points per specimen were analyzed independently. The percent bias between each study time point and baseline time point (V0 or C0) was calculated for each specimen. Then the overall percent bias was calculated at each study time point, C1, V1, V2, and V3, across all the specimens and the data was pooled, yielding the mean percent bias, SD, and the lower and upper confidence intervals for each time point tested. The 95% CI around the mean bias was calculated based on the T-distribution (S5 File).

#### Precision

For the 20-run repeatability study, the standard deviation (SD) and coefficient of variation (CV) were reported. In the 21-day precision study, within-run and total precision were calculated for AbsCD4, %CD4, and Hb. Within-run precision means the same operator runs the same instrument using the same lot of cartridges within a short time period. Variance for AbsCD4, %CD4, and hemoglobin were estimated by ANOVA, reporting the SD(s) and percent of coefficient of variation (%CV) with the upper 95% CI for within-run and total precision. Data from 42 runs (2 runs per day during 21 days) with duplicate values were included in the analysis. Precision for AbsCD4, %CD4, and Hb concentration was analyzed separately.

#### Linearity

Weighted polynomial regression models (1^st^ order, 2^nd^ order, and 3^rd^ order) were fitted to the measured results as a function of the expected values or coded levels for each of the variables: a) AbsCD4, b) total Lymphs, and c) Hb. All results from triplicate cartridges were used in the regression analysis. The coefficients for the nonlinear terms (2^nd^ order and 3^rd^ order terms) of the higher order models were tested to determine statistical significance. If the higher order coefficient differences were not statistically significant, then the data was deemed linear with no further analysis required. If the higher order coefficients were statistically significant, then the degree of non-linearity was studied by taking the difference between the best higher order fit and the 1^st^ order linear fit at each concentration level. If the difference was within the acceptance criteria for each level, then the data was adequately linear[23]. The pooled data across the different concentration levels was reported to ensure good fit (S6 File).

### Reference Intervals

Reference intervals were analyzed in venous (N = 151) and capillary (N = 150) blood specimens using only the first replicate measurement and calculating the 2.5 and 97.5 percentiles of the observed measurement range for AbsCD4, %CD4, and Hb per specimen type, gender and age. Differences by age and gender were tested for statistical significance (S7 File).

## Results

### Demographic Information

The total number of HIV+ enrolled subjects included 241 (179 in accuracy and 62 in stability studies) from KEMRI/CDC. An additional 21 HIV+ venous blood specimens were procured from an external vendor for sample manipulation to fill the accuracy bins at BD; and HIV-negative subjects free of hematological abnormalities were also enrolled (N = 155), 152 in the reference interval study at KEMRI/CDC, and 3 in the linearity studies (Fig 1).

The median age of the adolescent/adult population was 36 years (range, 18–77 years), and for children the median age was 8 years (range 2–17 years) in the accuracy and stability studies; by gender, 64% were females (N = 154), and 36% were male subjects (n = 87). During enrollment, patient verbally reported co-morbid conditions, including anemia (2%); malaria (4.5%), tuberculosis (2%), diabetes mellitus (1.6%), and high blood pressure (1.7%). Enrolled subjects reported taking medications that included antiretroviral drugs (79%), and Septrin or Dapson as prophylaxis (95%). The right hand was more frequently used (62%) for capillary blood collection.

### Accuracy

Specimens tested according to the protocol requirements that provided valid results were included in analysis and grouped in predefined bins for AbsCD4, %CD4, and Hb parameters based on the analytical range of the assay. The number of specimens with valid results included in the analysis varied by specimen type venous/capillary (N = 189/N = 162) and parameter: AbsCD4 (187/160), %CD4 (184/162), and Hb (190/163). All capillary blood specimens had a corresponding venous sample from the same subject. The descriptive statistics for AbsCD4 count, %CD4, and Hb measured values are illustrated in Table 1. This table also includes the mean percent similarity (%sim) and %sim standard deviation (SD) results per bin. Eleven specimens were completely excluded form analysis; the reasons for exclusion were the following: the process controls were not acquired prior to acquiring patient data, results were outside the validated claim range or suppressed by the instrument or one replicate was missing.

**Fig 2:**
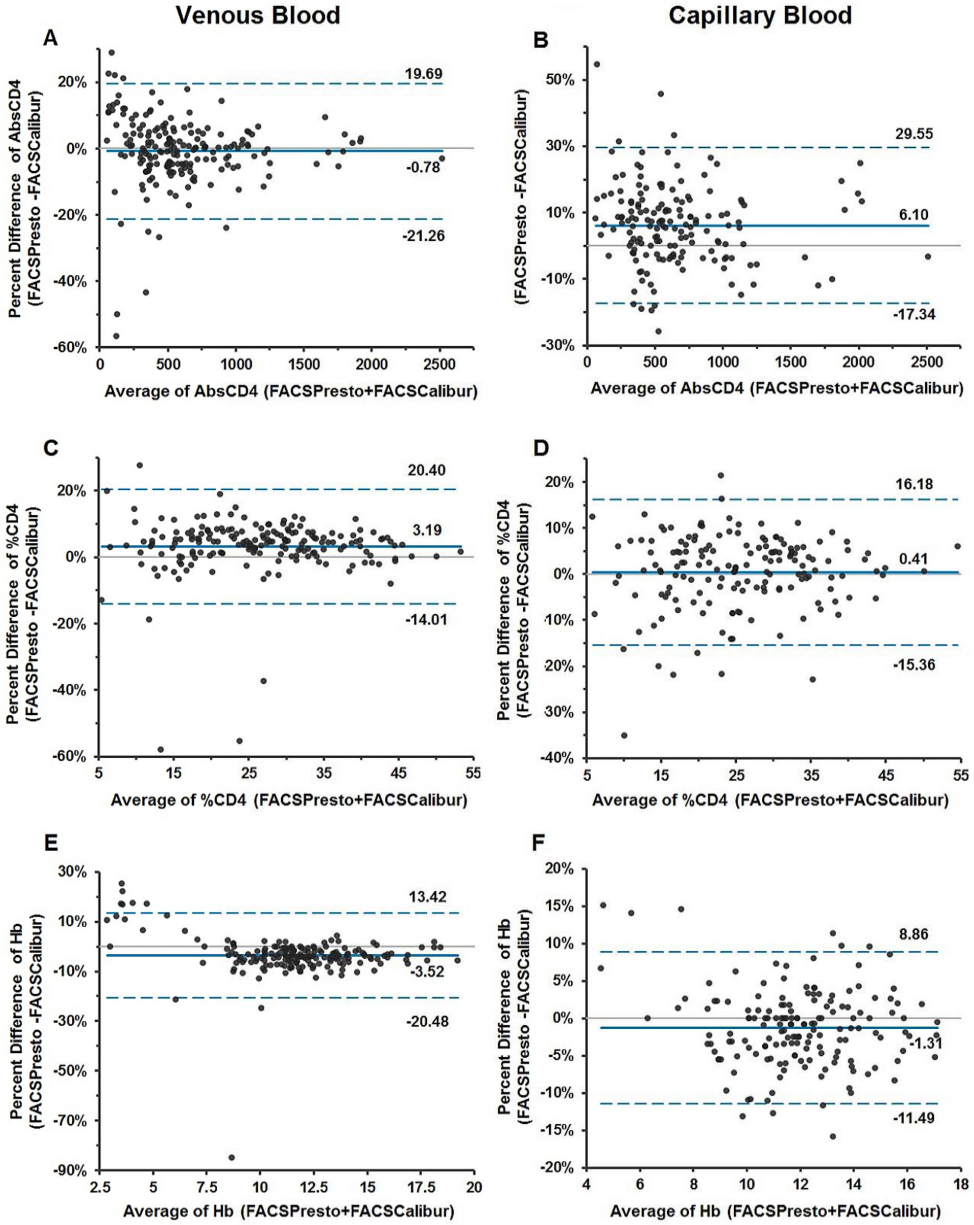
Bland-Altman Plots for AbsCD4, %CD4 and Hb in Venous and Capillary Blood. Bias results are illustrated in the Bland-Altman plots with limits of agreement for venous blood (2A, 2C and 2E) on the left side and for capillary blood (2B, 2D and 2F) on the right side. Biases for AbsCD4 are show in 2A and 2B; for %CD4 in 2C and 2D and hemoglobin in 2E and 2F. The x-axis displays the average (AbsCD4, %CD4 or Hb) and the y-axis is the difference (AbsCD4, %CD4 or Hb).

The Bland-Altman bias results for venous AbsCD4 and %CD4 showed a mean percent bias with 95% limits of agreement (LoA) for AbsCD4 of -0.78 (-21.26; 19.69) cells/μL and %CD4 of 3.19 (-14.01; 20.40); for capillary blood was 6.1 (-17.34; 29.55) cells/μL AbsCD4 and for %CD4, the mean bias was 0.41 (-15.36;16.18). For Hb, the Bland-Altman Hb bias analysis with 95% LoA for venous blood was -3.53 (-20.48; 13.42) g/dL, and for capillary blood was -1.31 (-11.49; 8.86) g/dL) as illustrated in the Bland-Altman plots Fig 2.

Table 2 summarizes the results from three analyses, mean bias by Deming Regression and Bland-Altman and mean %sim with the lower and upper limits. Notice the Bland-Altman mean %bias is reported with 95% limits of agreement (LoA), whereas the mean %bias and the mean %sim are reported with 95% confidence intervals (95% CI). The valid results from first replicate were used to generate the summary statistics.

**Table 2:** Deming Regression, Bland-Altman Mean Bias and Mean Percent Similarity Results Comparison with Limits of Agreement.

Specimen	Parameter	N	Mean %Bias (95%) CI of Mean Bias)	Bland-Altman Mean %Bias (95% LoA)	Mean %Similarity (95% CI of the Mean %sim)
**Venous**	**AbsCD4 (cells/μL)**	189	-0.28(-1.46;0.91)	-0.78(-21.26;19.69)	99.86(99.27;100.45)
	**%CD4 (%)**	187	3.6(2.63;4.56)	3.19(-14.01;20.40)	101.80(101.32;102.28)
	**Hb (g/dL)**	184	-3.16(-4.08;-2.23)	-3.53(-20.48;13.42)	98.42(97.96;98.88)
**Capillary**	**AbsCD4 (cells/μL)**	162	7.11(5.37;8.86)	6.1(-17.34; 29.55)	103.56(102.68;104.43)
	**%CD4 (%)**	160	0.72(-0.3;1.74)	0.41(-15.36;16.18)	100.36(99.85;100.87)
	**Hb (g/dL)**	162	-1.17(-1.85;-0.5)	-1.31(-11.49;8.86)	99.41(99.08;99.85)

In addition, the linear Deming regression results for each parameter in venous or capillary blood are depicted as R^2^, slope and intercept in Table 3. The Deming regression graphs (S1 Fig) can be found in Supplement section.

**Table 3:** Summary of the Deming Regression Results.

Specimen	*Parameter*	R^2^	Slope (CI)		Intercept (CI)	
**Venous**	**CD4 (cells/μL)**	0.98	0.97	(0.95,0.99)	7.37	(-0.03,14.76)
	**%CD4 (%)**	0.96	1.03	(1.00,1.05)	0.13	(-0.55,0.85)
	**Hb (g/dL)**	0.96	0.94	(0.91,0.98)	0.18	(-0.27,0.64)
**Capillary**	**CD4 (cells/μL)**	0.97	1.03	(0.99,1.07)	13.47	(-1.91,28.85)
	**%CD4 (%)**	0.96	1.02	(0.99,1.05)	-0.26	(-1.03,0.49)
	**Hb (g/dL)**	0.94	0.98	(0.94,1.02)	0.08	(-0.41,0.55)

The predicted bias from Deming regression with 95% CI at candidate decision points of 200, 350, 500 and 700 cells/μL, as well as for 15, 20 and 25% for %CD4 are reported (Table 4).

**Table 4:** Deming Regression Predicted Accuracy Interval at Different Cutoffs.

Type	Parameter	Decision Level	Bias (with 95% CI)	Percent (%) Bias (with 95% CI)
**Venous**	**CD4 (cells/μL)**	200	1.7 (-3.8, 7.2)	0.85 (-1.9, 3.6)
		350	-2.5 (-7.7, 2.7)	-0.71 (-2.2, 0.77)
		500	-6.8 (-12.8, -0.7)	-1.36 (-2.56, -0.14)
		750	-13.8(-22.9, -4.8)	-1.84(-3.05, -0.64)
	**%CD4 (%)**	15%	0.55 (0.16, 0.94)	3.67 (1.07, 6.27)
		20%	0.69 (0.37, 1.01)	3.45 (1.85, 5.05)
		25%	0.83 (0.55, 1.11)	3.32 (2.20, 4.44)
	**Hb (g/dL)**	4.5	-0.07 (-0.36, 0.22)	-1.56 (-8.00, 4.89)
		10.5	-0.40 (-0.52, -0.27)	-3.81 (-4.95, -2.57)
		17	-0.76 (-0.89, -0.62)	-4.47 (-5.24, -3.65)
**Capillary**	**CD4 (cells/μL)**	200	19.6 (9.9, 29.4)	9.8 (4.95,14.7)
		350	24.3 (16.5, 32.0)	6.94 (4.71, 9.14)
		500	28.9 (19.5, 38.2)	5.78 (3.90, 7.64)
	**%CD4 (%)**	15%	36.6(20.2, 53.0)	4.9(2.7, 7.1)
		20%	-0.01 (-0.38, 0.36)	-0.07 (-2.53, 2.4)
		25%	0.07 (-0.22, 0.37)	0.35 (-1.10, 1.85)

In addition, the two-by-two (2x2) concordant table with the overall, positive and negative agreements for AbsCD4 in venous blood at 350 cells/μL are shown in Table 5.

**Table 5:** Venous AbsCD4 Comparison between BD FACSPresto and BD FACSCalibur Systems at Cutoff of 350 cells/μL.

**Agreement**		**BD FACSCalibur**		
		**Positive**	**Negative**	**Total**
**BD FACSPresto**	**Positive**	44	2	46
	**Negative**	6	137	143
	**Total**	50	139	189
Overall Agreement** = (44+137)/189*100% = 95.8%				
Positive Agreement*** = (44/50)* 100% = 88.0%				
Negative Agreement^&^ = (137/139)*100% = 98.6%				

**Overall agreement is defined as the proportion of subjects in whom the new test and the non-reference standard give the same outcome.

***Positive agreement refers to the proportion of non-reference standard positive subjects in whom the new test is positive or ≤cutoff.

^&^Negative agreement is defined as the proportion of non-reference standard negative subjects in whom the new test is negative or >cutoff.

The concordance analysis for additional decision points is summarized (Table 6) as overall agreement with 95% CI and positive percent and negative percent agreement.

**Table 6:** BD FACSPresto and BD FACSCalibur Agreement at Different Cutoffs.

**Parameter**	**Type**	**Agreement at Cutoffs**	**200**	**350**	**500**	**750**
**AbsCD4 (cells/μL)**	**Venous**	Overall	99.5%	95.8%	96.3%	99.5%
		95% CI^#^ (LL/ UL)	97.1/ 99.9%	91.9/ 97.8%	92.6/ 98.2%	97.1/ 99.9%
		Positive Percent Agreement	100.0%	88.0%	93.2%	100.0%
		Negative Percent Agreement	99.4%	98.6%	99.0%	98.0%
	**Capillary**	Overall	98.80%	94.40%	95.1%	95.1%
		95% CI^#^ (LL/ UL)	95.6/ 99.7%	89.8/ 97.1%	90.6/ 97.5%	90.6 97.5%
		Positive Percent Agreement	100.00%	87.10%	93.9%	100.0%
		Negative Percent Agreement	98.7%	96.2%	95.8%	84.3%
**Parameter**	**Type**	**Agreement at Cutoffs**	**25%**			
**%CD4 (%)**	**Venous**	Overall	93.1%			
		95% CI^#^ (LL/ UL)	88.5/ 95.9%			
		Positive Percent Agreement	96.3%			
		Negative Percent Agreement	90.7%			
	**Capillary**	Overall	90.1%			
		95% CI^#^ (LL/ UL)	84.5/ 93.8%			
		Positive Percent Agreement	88.8%			
		Negative Percent Agreement	91.4%			

^#^CI of the overall agreements was calculated based on the Wilson Score Method.

**Fig 3:**
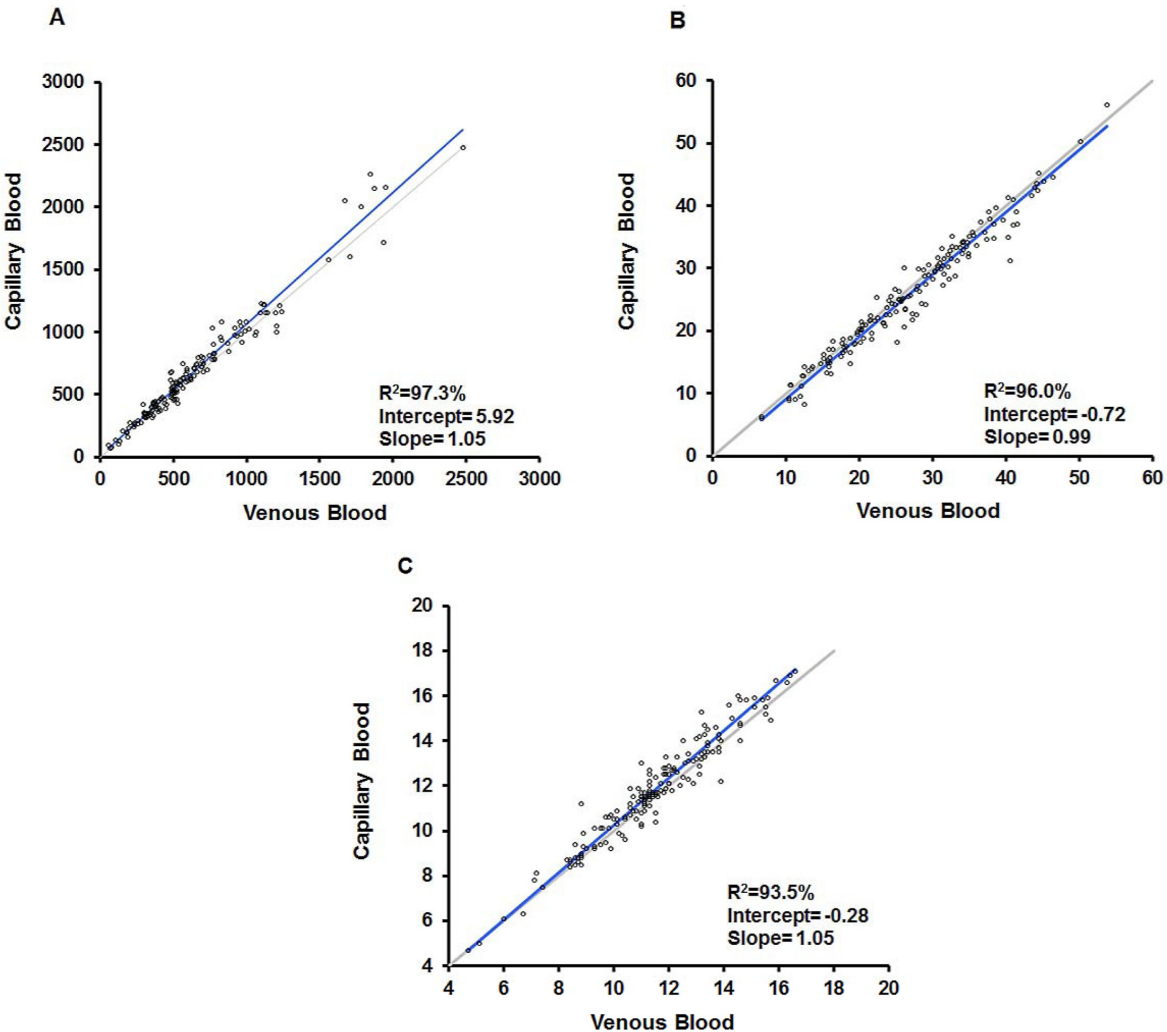
Comparison of Venous and Capillary Blood on the BD FACSPresto. Comparison of venous and capillary Deming regression results are shown for AbsCD4 (A), %CD4 (B), and Hb (C) with R^2^ (%), intercept, and slope.

The regression analysis for equivalency between venous and capillary blood results provided R^2^ ≥ 93.5% (Fig 3).

### Stability

Only specimens tested under protocol procedures that provided valid results were included in the analysis. The study included 24 manipulated venous blood specimens to assess the high end of the linear range of the assay. Eight specimens with at least one parameter with valid results were included in the statistical analysis, two specimens were completely excluded due missing the time zero results. Stability descriptive statistics results by parameter bin and specimen type are summarize in Table 7.

**Table 7:** Time Zero Stability Results Summary by Specimen Type and Bins.

Parameter	Specimen Type	Bins	N	Min	Max	Mean	SD
**AbsCD4 (cells/μL)**	**Venous**	50–250	7	54	238	158.3	67.9
		250–500	17	252	491	383.9	83.2
		500–1,000	30	510	976	704.1	149.6
		1,000–4,000	4	1,191	1,969	1,644.8	326.3
		Sub-total	58				
	**Capillary**	50–250	4	61	171	122	52.4
		250–500	12	265	462	366.3	64.1
		500–1,000	25	505	994	712.6	144.9
		1,000–4,000	6	1,049	2,461	1,433.7	540
		Sub-total	47				
**%CD4**	**Venous**	5–20	17	5.1	19.9	13.6	5.2
		20–35	29	20.7	33.5	27.6	3.8
		35–60	14	35.9	54.8	40.9	5.2
		Sub-total	60				
	**Capillary**	5–20	13	5.3	19.7	14	4.6
		20–35	22	20.3	33.9	27.8	4.1
		35–60	11	35.6	55.8	40.1	5.8
		Sub-total	46				
**Hb (g/dL)**	**Venous**	2–9	11	4.8	8.7	7.3	1.2
		9–12	14	9	11.5	10.5	0.9
		12–17	26	12	15.4	13.4	0.9
		17–20	5	17.8	19.1	18.2	0.5
		Sub-total	56				
	**Capillary**	2–9	11	4.5	8.9	7.5	1.3
		9–12	14	9.4	11.9	10.8	0.9
		12–17	23	12.1	16.3	13.9	1.1
		17–20	0	NA	NA	NA	NA
		Sub-total	47				

The AbsCD4 cells/μL mean (range) at time zero (V0/C0) was 609.24 (54–1,969) for venous blood and 666 (61–2,461) for capillary blood. The %CD4 mean (range) in venous blood was 26.7% (5.1%–54.8%) and 26.9% (5.3%–55.8%) for capillary blood. Hb concentration in g/dL showed a mean (range) of 11.9 (4.8–19.1) in venous blood and 11.5 (4.5–16.3) in capillary blood.

The mean percent biases across all stability conditions were within 6.2% for CD4, 9.3% for %CD4, and 2.1% for Hb (Table 8). Results are presented per specimen type (V or C) and time point (1, 2 or 3). Each time point is comprised by the age of the blood (0h or 22h), and the duration of the incubation of the cartridge (0h or 2h).

**Table 8:** Venous and Capillary Blood Stability Results.

Specimen Type	Parameter	Time point (h/h)		N	Mean %Bias^	SD	95% Low CI	95% Upper CI
**Venous**	**AbsCD4 (cells/ μL)**	V1	(0/2)	57	3.40	7.82	1.67	5.13
		V2	(22/0)	53	1.36	14.30	-1.93	4.65
		V3	(22/2)	54	6.17	12.24	3.38	8.96
	**%CD4**	V1	(0/2)	59	3.93	11.16	1.50	6.35
		V2	(22/0)	54	4.32	11.78	1.64	7.00
		V3	(22/2)	55	9.21	12.44	6.41	12.02
	**Hb (g/dL)**	V1	(0/2)	55	2.06	3.75	1.21	2.90
		V2	(22/0)	52	0.33	3.96	-0.59	1.25
		V3	(22/2)	54	1.88	4.66	0.82	2.94
**Capillary**	**AbsCD4 (cells/ μL)**	C1	(0/2)	46	1.49	10.38	-1.08	4.06
	**%CD4**	C1	(0/2)	44	5.55	7.82	3.56	7.53
	**Hb (g/dL)**	C1	(0/2)	47	1.52	5.44	0.19	2.86

^^^Mean %Bias is calculated with respect to a time point zero reference (V0 or C0).

### Precision

The 20-run repeatability results (Table 9), for low CD4 samples the %CV was within 5.8%, and for normal CD4 samples was within 2.6%, for CD4, %CD4, and Hb (data will be provided upon request).

**Table 9:** Precision Results—Twenty-run Repeatability.

Parameter	Sample	Mean	N	SD	SD—95% Upper CI	%CV	CV- 95% Upper CI
**AbsCD4 (cells/ μL)**	**Low**	155.1	20	8.97	12.29	5.78	7.37
	**Normal**	926.6	20	24.04	32.95	2.59	3.3
**%CD4 (%)**	**Low**	12.77	20	0.73	1.01	5.74	7.32
	**Normal**	43.99	20	0.67	0.92	1.53	1.95
**Hb (g/dL)**	**Low**	6.95	20	0.16	0.22	2.26	2.88
	**Normal**	12.99	20	0.14	0.19	1.09	1.38

The results of the 21-day precision study (Table 10a and 10b) showed the %CV for AbsCD4 and %CD4 within 6.8% for low samples and 3.4% for normal samples; and the %CV for the three levels of Hb was within 2.4% (Table 10c).

**Table 10:** Precision Results—Within-run and Total Precision.

Parameter	Sample	Precision	Mean	DF	SD	SD—95% Upper CI	%CV	%CV—95% Upper CI
**AbsCD4 **	Low	Within Run	155.96	42	10.6	12.95	6.79	8.03
		Total	155.96	83	10.6	12.16	6.79	7.67
	Normal	Within Run	940.93	42	20.55	25.1	2.18	2.58
		Total	940.93	83	31.02	35.61	3.3	3.72
**%CD4**	Low	Within Run	12.55	42	0.75	0.92	5.98	7.07
		Total	12.55	83	0.75	0.86	5.98	6.75
	Normal	Within Run	43.9	42	0.69	0.85	1.58	1.87
		Total	43.9	83	0.76	0.88	1.74	1.96
**Hb (g/dL)**	Level 1	Within Run	6.9	42	0.17	0.2	2.42	2.86
		Total	6.9	83	0.17	0.19	2.42	2.73
	Level 2	Within Run	12.74	42	0.19	0.23	1.46	1.72
		Total	12.74	83	0.19	0.22	1.52	1.72
	Level 3	Within Run	16.87	42	0.18	0.22	1.07	1.27
		Total	16.87	83	0.19	0.22	1.14	1.28

DF = degrees of freedom, refers to the number of independent values. In the precision study, the total tubes collected were 21 (days) x 2 (runs) x 2 (replicates). Therefore, the total degree of freedom is 84–1 = 83. Within-run degrees of freedom are 21 (days) x 2 (runs) = 42.

SD = standard deviation.

%CV = percent coefficient of variation.

### Linearity

The analytical range of the assay was determined by the linear relationship between the expected and the measured ranges for each parameter. The determined linear range for AbsCD4 was 42–4,897 cells/μL, for total Lymphs, 182–11,704 cells/μL, and for Hb, 2–24 g/dL, with R^2^ = 0.99 for all. The %CVs the different concentration levels were pooled using the root mean square method and all the %CVs values were ≤4.6%. Results are summarized in Table 11.

**Table 11:** Linearity Range.

Parameter	Overall %CV^!^	R^2^	Final Range	Product Claim Range
**AbsCD4 (cells/μL)**	4.36	0.997	42–4,897	50–4,000
**Total Lymphs (cells/μL)**	2.55	0.999	182–11,704	20–10,000
**Hb (g/dL)**	4.60	0.999	2–24	2–20

^!^Overall %CV = the mean of the %CV across all the triplicate measurements at each linearity interval.

### Reference Intervals

The median age for normal subjects was 29 years (range, 13–65 years). Age difference results were not statistically significant when comparing groups of 13 to <18, 18 to <30, 30 to <42, 42 to <54, and 54 to ≤65 years; however gender differences were statistically significant (p <0.01). Reference intervals per parameter, specimen type and gender are summarized in Table 12. One female subject was excluded due to age outside the range and results from second capillary specimen were suppressed (CD4 and %CD4), including only Hb results in the analysis.

**Table 12:** Reference Intervals Partitioned by Gender.

Blood Type	Subset	Gender	N	Mean	Reference Interval
	**CD4**	Male	76	881	469–1,437
	**(cells/μL)**	Female	75	1,054	503–1,679
**Venous**	**%CD4**	Male	76	39.99	29.15–52.54
	**(%)**	Female	75	42.95	30.66–54.67
	**Hb**	Male	76	14.17	10.9–16.8
	**(g/dL)**	Female	75	12.2	8.2–15
	**CD4**	Male	76	930	510–1,552
	**(cells/μL)**	Female	74	1,122	508–1,851
**Capillary**	**%CD4**	Male	76	37.92	26.86–49.39
	**(%)**	Female	74	41.37	30.56–53.16
	**Hb**	Male	76	14.56	11.2–17.5
	**(g/dL)**	Female	75	12.48	8.5–14.7

### Cartridge Failure and Error Rate

During acquisition, if there was an error with an instrument error code, the site staff typically re-ran the cartridges. A cartridge failure was considered as an unsuccessful attempt to generate results after a second acquisition of the same cartridge. This required re-preparing the sample using a new cartridge. There were 2,875 cartridge runs during the accuracy, stability, and reference interval testing, with a total of 55 cartridge failures. Fifty-four were sample replicates, and one error was for Hb control.

The overall error rate was 1.9% for accuracy, stability, and reference interval testing, the cartridge or instrument errors suppressed results from one parameter but reported results from other parameter, for example Hb. The error codes observed during testing were: 6B0F (n = 34), 6B0D (n = 19), 6B0E (n = 1), and 6B07 (n = 1). The error code 6B0F occurred most often in samples with blood stored ≥22 hours post draw and can be related to diminished cell signal. The error code 6B0D is associated with non-homogenous distribution of the blood sample in the cartridge channel, which could have been related to inappropriate blood transfer or incubation. The error 6B0E is related to unexpected cell distribution, and error 6B07 refers to insufficient quality images for the Hb analysis.

### Protocol Deviations

There were a total of four minor protocol deviations, for accuracy the FACSCalibur settings for the minimum lymphocyte events did not follow the protocol, in addition, two capillary samples were acquired before running the process controls. In stability, samples with two different time points from the same specimen type were acquired using different FACSPresto instruments. For linearity, a low level sample did not followed the predefine order during acquisition. The protocol deviations were minor and had minimum impact on the quality of the data.

## Discussion and Conclusions

This is the first evaluation of the performance of the BD FACSPresto Near-Patient CD4 Counter after its introduction in the market with the intent of increasing access to CD4 testing and services in resource-limited settings. The BD FACSPresto Near-Patient CD4 Counter, along with other point-of-care devices, enables the collection and testing of samples in the local community and overcomes time or geographical constraints. The system is a response to the UN Millennium Development Goals to improve maternal health, child health, and combat HIV/AIDS in developing countries [37]. The point-of-care CD4+ cell counter(s) currently on the market use venous and capillary blood [13, 14, 38, 39] to enumerate CD4 absolute count in human whole blood.

AbsCD4 is a widely accepted surrogate marker for the decision to start ART in adolescent and adult patients. For children under 5 years of age, results from %CD4 are considered a more reliable surrogate marker due to normal lymphocytosis at that age range [15]. The BD FACSPresto system provides results for both AbsCD4 (cells/μL) and %CD4 of a T-cell lymphocyte population. These parameters are used for monitoring HIV-infected populations that have no access to viral load monitoring and are also used for monitoring children and subjects with co-infections, opportunistic infections, or treatment failure [40, 41]. Determination of CD4 cell count is relevant for rapid monitoring of these subjects in their community using CD4-point-of-care devices. The BD FACSPresto Near-Patient CD4 Counter also includes a third analytic parameter, measurement of the concentration of Hb (g/dL or g/L) from the same sample, which is an important marker for anemia as a prognostic factor in HIV infection (Fig 2 and S1 Fig).

Anemia is a commonly encountered hematological abnormality that has a significant impact on clinical outcomes of the patients [17, 19]. ZDV is known for hematological adverse effects that include anemia, neutropenia, and macrocytosis [17, 42]. Determination of the concentration of Hb may be an indicator for early detection of anemia in patients receiving ART combination therapy that includes ZDV [42], and for monitoring of HIV-infected pregnant women and children. Several studies have shown that Hb concentration reflects the rapidity of disease progression rates and independently predicts prognosis across demographically diverse populations [18, 19]. The rate at which Hb decreases also correlates with falling CD4 counts [43], suggesting that increases in Hb are predictive of treatment success [44, 45]. Integration of these three parameters in the BD FACSPresto Near-Patient CD4 Counter is intended for more integrated and comprehensive routine monitoring of HIV/AIDS patients in resource-limited settings using capillary blood.

Schalk et al [46] reported differences in Hb and hematocrit capillary measurements. However, Daae et al found equivalency of the cellular components between venous and capillary blood [47]. Recently, it was reported that CD4 results were comparable to the predicate when the cartridges were filled using a pipette, but not for capillary blood transfer [48]. A study by Sitoe et al [49] showed that venous and capillary blood enumeration of both CD4+ T-cell absolute count and %CD4 using the BD FACSCalibur and BD FACSCount^™^ systems was similar. The FACSPresto mean bias results in Tables 2 and 3, Fig 2 and S1 Fig lead to similar conclusions, the numeric values of the mean bias and Bland-Altman mean bias are closer. In addition, the mean %sim for AbsCD4, %CD4 and Hb showed the close agreement of the FACSPresto with the predicate methods in venous and capillary blood results (Fig 2 and S1 Fig), demonstrating equivalency for CD4 determination. The challenge with CD4 determination in capillary blood is that the sample must be whole blood to provide valid results (Fig 3). Therefore, validation of the capillary blood collection method, the device selected for finger-stick, and the steps to efficiently transfer the drop of blood onto the cartridge are fundamentally important to obtain accurate results (Fig 2). The capillary blood collection method used with the patients participating in the studies was a reproducible and robust method, since our results from these two specimen types were equivalent as shown in the results from the accuracy (Fig 3), stability and reference interval studies.

The clinically relevant AbsCD4 cutoff in venous blood of 350 cells/μL was used to analyze overall positive or negative agreement. The BD FACSPresto overall agreement for AbsCD4 was 95.8%; positive agreement was 88.0%, and negative agreement was 98.6% (Table 5). Analyses at different AbsCD4 cutoffs (Table 6) provide additional information showing that the BD FACSPresto’s overall agreement for identifying patients eligible for treatment in both venous and capillary blood was ≥94.4%, and for %CD4 was ≥90.1%. Negative agreement for AbsCD4 was ≥95.8% for all cutoffs with exception of capillary blood at the 750 cells/μL cutoff. For %CD4, the positive agreement was ≥88.8% and the negative agreement was ≥90.7% for both venous and capillary blood. These results show acceptable agreement of the BD FACSPresto system with the AbsCD4 predicate FACSCalibur/BD Tritest method. However, at a cutoff of 200 cells/μL, the number of subjects was small. Therefore, additional studies would be required to address misclassification at this clinically relevant cutoff frequently used for monitoring subjects with co-infections or opportunistic infections [40, 41]. Other authors have used similar analyses presented as sensitivity and specificity for other point-of-care products in the market [13, 14, 16, 38, 39, 50].

The results from Hb concentration in venous blood show the concentration of Hb in Bland-Altman plots (Fig 2E and 2F). Although most of patient data fell within the normal ranges, the graph also depicts results with larger variability either below 8 or above 17 g/dL. These results correspond to manipulated samples (n = 30) used to fill the Hb bins. In addition, regression analysis also showed equivalency with the reference method (Figs 2 and 3, S1 Fig).

Our stability results support using venous blood anticoagulated with EDTA within 24 hours and capillary blood within 2 hours of draw. Our results showed more variation reflected as larger mean %bias for AbsCD4 and %CD4 at about 24 hours post-draw (Table 8). Coincidentally, we observed an increase in number of cartridge re-runs at about the 24-hour time point, along with more errors (6B0F). However, the majority of the errors were resolved after re-running the cartridge.

The BD FACSPresto reference interval results from the healthy cohort showed statistically significant gender differences for each parameter (Table 12). Females had higher AbsCD4 and %CD4, and males had higher Hb values. Statistically significant Hb gender differences are well known and established [51]. However, it was surprising encountering similar gender differences in results for AbsCD4 and %CD4 in this cohort, since this was not observed in a previous study [51] and further studies are necessary to established valid reference intervals for the FACSPresto cartridge.

Precision studies of the BD FACSPresto Near-Patient CD4 Counter and the cartridge have shown that the performance of the system is highly reproducible and robust (Tables 9 and 10) for all three parameters in the different conditions tested. Regarding BD FACSPresto linearity, total lymphocytes, AbsCD4, and Hb data has shown that the cartridge performance linear ranges for these three parameters comply with the product claim ranges (Table 11), data to be provided upon request. For the purpose of this evaluation, the algorithm embedded in the BD FACSPresto collected and analyzed total lymphocyte and AbsCD4 count to report %CD4. The software version used during data collection made available total lymph results for linearity testing. Typically this information would not be available to the targeted end-user in resource-limited setting health clinics, since calculation of the total lymphocytes has no clinical significance for HIV/AIDS monitoring or treatment.

On the other hand, during our evaluation, the laboratory operators found that the BD FACSPresto was easy to use with a friendly customer interface, and simple to navigate with supporting self-training materials for the different steps, from instrument QC, blood collection, sample acquisition, to results handling.

The BD lancet used during the clinical evaluation has been marketed worldwide. This is a safety engineered and reliable lancet. During capillary blood collection, there were a minimum number of BD lancet failures (<1%) for the duration of the studies. In addition, capillary blood was effectively and easily transferred directly onto the cartridge after a short training. The simplicity of this procedure was reflected in our capillary blood results, which support the AbsCD4, %CD4, and Hb equivalency with venous blood. Regarding sample throughput, the cartridge incubation is staged outside the instrument on incubation tray(s) or in an incubation workstation. The BD FACSPresto throughput is conservatively estimated to be 10 cartridges per hour, with approximately 23 minutes from blood application to results. Despite the constraints of the clinical evaluation, the laboratory staff was able to run an average of 50 cartridges in two instruments, including process controls, cartridge re-runs, and cartridge re-preparation, suggesting that at the sites where the BD FACSPresto may be placed, it would be expected to facilitate a rapid turnaround of the results.

Although performance characteristics should be a high priority with any new diagnostic test, the impact of the BD FACSPresto Near-Patient CD4 Counter tests on treatment monitoring and linkage to care should be considered, since this outweighs the effect of the tests' performance characteristics within reasonable ranges. It has been shown that there is little added value in long-term CD4 monitoring among virally suppressed patients, and stopping CD4 monitoring will have major cost savings [52–54]. Therefore, efforts are being made to discontinue CD4 testing in situations where viral load testing is available and patients are virologically suppressed.

However, CD4 cell counts, the prime indicator of HIV disease progression, can expedite the process leading from HIV diagnosis to ART and improve clinical outcomes, since they still continue to play an important part in initial decisions around ART initiation. To meet the 90-90-90 targets [55], CD4 cell counts will continue to be used as a filter mechanism to prioritize patients in need of treatment and clinical management. UNAIDS highlights that the current viral load scale-up is at 25% and, it is expected to reach only 57% by 2019. Therefore, to meet the 2020 UNAIDS target of 90% virally suppressed patients, CD4 will continue to play a role for countries lacking viral load testing capacity. This is recommended by WHO [56] for treatment monitoring when viral load monitoring is limited and in cases in which the CD4 cell count of a few patients might fail to increase despite virological suppression.

Near-patient and point-of-care CD4 tests can help more people reach care, since they offer a way of providing portable capacity for rapid CD4 testing in settings where traditional CD4 testing using flow cytometry cannot be implemented. Therefore, availability of the BD FACSPresto Near-Patient CD4 Counter in remote or resource-limited areas will aid in prompt clinical management, especially for patients presenting late to care, those returning to care after a period of treatment interruption, and those experiencing virological or clinical failure. Much of this improvement is due to increased test completion and receipt of results.

Determination of CD4 cell count has an important role in decisions for screening and prophylaxis for major opportunistic infections. There is a continuous need to monitor the CD4 count to determine whether patients require prophylaxis or not, and when to stop. Shubber et al [57] have shown that baseline CD4 measurement might have a role in decisions around use of nevirapine, in view of the potential increased risk of nevirapine-associated hypersensitivity reaction at increased CD4 cell counts. Attention must therefore remain focused on sustaining improvements in any of the sequential steps of linkage after point-of-care CD4 testing. It is important to identify cost-effective methods for immunologic staging that will expedite access to care for the high-priority cases of the most immunosuppressed individuals in resource-limited settings.

In summary, the data suggests good agreement between the BD FACSPresto Near-Patient CD4 Counter and laboratory-based CD4 and Hb testing, the BD FACSCalibur and the Sysmex KX-21N. We find that devices such as the BD FACSPresto Near-Patient CD4 Counter for CD4 testing have the potential benefit of expanding timely access to testing, increasing timely initiation of ART, providing the correct regimen, establishing a baseline enumeration for patients beginning treatment, simplifying ART services at the primary care level, and reducing patient loss to follow-up. This strategy can stop deaths and offer excellent value for immunologic staging across a wide range of parameters in Kenya, as well as in a variety of resource-limited settings. The results of the BD FACSPresto evaluation are compliant with the Transparent Reporting of Evaluations with Non-randomized Design (S8 File). Further research is required on the operational aspects of the BD FACSPresto Near-Patient CD4 Counter and other devices defined as point-of-care CD4 testing services.

## Limitations

Data from this study should be interpreted in light of a few important limitations. The study was conducted in a single research facility, and while this facility is broadly representative of HIV research facilities in this setting, the results may not be generalizable to other facilities within the country. The CD4 tests were done by laboratory staff. CD4 monitoring level 2 to 4 health facilities will require strong capacity building among staff and constant supervision to ensure high quality sample collection and testing. There may be divergence in agreement between BD FACSPresto instruments and operators, and this warrants further consideration.

## Conclusions

The BD FACSPresto Near-Patient CD4 Counter is a robust, reliable, and easy to use system that provides accurate, robust, precise and linear clinical results for capillary or venous samples. The BD FACSPresto is a suitable alternative requiring short user training for optimally generating CD4 and Hb integrated results in health care facilities in resource-limited settings. This product was CE Marked (IVD Directive 98/79/EC) and WHO prequalified (2014).

## Supporting Information

S1 Fig AbsCD4, %CD4 and Hb Deming Regression Results in Venous and Capillary Blood. Regression analysis with Deming fit of venous and capillary blood samples. In the left side shows results from venous blood (A, C and E), and the right side illustrates capillary blood (B, D and F) plots. The plots at the top depict the AbsCD4 results (A and B); %CD4 results are shown in the middle(C and D), and the Hb plots are at the bottom (E and F) with R^2^, slope, and y-intercept data. The BD FACSCalibur results are represented in the x-axis and the BD FACSPresto results in the y-axis.
(TIF)

S1 File Accuracy, Stability and Reference Intervals Protocol. (PDF)

S2 File Precision protocol. (PDF)

S3 File Linearity protocol. (PDF)

S4 File Accuracy data. (XLSX)

S5 File Stability data. (XLSX)

S6 File Linearity data. (XLSX)

S7 File Reference intervals data. (XLSX)

S8 File FACSPresto TREND Checklist. (PDF)

S1 Table Clinical Evaluation Inclusion/Exclusion Criteria. (DOCX)
