# Supplementary figures and images for: Clinical Evaluation of the BD FACSPresto™ Near-Patient CD4 Counter in Kenya
Source: PLoS One. 2016 Aug 2;11(8):e0157939. doi: 10.1371/journal.pone.0157939 (PMC4970792; doi:10.1371/journal.pone.0157939)

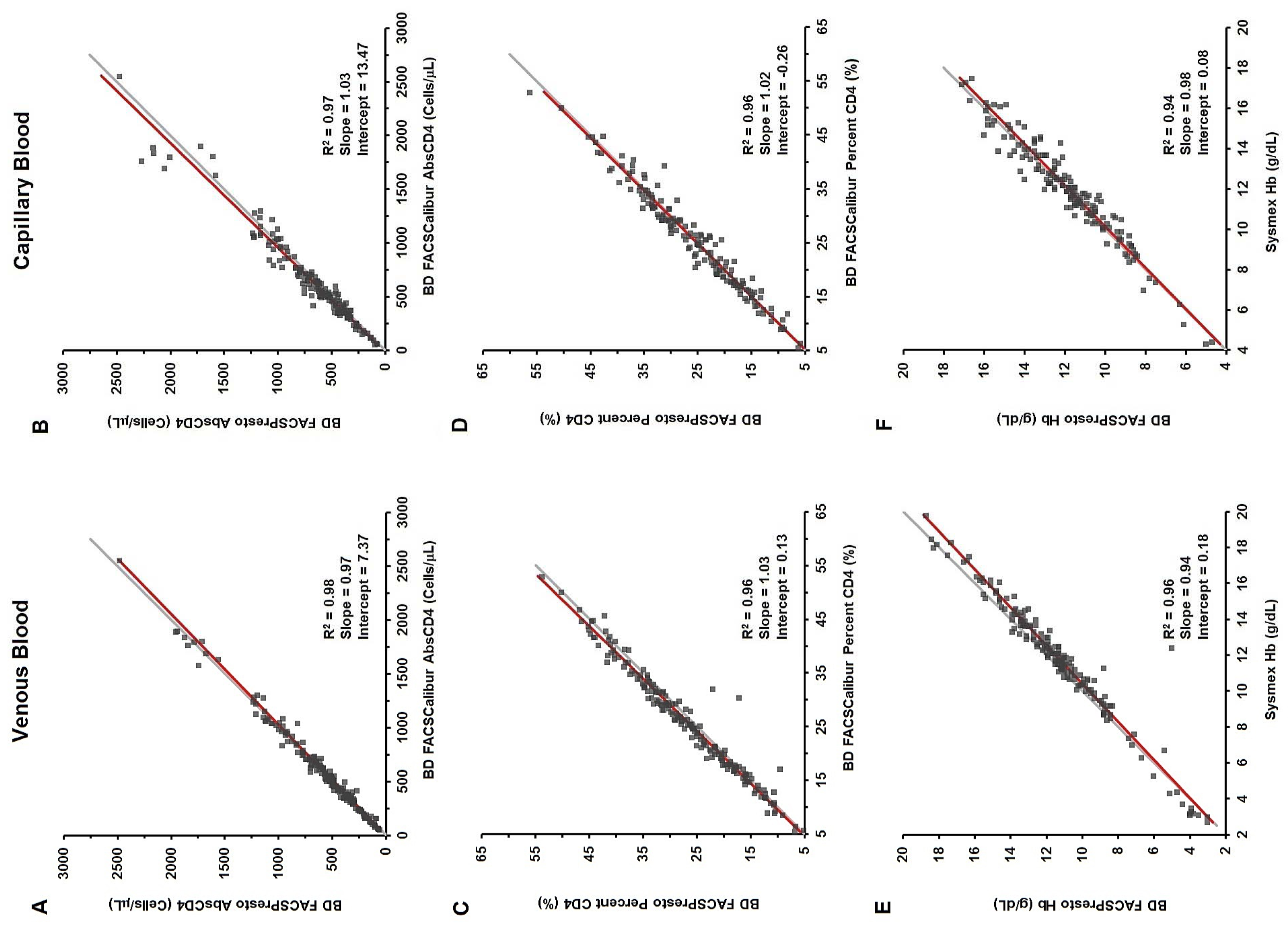

Supplement: S1 Fig — Regression analysis with Deming fit of venous and capillary blood samples. In the left side shows results from venous blood (A, C and E), and the right side illustrates capillary blood (B, D and F) plots. The plots at the top depict the AbsCD4 results (A and B); %CD4 results are shown in the middle(C and D), and the Hb plots are at the bottom (E and F) with R2, slope, and y-intercept data. The BD FACSCalibur results are represented in the x-axis and the BD FACSPresto results in the y-axis. (TIF) [file pone.0157939.s001.tif]
