# Supplementary material for: Clinical Evaluation of the BD FACSPresto™ Near-Patient CD4 Counter in Kenya
Source: PLoS One. 2016 Aug 2;11(8):e0157939. doi: 10.1371/journal.pone.0157939 (PMC4970792; doi:10.1371/journal.pone.0157939)
Supplement: S1 File — (PDF) [file pone.0157939.s002.pdf]

Evaluation of the Investigational BD FACSPresto™ System: Instrument, Software, and BD CD4%CD4/Hb Cartridge Assay

**Sponsor**	BD Biosciences<br>2350 Qume Drive<br>San Jose, CA 95131
**Conducted by:**	KEMRI/CDC Research & Public Health Collaboration<br>HIV-Research laboratory<br>P.O. Box 1578<br>Post code 40100<br>Kisumu, Kenya
**Version**	**5.0**
**Date**	**26th March 2014**

For Investigational Use Only

The Performance characteristics of this product have not been established.

All investigational material supplied by BD Biosciences must be used for study purposes only. Results from investigational testing must not be used as a diagnostic procedure without confirmation of the diagnosis by another medically established diagnostic product or procedure.

Confidential

The product information and data disclosed through this study protocol are confidential and are subject to a Confidential Disclosure Agreement. No content may be disclosed without prior written consent of Becton, Dickinson and Company.

TABLE OF CONTENTS

LIST OF ABBREVIATIONS AND ACRONYMS 5

LIST OF ABBREVIATIONS AND ACRONYMS 6

STUDY INVESTIGATORS AND ROLES.. 7

PROTOCOL SUMMARY 9

1.1 Statement of Compliance 11
1.2 Background Information and Scientific Rationale 11
1.3 Clinical Validation and Evaluation 12
1.4 Proposed Intended Use 14

2.0 OBJECTIVES AND DESIGN 14

2.1 Primary Objective(s) 14
2.2 Secondary Objective(s) 15
2.3 Overall Study Design / Outcome Measures 15
2.4 Expected Study Duration 16
2.5.1 Investigational Device(s) 17
2.5.2 Predicate Devices 17

3.0 STUDY POPULATION 17

3.1 Description of Study Site 17
3.3 Inclusion Criteria 18
Capillary blood applied onto **the PEO/IUO BD CD4/%CD4/Hb cartridge. 3.4 Exclusion Criteria** 18
3.5 Sample Size Determination 18

4.0 ACCURACY STUDY GENERAL METHODS AND PROCEDURES 19

4.1 Daily Study Activities 19
4.1.1 Subject Enrollment 19
4.1.2 Venous Blood Specimen 21
4.1.3 Capillary Blood Collection 21
4.1.4 Enrollment by Subjects Age 21
4.1.5 Enrollment by CD4 Count 21
4.1.6 Enrollment by Hb (only venous blood) 22
4.1.7 Instrument Setup/Instrument QC 23
4.2 Enrolled Specimen Testing 26
4.2.1 BD FACSPresto Method: Sample Preparation 26
4.2.2 Predicate FACSCalibur™ Method 27
4.2.3 Predicate Hb Method 27
4.2.4 Enrolled Sample Acquisition and Analysis 28

5.0 DISCONTINUATION OF SPECIMEN TESTING **30**

6.0 PRODUCT ERRORS/ DEFECTS/ FAILURES **30**

7.0 DATA COLLECTION AND MANAGEMENT **30**
7.1 Data Collection and Source Documents 30
7.2 Submission of Data Packages to Sponsor 31
7.3 Data Evaluation for Inclusion/Exclusion from Analysis 32
7.4 Data Management and Storage 33

8.0 STATISTICAL METHODS **33**
8.1 Sample Size Determination 33
8.2 Eligibility, Exclusions, Missing Data, Interim Analysis 33
8.3 General Statistical Considerations 33
8.4 Study Endpoints / Outcome Measures 33
8.4.1 Primary Endpoint 34
8.4.2 Secondary Endpoint 34
8.5 Acceptance Criteria 34
8.6 Methods to Reduce Bias 35
8.7 Analysis Method for the Accuracy Evaluation Procedure 35
8.8 Biostatistics Report 37

9.0 STUDY MATERIALS AND SUPPLIES **37**
9.1 Investigational Product Labeling 37
9.2 Study Products Shipment and Receipt 37
9.3 Study Product Accountability 37
9.4 Required Study Materials and Supplies 38

9.5 Biohazard Containment **39**
9.6 Disposition 39

11.0 SAFETY REPORTING **42**
12.0 STUDY SUSPENSION OR TERMINATION 42

13.0 STUDY ETHICS/ GOOD PRACTICES **43**
13.1 Ethics Review and Approval 43
13.2 Informed Consent 44
13.3 Risks and Benefits 44
13.4 Donor Identification and Confidentiality 44
13.5 Donor Honoraria 45
14.0 INVESTIGATOR 45
15.0 SPONSOR 47
16.0 PUBLICATION 48

17.0 REFERENCES 48

18.0 BUDGET 49

APPENDICES 51

APPENXIX 1 – STUDY SITES 52

APPENDIX 2: STUDY SCREENING AND ELIGIBILITY FORM 53

APPENDIX 3: ENROLLMENT QUESTIONNAIRE FOR ADULT AND CHILDREN 54

APPENDIX 4: ADULT INFORMED CONSENT FORM (CONSENT 01) 55

APPENDIX 5: PARENTAL/GUARDIAN CONSENT FORM (CONSENT 02) 59

APPENDIX 6: MINOR ASSENT FORM (ASSENT 03) 64

APPENDIX 7: DONOR'S QUESTIONNAIRE 68

APPENDIX 8: RELEASE OF MEDICAL INFORMATION FOR ADULTS (ENGLISH) 70

APPENDIX 9: RELEASE OF MEDICAL INFORMATION FOR CHILDREN (ENGLISH) 71

APPENDIX 11: ADULT INFORMED CONSENT FORM (CONSENT 04) 74

APPENDIX 12: PARENTAL/GUARDIAN CONSENT FORM (CONSENT 05) 78

APPENDIX 13: MINOR ASSENT FORM (ASSENT 06) 82

LIST OF ABBREVIATIONS AND ACRONYMS

%CD4	Percentage of CD4+ T lymphocytes of total lymphocytes
Abs CD4	Absolute cell count (cells/mL) of all T lymphocytes that are CD4+
AIDS	Acquired Immunodeficiency Syndrome
AOB	Age Of Blood is the time period from venipuncture draw to the start of staining
AOS	Age Of Stain is the time period from sample lyse to acquisition
ART	Antiretroviral therapy
BD	Becton Dickinson and Company
BDB	BD Biosciences, hereafter referred to as sponsor
CBC	Complete blood counts
CD3	Antigen present on all T lymphocytes
CD4	Antigen present on the "helper/inducer" T lymphocyte subpopulation
CI	Confidence Interval
CE	Clinical Evaluation
CRA	Clinical Research Associate
CRF	Case Report Form
DCF	Data Clarification Form
FIO	For Information Only
ERC	KEMRI Ethics Review Committee
FDA	Food and Drug Administration
GCP	Good Clinical Practice
HIV	Human Immunodeficiency Virus
ICH	International Conference on Harmonization for Registration of Pharmaceuticals for Human Use; as adopted by the FDA (i.e., Good Clinical Practice E6)
ICF	Informed Consent Form
IRB/IEC	An Institutional Review Board or Independent Ethics Committee
IUO	Investigational Use Only
IVD Performance Evaluation Study	An investigation of the performance of an *in-vitro* diagnostic medical device based upon data already available, scientific literature and / or performance evaluation studies[*]
IVD medical device	Any medical device which is a reagent, reagent product, calibrator, control material, kit, instrument, apparatus, equipment, or system, whether used alone or in combination, intended by the manufacturer to be used *in vitro* for the examination of specimens, including blood and tissue donations, derived from the human body, solely or principally for the purpose of providing information: concerning a physiological or pathological state, or concerning a congenital abnormality, or to determine the safety and compatibility with potential recipients, or to monitor therapeutic measures.[†]
PE**O**	Performance Evaluation Only

[*]EN 13612:2002, Performance evaluation of in vitro diagnostic medical devices

[†]98/79/EC:1998, Directive on *in vitro* diagnostic medical devices

LIST OF ABBREVIATIONS AND ACRONYMS

POC	Point of Care
Predicate or Predicate Method	The previously approved or generally accepted method
PI	Principal Investigator
QC	Quality Control
SSC	Scientific Steering Committee
UADE	Unanticipated Adverse Device Effects
WB	Peripheral Whole Blood
WBC	Whole blood counts
WHO	World Health Organization

STUDY INVESTIGATORS AND ROLES

Name	Role	Contribution
Clement Zeh, Ph.D.	Principal Investigator,<br>Laboratory Director,<br>HIV Research Branch,<br>KEMRI/CDC (Kisumu)	Substantial input into study design, protocol development and assessment tools. Will provide laboratory oversight and input for study implementation and report writing and supervise Kisumu-based study staff. Will have no involvement with identifiable data or specimens, and will have no interaction with participants.
Lisa A. Mills, MD, MSc	Chief, HIV Research Branch,<br>KEMRI/CDC (Kisumu)	Will provide technical support, data analysis and authorship, but will have no involvement with identifiable data or specimens, and will have no interaction with participants.
Frank Angira, HND, MPH (c)	Sr. Clinical Officer, Study coordinator, HIV Research Branch, KEMRI/CDC (Kisumu)	Will coordinate and oversee daily study operations and implementation and supervise Kisumu based study staff.
Benta Akoth, HND	Laboratory technologist, in-charge of CD4 and Hematology monitoring	Will oversee daily laboratory testing and implementation of Point care devices
Boaz Oyaro, HND	Laboratory Manager,<br>HIV Research Branch,<br>KEMRI/CDC (Kisumu)	Will oversee overall daily laboratory testing and implementation of Point care devices
Kayla Laserson, PhD	Field station director<br><br>KEMRI/CDC (Kisumu)	Involved in protocol development and review. Will provide input for study implementation.
John Vulule, PhD	Centre Director, Centre for Global Health Research,<br>KEMRI (Kisumu)	Involved in protocol review
Kevin Judge, MD	Vice-president, of Medical Affairs<br>Phone: 201-847-4454<br>Fax: 201-523-9042<br>Email: kevin_judge@bd.com	BD Medical Safety Officer will oversee safety and medical risks of the BD sponsored clinical study ensuring that considerations have been given to minimize risks to subjects participating in the study.

Lorlelei Lee-Haynes, MPH	Clinical Operations<br>Director of Clinical Operations<br>Phone: 408-954<br>Fax: 408-954-6313<br>Email: lorlelei-lee_haynes@bd.com	Will oversee resourcing, planning, conduct and reporting of results of the BD sponsored clinical studies.
Imelda Omana-Zapata, MD, PhD	Clinical Operations<br>CPL / Study Manager<br>Clinical Project Lead<br>Phone: 408-954-2323<br>Fax: 408-954-6313<br>Email: Imelda_omana-zapata@bd.com	Will coordinate, develop and maintain clinical plan and ensure progress of the clinical plan and timely attain of deliverables
Jerry Zhang, PhD	Biostatistics<br>Biostatistician<br>Phone: 408-954-6301<br>Email: jerry_q_zhang@yahoo.com	Will provide input on the study design and analyze the data.
Jeannine Paliotta, BSc	Data Management<br>Data Manager<br>Phone: 408-954-4111<br>Email: Jeannine_Paliotta@bd.com	Will lead activities associated with database set-up, data processing in compliance with the protocol and internal procedures
Henok Tilahun MSc	Product line manager for FACSPresto System<br>Phone: 408-954-2436<br>Email: Henok_Tilahun@bd.com	Will provide technical input on the study product, coordinate and maintain clinical plan, oversee resourcing, conduct and reporting of results of the BD sponsored clinical studies.

PROTOCOL SUMMARY

Purpose:

To evaluate the feasibility and accuracy of the investigational BD FACSPresto™ System including the instruments Software and BD CD4% CD4/Hb Cartridge Assay in determination of Absolute CD4, percentage of CD4, and Hemoglobin (Hb) concentration using venous blood and capillary blood.

Design:

This study is designed to encompass three phases: I Feasibility, II Customer Evaluation, and III Regulatory Submission for CE-IVD and Regulatory Submission for FDA 510K. The study will involve prospective evaluations based on the CLSI guideline EP9-A2 on method comparison and bias estimation using patient samples at a site external to BD in first three phases and at three sites in the fourth phase.

Population:

A minimum total of **550** HIV infected participants: infants (0-5 yr), children (6-12) and adolescent/adult subjects aged ≥ 13 years that provide venous and capillary specimen with valid results. Specimens must also fulfill study specific age and CD4/Hb binning requirements.

Study Duration:

The study duration is approximately 28 weeks, from participant enrollment to completion of testing.

Primary Objective:

The Objectives and -Endpoints refer to phase III; Regulatory submission for CE-IVD and US 510K Submission for market clearance. .

The primary objective for the Performance Evaluation procedure is to determine the method bias between the "For Performance Evaluation Only" (PEO))/ "Investigational Use Only" (IUO) labeled BD FACSPresto system versus the predicate systems (IVD Tritest™ CD3/CD4/CD45 reagent with Trucount™ tubes and BD Multiset software on the FACSCalibur™ flow cytometer) in the determination of Abs CD4 and%CD4, using venous blood in de-identified specimens at three or more external study sites.

Primary Endpoints:

The primary endpoint is the bias (expected difference) between the investigational system versus the predicate system measured as Abs CD4 and %CD4 in peripheral venous blood specimens from a minimum of 400 specimens from adolescent/adult (subjects aged ≥ 13 years) with valid results tested at three or more sites. .

Secondary Objectives:

Determine method bias between the PEO/IUO labeled BD FACSPresto System versus the predicate system in the determination of Abs CD4 and %CD4, using capillary blood in de-identified specimens at three or more external study sites.

Determine the method bias between the PEO/IUO BD FACSPresto System versus the predicate system for Hb, Sysmex (KX-21) hematology analyzer, in the determination of Hb concentration using venous blood and capillary blood in de-identified specimens

Evaluate the customer's ease of use of the IUO PEO/IUO BD FACSPresto System following brief training.

Secondary Endpoints:
Secondary endpoints have been defined for the analysis of the AbsCD4, %CD4 and Hb results from investigational BD FACSPresto System and predicate methods using capillary and venous blood.

- **Evaluation of CD4 and Percentage of CD4 in capillary blood**
  Expected difference between the investigational system using capillary blood specimens versus the predicate system using venous blood specimens measured as AbsCD4 and %CD4.

- **Evaluation of Hb concentration in venous and capillary blood**
  Expected difference between the investigational system testing capillary blood specimens versus the predicate system using venous blood specimens measured as Hemoglobin concentration.

- **Enrolled Sample Acquisition and Analysis**
  The customer's response on the "ease of use" is a qualitative and quantitative assessment of how quickly, easily and safely a completely instrument-naïve operator should be able to use the PE/IUO BD FACSPresto system, after a 3 hrs. or less training period and the "anxiety" factor as to fear of breaking something or losing a large amount of patient data without noticing it.

1.0 INTRODUCTION

1.1 Statement of Compliance

The study is to be conducted in compliance with this protocol, Good Clinical Practice (GCP)[1], the Declaration of Helsinki, Title 21 of the Code of Federal Regulations §§ 50, 56, and 812, International Conference on Harmonization E6 and ISO Standards 14155:2011 .

1.2 Background Information and Scientific Rationale

The enumeration of T lymphocytes that are positive for the CD4 antigen is used to evaluate the immune status of patients with, or suspected of developing, immune deficiencies such as AIDS. The CD4 antigen is the receptor for Human Immunodeficiency Virus (HIV) and the number of CD4+ T lymphocytes declines due to programmed cell death in an HIV infection[2]. The absolute count of CD4+ T lymphocytes (Abs CD4) and the percentage of CD4+ T lymphocytes of total lymphocytes (%CD4) are cellular parameters closely associated with HIV disease progression and patient prognosis. %CD4 measurement is particularly important in the context of infant patient management and in the context of a wide range of co-morbidities. According to World Health Organization (WHO) guidance documents regarding HIV/AIDS patient treatment, the measurement of Abs CD4 cell counts and percent of CD4+ in adolescents and adults[3,4] is recommended for decision-making on initiating antiretroviral therapy (ART). Recently, the use of ART has been extended on prevention or profilaxis.[5,6]

Anemia can be induced by ART[4,7] or one of the hematological abnormalities frequently observed in HIV/AIDS patients in developing countries[8]. Anemia is usually diagnosed by measuring the concentration of hemoglobin (Hb) and by counting the number of red cells or hematocrit in whole blood. It is a common practice during the patient's routine visit, more so in pregnant women, to evaluate presence of anemia and other hematological or functional parameters to assess HIV/AIDS disease progression or control.

In the recent past, there has been an expansion of laboratory infrastructure in many developing countries. Despite this, access to CD4 testing remains a bottle neck to provision of care and treatment to HIV patients due to increased demand for CD4 tests arising from ART scale up in these regions, as well as most CD4 testing being done in few central laboratories. This often leads to delays in CD4 testing and significantly contributes to loss of patients, either who fail to return for their results or who die before initiating treatment, hence negating the gains made in the scale up of antiretroviral therapy in these areas. Attempts to overcome these huddles have lead to the development of point of care systems that can offer simple, effective and scalable means of measuring these parameters within a shorter turnaround time. This approach would allow patients to receive their CD4 and other hematological test results and possibly be put on therapy on the same day of testing.

The recent development of PIMA point-of-care (POC) CD4 test system (Alere, Waltham, MA) conducts CD4 tests within 20 minutes of sample collection using capillary blood. The test can be conducted by non-laboratory staff, and testing does not require laboratory infrastructure[12]. However, this device provides only the absolute CD4 counts, leaving the quest for other critical parameters key in management of infant and pregnant women patients like %CD4 and Hb unattained.

The BD Point of Care CD4 System is an investigational, robust, affordable and portable CD4 and Hb system consisting of an instrument with fluorescence and light absorbance microscopy and embedded software; dried reagent cartridges with reagent quality controls; and instrument quality control. The BD Point of Care CD4 first generation will serve customers in resource limited settings of the developing

world, designed to increase access to treatment by offering accurate, timely and reliable tests that support staging and monitoring of all CD4/Hb patients, encompassing adolescents, adults as well as pregnant women and infants living in resource-limited settings of developing countries.

1.3 Clinical Validation and Evaluation

The purpose of the Clinical Validation of the PEO Only/IUO BD FACSPreso™ CD4 System (including PEO/IUO BD CD4% CD4/Hb cartridge) is to plan, prepare and conduct clinical studies and present the results of the studies to support Regulatory submissions and product marketing. This study encompasses three phases: I Feasibility, II Customer Evaluation, III Regulatory Submission for CE-IVD and Regulatory Submission for FDA 510K.

KEMRI/CDC has been identified as suitable clinical study site to participate during the four phases of the PEO/IUO BD Point of Care CD4 System Clinical Validation and Evaluation. All phases of the study are briefly depicted below:

I. Feasibility Phase

This phase includes Accuracy evaluation using prototype PEO/IUO BD CD4 System and prototype PEO/IUO CD4/Hb cartridge. BD will lead the study, site staff will be involved in a supporting role. All data collected will be used by BD to complete characterization according to predefine requirements and performance evaluation of the system and cartridge.

Study Summary:

1. Prototype system will be used that includes: Instrument, embedded software, reagents, instrument QC, standards and the new BD lancet
2. Sample size: Minimum of 200 venous and 200 capillary blood specimens that provide valid results
3. BD staff will be at the site working with the proto-type PEO/IUO BD FACSPresto™ System and PEO Only/IUO CD4/Hb cartridge and KEMRI/CDC Site staff will support flow cytometry testing and Hb testing with Sysmex hematology analyzer (KX-21) .
4. Hb will be fully integrated into the study work flow
5. SSC/ERC to provide written approval to conduct protocol and informed consent and Study Agreement/Contract
6. Collect basic patient demographic information
    a. Age, gender, co-morbid conditions (anemia, TB, malaria, other)
7. Estimated start date: July, 2013
8. Study Systems:
    - Prototype PE) Only/IUO BD FACSPresto™ System and PEO/IUO CD4/CD4% CD4/Hb Cartridge
    - CD4 predicate= FACSCalibur/Tritest
    - Hb predicate= Sysmex hematology analyzer (KX-21)

II. External Customer Evaluation

This study will be conducted after the Feasibility and before or during the Single Site Accuracy Study.

Prospective single site planned for July, 2013. BD Biosciences protocol number CAS-PCMKT 1

Study Summary:

1. Single external site in Africa

2. Sample size: A minimum of 50 capillary and 50 venous blood specimens that provide valid results.
3. 2-3 week duration (assumes 14-18 venous and 14-18 capillary specimens/week).
4. Assumes study will be conducted by Principal Investigator and delegate responsibilities to 1 full time KEMRI/CDC Site staff member (testing and administrative functions).
5. EC/IRB to provide written approval to conduct protocol and informed consent.
6. Collecting basic patient demographic information.
    a. Age, gender, co-morbid conditions (anemia, TB, malaria, other)
7. Conducted under Good Clinical Practices and GLP.
8. Under BD Oversight.
9. Estimated start date: July, 2013
10. Study Systems:
    - Production equivalent PE**O**/IUO BD CD4 System and PE**O**/IUO CD4/%CD4/Hb Cartridge
    - CD4 predicate= FACSCount/ Tritest
    - Hb predicate= HemoCue 301

III. <u>Regulatory US FDA 510K Submission for market clearance</u>

For this phase, three activities are planned, the Accuracy, Reference interval and Stability evaluation of the PEO/IUO BD FACSPresto System and PEO/IUO cartridge. All the activities will be carried out by the KEMRI/CDC Investigator, Accuracy as multisite and Stability as two-site studies.

Accuracy prospective multisite study planned for August – October 2013, 2013. BD Bioscience protocol number CAS-PCACC1.

Stability prospective single site study planned for August - October, 2013. BD Bioscience protocol number CAS-PCAOB.

Reference Intervals prospective single site study planned for August-October, 2013. BD Bioscience protocol number CAS-PCREF.

Study Summary:

1. Clinical studies will be conducted under GCP/GCLP guidelines and data will be used for FDA 510K regulatory submission.
2. Accuracy: Multisite prospective study
    a. Three external or more sites (3OUS)
    b. Total cohort = A minimum of 400 specimens (400 capillary and 400 venous blood (+20%)) that provide valid results
    c. Three external sites (1US/2OUS):
        i. Site 1 (OUS) = 133 capillary and 133 venous blood specimens
        ii. Site 2 (OUS) = 134 capillary and 134 venous blood specimens
        iii. Site 3 (US) = 133 capillary and 133 venous blood specimens
    d. Duration: 16 week assuming sites start at different time; specimen processing also assumes daily testing of 10 venous and 10 capillary specimens during five weeks processed at each study site.
    e. Assumes 2 full time staff members (testing and administrative functions) at each site.
    f. EC/IRB with approved informed consent and Study Agreement.

g. BDwill monitor sites and data
h. Estimated start date: August, 2013
i. Systems
    i. PEO/IUO BD CD4 System and PEO/IUO CD4/Hb Cartridge
    ii. CD4 predicate= FACSCalibur/ Tritest
    iii. Hb predicate= Sysmex analyzed KX-21

3. Stability: Singlesite prospective study (1OUS)

    a. 40 capillary and 40 venous blood specimens that provide valid results
    b. 2 number of staff members (testing and administrative functions)
    c. IRB with approved informed consent and Study Agreement
    d. Conducted under Good Clinical Practices and GLP
    e. BD will monitor sites and data
    f. Estimated start date: September, 2013
    g. Systems
        i. PEO/IUO BD CD4 System and PEO/IUO CD4/Hb Cartridge
        ii. CD4 predicate= FACSCalibur/ Tritest

4. Reference Intervals: Single site prospective study (1OUS)

    a. 120 capillary and 120 venous blood specimens form hematological normal subjects that provide valid results
    b. 2 number of staff members (testing and administrative functions)
    c. IRB with approved informed consent and Study Agreement
    d. Conducted under Good Clinical Practices and GLP
    e. BD will monitor sites and data
    f. Estimated start date: August, 2013
    g. Systems
        i. PEO/IUO BD CD4 System and PEO/IUO CD4/Hb Cartridge

    h.

1.4 Proposed Intended Use

The BD Point of Care CD4 is an automated system for *in vitro* diagnostic use in performing the direct enumeration of CD4 absolute count, CD4 percentage of lymphocytes, and Hb concentration in human whole blood.

2.0 OBJECTIVES AND DESIGN

2.1 Primary Objective(s)

- The objective in phase I (Performance Evaluation procedure) is to -determine the method bias between the “For Performance Evaluation Only” (PEO)/ “Investigational Use Only” (IUO) labeled BD FACSPresto System versus the predicate system (IVD Tritest™ CD3/CD4/CD45 reagent with Trucount™ tubes and BD Multiset™ software on the FACSCalibur™ flow cytometer) in the determination of Abs CD4 and %CD4, using venous blood in de-identified specimens.

- The objective in phase II will be to determine the method bias between the PEO/IUO BD PFACSPresto System and PEO/IUO BD Point of Care CD4/Hb Cartridge versus the IVD

FACSCount system (BD FACSCount System with FACSCount CD4 Reagents and software), FACSCalibur™ flow cytometer and HemoCue 301 in the determination of Absolute CD4, %CD4 and Hb using de-identified patient specimens.

- Three objectives are planned to cover phase III:
    1. The first objective covering accuracy is to determine the method bias between the PEO/IUO BD Point of Care CD4 System and PE**O**/IUO BD Point of Care CD4/Hb Cartridge versus the IVD FACSCount system (BD FACSCount System with FACSCount CD4 Reagents and software) and Sysmex in the determination of Abs CD4, %CD4 and Hb using de-identified patient specimens.
    2. The second objective covering stability is to generate product claim data for stability of whole blood specimens and stained samples using the investigational system (PE**O**/IUO BD FACSPresto System and BD CD4/%CD4/Hb cartridge) in prospectively collected venous and capillary blood patient specimens at single external study sites.
    3. The third objective covering reference interval is to generate product claim data for normal reference intervals from normal whole blood specimens and stained samples using the investigational system (PEO/IUO BD FACSPresto System and BD CD4/%CD4/Hb cartridge) in prospectively collected venous and capillary blood from hematological normal subjects at single site.

2.2 Secondary Objective(s)

1. The secondary objective of this study, enshrined within phase III are the following: Determine method bias between the PEO/IUO labeled BD FACSPresto System versus the predicate system in the determination of Abs CD4 and %CD4, using capillary blood in de-identified specimens at three or more external study sites.
2. Determine the method bias between the PEO/IUO BD FACSPresto System versus the predicate system for Hb, Sysmex (KX-21) hematology analyzer, in the determination of Hb concentration using venous blood and capillary blood in de-identified specimens.
3. Evaluate the customer’s ease of use of the IUO PEO/IUO BD FACSPresto System following brief training.

2.3 Overall Study Design / Outcome Measures

The design applies to Phase III, Regulatory CE-IVD and US FDA 510K Submission for market clearance (BD Protocol number: CAS-PCACC1, Version 2.0).

This is a prospective, multisite study to determine the relative bias between the PEO/IUO BD FACSPresto System versus the IVD FACSCalibur™ with BD Tritest™ CD3/CD4/CD45 and Hb Sysmex methods in their determination of Abs CD4, %CD4 and Hb concentration. The design and proposed analysis of this evaluation are based on the CLSI EP9-A2 guideline on method comparison and bias estimation using patient samples.[9]

This study is planned to include three or more sites, three of which are external to BD that will enroll prospectively procured peripheral venous blood and capillary blood specimens from subjects willing to provide written informed consent and attending for routine flow cytometry lymphocyte subset evaluation (i.e., CD4 counting) and de-identified from the subject’s health personal information. The fourth site, the

BD MedLab will enroll only prospectively procured HIV de-identified venous blood specimens from HIV infected subjects for Hb determination.

The target number for enrolled specimens providing valid testing results (i.e. meeting protocol requirements) is a minimum total of 400 specimens from children s (0-12 yrs) and adolescent/adult subjects aged ≥ 13 years. Specimens must also fulfill age and CD4 andHb binning requirements as described in Section 4.2.2.

2.4 Expected Study Duration

I. **Feasibility Phase:** The expected study duration from start of enrollment to the completion of feasibility study testing is anticipated to span approximately three weeks.

II. **External Customer Evaluation:** The expected study duration of the Customer Evaluation from start of enrollment to the completion of study testing is anticipated to last approximately three weeks.

III. Regulatory for CE-IVD and US FDA 510K Submission for market clearance: The three activities in this phase (Accuracy, Reference interval and Stability evaluations) are expected to overlap within study duration of 16 weeks from the enrollment to completion of testing of specimen.

Proposed Timeline

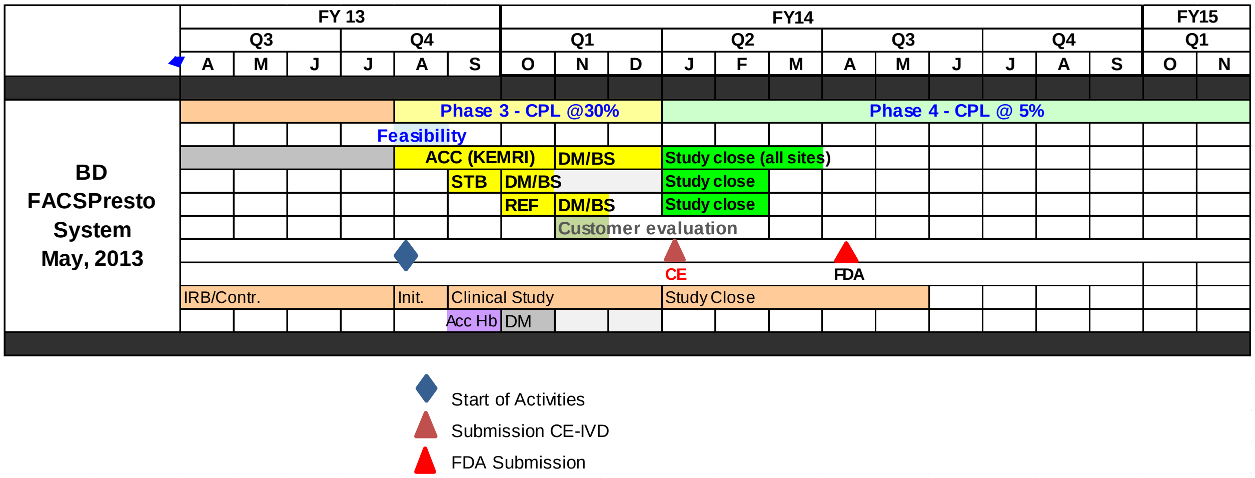

2.5 Study Device(s)

2.5.1 Investigational Device(s)

The investigational device for this study is the PEO/IUO labeled BD FACESPresto System, that consist of the instrument with integrated software (BD FACSPresto Instrument) and PEO/IUO BD CD4,%CD4/Hb cartridges with intergrated quality controls that will provide the absolute count of CD4+ T lymphocyte cells/mL of blood (Abs CD4), the percentage of CD4-positive T lymphocytes of total lymphocytes (%CD4) and concentration of Hb.

2.5.2 Predicate Devices

The predicate devices to be used in this Performance Evaluation procedure will involve currently marketed devices used for CD4Abs counts, %CD4, and Hb concentration measurements.

- For CD4Abs and %CD4 measurements, the BD FACSCalibur flow cytometer, BD Multiset software version 1.1 or later and BD Tritest™ CD3/CD4/CD45 reagent with Trucount™ tubes will be used to test peripheral whole blood specimens (WB).
- The predicate device to measure hemoglobin concentration is the Sysmex hematology analyzer (KX-21).

3.0 STUDY POPULATION

3.1 Description of Study Site

Since 1979 Kenya Medical Research Institute (KEMRI) has collaborated with the Centers for Disease Control and Prevention (CDC) to operate a field research station in western Kenya, near the city of Kisumu in Nyanza Province. The KEMRI/CDC Research and Public Health Collaboration was originally established as a malaria research program and has since expanded, experiencing rapid growth in terms of the number and scope of research and program activities, as well as human resources and physical infrastructure. Today, the primary research activities include malaria, HIV, TB, schistosomiasis, and emerging infections. The KEMRI/CDC Research and Public Health Collaboration is housed in three state-of -the art facilities. Administrative offices and laboratories are located at the main KEMRI/CDC field research station at Kisian (15 minutes from Kisumu). There are currently two Clinical Research Centers (CRCs) associated with KEMRI/CDC. One CRC is located on the grounds of the **Jaramogi Oginga Odinga Teaching & Referral Hospital (JOOTRH** formerly New Nyanza Provincial Hospital) for processing - PGH) in downtown Kisumu; which has high influx of patients, approximately 21,000 patients on care and 10,000 on treatment for HIV/AIDS. Additionally, KEMRI/CDC HIV-Research laboratory is located less than three kilometers from two other major health facilities namely, Kisumu District hospital which has about 5000 patients on care and about 5000 on treatment and Lumumba health center which has about 9000 patients on care and about 7000 on treatment. The laboratory has and is still supporting clinical trials like HPTN-052.[5,6]

The laboratory has also developed population-based biochemistry, immunological and hematological reference values for adolescents and adults in western Kenya that have been utilized by recent clinical studies in this region[11].

3. 2 Specimen Requirements

This study will be conducted at three or more reference laboratories specializing in flow cytometric immunophenotyping (See Appendix 1: Study sites). The study requires testing of prospectively procured

venous blood and capillary blood specimens from HIV/AIDS patients **for phase I, II and III (objective 1&2) and any persons/patients** willing to participate in the study and provide written informed consent **irrespective of their HIV status for phase III (objective 3).**

3.3 Inclusion Criteria

The following criteria must be met for the subject and whole blood specimens to be enrolled into this study:

Subject:

- **Phase I, II and Phase III objective 1&2:** Has been infected with HIV and willing to provide written informed consent to draw venous and capillary blood.
- **Phase III objective 3: Any persons irrespective of their HIV status who are willing to provide written informed consent to draw venous and capillary blood.**
- If minor, parent(s) are willing to provide informed consent and if child aged ≥ 13 yrs and above, willing to provide assent (in addition to parental/guardian consent) to draw blood.
- Agrees to grant access to her/his CD4 testing medical records **if available** for pre-screening.
- Agrees to disclose age and gender
- Agrees to disclose **any** co-morbid conditions information: malaria, tuberculosis, anemia, sickle cell anemia, infectious diseases, thalassemia and other current medical condition
- Agrees to disclose current medications.

Specimen:

- Patient has provided written informed consent for specimen draw
- Venous blood collected in a blood collection tube with EDTA anticoagulant and stored at room temperature (20-25°C) and according to the collection tube manufacturer's guidelines until enrollment.
- Venous blood drawn within an adequate time to perform post-enrollment staining within 24 hours.
- Venous blood of acceptable quality for flow cytometry testing (e.g., no hemolysis or clots and acceptable pre-analytical handling).
- Venous blood of sufficient volume: > 1mL for Sample Preparation.
- Capillary blood meets requirements for specimen donation as required.

Capillary blood applied onto the PEO/IUO BD CD4/%CD4/Hb cartridge. 3.4 Exclusion Criteria

ANY of the following is regarded as a criterion for excluding a specimen from the study:

- Unwillingness to provide written informed consent.
- Unwillingness to disclose medical information regarding previous CD4 testing results.
- Unwillingness to disclose medical information regarding co-morbid conditions.
- Enrolled specimens may be subsequently excluded from the study if found to be unsuitable for testing; for example, if visual inspection prior to acquisition shows clotting or hemolysis.

3.5 Sample Size Determination

The total sample size including the Phases I, II, and III is estimated a minimum of 450 pediatric, adolescent and adult patient venous and capillary WB specimens, from the study site that provide valid results (i.e. meeting enrollment and protocol testing criteria) will be required for the three phases of the study. The selection of samples for this study will follow a convenience sampling method, and will

include representatives at both extremes of the 95% CI for CD4 and Hb parameters. The projected number of samples, as detailed below for each phase of the study, is in accordance with and exceeds the revised CLSI guidelines for method comparison[9] so as to increase the power of subsequent analyses.

I. **Feasibility Phase:** A minimum of **2**00 venous and **2**00 capillary specimens.

II. **External Customer Evaluation:** A minimum of 50 venous and 50 capillary blood specimens.

III. **Regulatory submission for CE-IVD and US FDA 510K Submission for market clearance Evaluation:** A minimum of **400** venous and **400** capillary blood specimens **from three or more sites**.

4.0 ACCURACY STUDY GENERAL METHODS AND PROCEDURES

These procedures are replicable in other phases of the study. Details for every phase are already provided in this protocol.

4.1 Daily Study Activities

Daily study activities include the following:

- Subject and specimen enrollment
- Instrument set-up and Process Controls
- Sample Preparation and Testing, and
- Data review, Compilation and daily update to BD
- Weekly submission of the data to BD

4.1.1 Subject Enrollment

On each day of study testing, the Study site staff will inquire to the HIV/AIDS patients attending for flow cytometry lymphocyte subset evaluation (CD4 testing) routine testing if they would be willing to participate in the study and donate blood. The subjects willing to participate in the study will be evaluated for inclusion. If all inclusion criteria are met and none of the exclusion criteria are positive the subject will undergo the informed consent process and provide written informed consent prior to any study-related blood draw. Designated site staff (i.e. study coordinator) will pre-screen the subject's medical records to compile and identify the previous CD4 absolute counts and percentage CD4 results for CD4/Hb bin placement and whether may satisfy the study age and CD4/Hb bin requirements (see sections 5.2.1 and 5.2.2).

Then, subject's venous blood will be drawn according to the facility phlebotomy procedures. Capillary blood will be collected following procedure shown in Appendix 2.

All specimens enrolled in the study will be de-identified from the patient's personal health information by the assignment of a study-specific specimen ID prior to study testing. Guidelines for target enrollment populations are detailed in Section 5.2.1 and 5.2.2.

During the enrollment process, specimen information will be documented on Case Report Forms (CRFs). In addition to documenting that inclusion criteria have been met, the following patient demographic data will be captured:

- Patient's age (for bin assignment)
- Patient's gender
- Specimen collection date and time
- The hand (left or right) used for capillary blood collection
- Relevant co-morbid conditions: Anemia, Sickle Cell Anemia, Tuberculosis, Malaria, Thalassemia, Infections, other medical conditions
- Relevant medications: ART, antimalaria, antituberculosis, other

The information that is brought forward from diagnostic specimen identification during study enrollment will be considered to be source documentation once transferred to the CRF, and there will be no re-linking performed to verify the accuracy of this data against diagnostic records.

Figure 1: Venous and Capillary Blood Testing

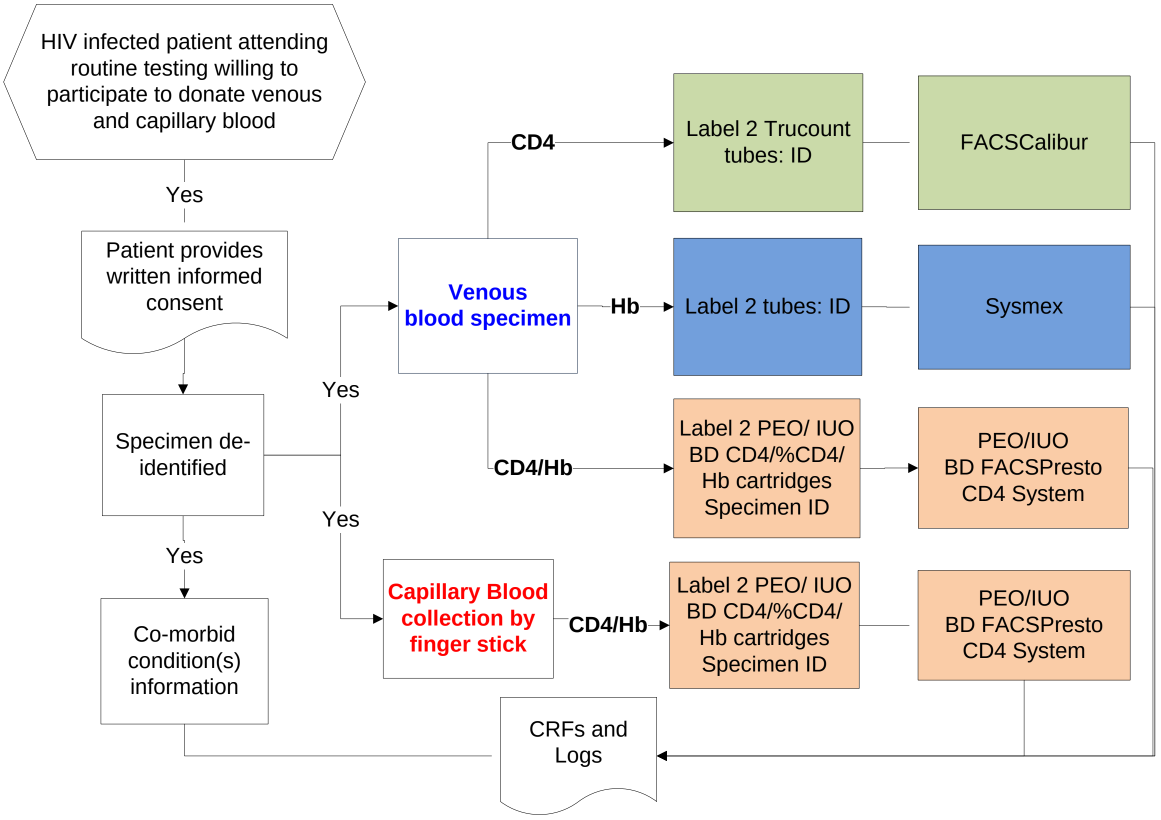

4.1.2 Venous Blood Specimen

Venous specimen

Venous blood specimen enrollment will involve:

- Patient provided written informed consent.
- Evaluation of specimens for enrollment based on inclusion/exclusion criteria.
- Collecting demographic information.
- De-linking the existing specimen ID by assigning a study-specific specimen ID to the aliquot/specimen tube and all applicable study documentation
- Completion of Case Report Form(s) (CRFs) and any additionally required logs.

Phlebotomy will be performed by an experienced clinical staff, to draw about 2 ml of blood for the study procedures.

4.1.3 Capillary Blood Collection

Capillary blood collection will be done according to the site approved standard procedures and CLSI H04-A6[9] and the method of capillary blood collection depicted on appendix 9 of this protocol Overview

- Ensure subject has willingly provided written informed consent
- Prepare supplies and subject for capillary blood collection
- Apply the methods described in Appendix 2
- Transfer bood to the PEO/IUO BD CD4/%CD4/Hb cartridges, by positioning the cartridge directly beneath the puncture site.
- Ensure blood flows through the channel
- Lock the PEO/IUO BD cartridge cap.

Ensure subject's safety by monitoring hemostasis before releasing the subject.

4.1.4 Enrollment by Subjects Age

A minimum of 400 valid AbsCD4 results are required to be collected in four age groups, the anticipated number patients enrolled by age is as follows:

Group	Patient Age	Anticipated
Children	**0-12 years old**	**80**
Young	13-25 years old	**100**
Mid	26-50 years old	**160**
Older	> 51 years old	**60**

4.1.5 Enrollment by CD4 Count

Enrollment by AbsCD4 and %CD4 binning will be done using the first replicate of the predicate results. If diagnostic CD4 counts are available at the time of specimen enrollment, site should enroll an approximately equal number of specimens in each CD4+ absolute count, %CD4 bins as shown in the following tables (specimens can be from any age):

Abs CD4 Bins for Accuracy

AbsCD4	Bins (cells/ μL)	Total # Desired	Minimum
	1) 50 ≤ CD4 < 250	100	80
	2) 250 ≤ CD4 < 500	100	80
	3) 500 ≤ CD4 < 1000	100	80
	4) 1000 ≤ CD4 ≤ 5000	100	80
**%CD4**	**Bins (%CD4)**	**Total # Desired**	**min**
	1) 5 ≤ CD4% < 20%	133	110
	2) 20 ≤ CD4% < 35%	134	110
	3) 35 ≤ CD4% ≤ 65%	133	110

4.1.6 Enrollment by Hb (only venous blood)

For Hb binning assignment will be done using the first replicate from the predicate method (Sysmex KX-21), Hb enrollment will follow the binning shown below.

Hb Bins for Accuracy

Bins (g/dL)	Anticipated total	Max-min
1) 2≤ Hb < 9	140	90
2) 9 ≤ Hb <13	180	100
3) 13 ≤ Hb <20	80	60

Clinical decision points for Hb (only capillary blood)

Bins (g/dL)	Anticipated total	Minimum
4.5 + 1*	80	40
10.5 + 1*	80	40
17 + 1*	80	40

*FIO: For information only

To complete bin enrollment specifically for the Hb bins, up to 15% of the patient samples may bemanipulated by diluting or spinning off plasma and then tested.

Only the BD MedLab will manipulate the venous blood samples for the Hb bins. If the minimum number of

specimens is not enrolled at each clinical decision point for Hb; the sites will be asked to use Hb controls to complete enrollment.

4.1.7 Instrument Setup/Instrument QC

4.1.7.1 PEO/IUO Method for CD4 – BD FACSPresto

On each day of study testing, turn on the BD FACSPresto, the system will automatically start the QC/setup. Verify all parameters achieve "pass' status, if necessary repeat again.

Passing results must be obtained prior to running any process controls or any patient specimens. If the BD FACSPresto instrument still deos not achieve "pass' status, contact BD.

- Process controls for the Investigational system
    - For AbsCD4 and %CD4, commercially available process controls supplied by the sponsor (i.e. BD Multicheck low/normal or Lymphosure low/normal or CDChex Plus/Chex CD4 Low, etc.) will be applied on BD CD4/%CD4/ Hb cartridges per process control.
    - For Hb, Eurotrol 301 (Low, Normal and High) instructions for use (IFU) will be applied on the BD CD4/%CD4/Hb cartridges for each control.
    - Incubate cartridges for a minimum of 18 min to 2 hrs on the incubation tray.
    - Load sample cartridges and start the run within 2 hrs of applying the control.

  Verified results (results i.e., AbsCD4, %CD4, AbsCD3, %CD3 and Hb concentration) to ensure they fall within the manufacturer's lot-specific ranges.

4.1.7.2 Predicate Method for CD4 – BD FACSCalibur™

The following are required:

- BD FACSCalibur™ flow cytometer
- BD Calibrite™ beads
- BD FACSComp™ software

On each day of study testing, predicate BD FACSCalibur system set-up must be performed and passing results obtained prior to running any process controls or enrolled patient specimens. This setup is performed by running BD Calibrite™ beads using the BD FACSComp™ software (Lyse/No-Wash assay selection) as follows:

- Prepare BD Calibrite beads per instructions in package insert. Run the two tubes with calibrate beads using Lyse/No-Wash assay selection in BD FACSComp™ software per instructions in the FACSComp User guide.
- Obtain a "pass" result on the FACSComp™ report and generate a hard copy print-out to document that the instrument setup is acceptable.
- Check to ensure that the threshold has been adjusted to minimize debris according to instructions in the BD Tritest™ CD3/CD4/CD45 Reagent Package Insert.

The flowchart illustrated on Figure 2 summarizes the key steps for daily process controls for CD4 Predicate Method (refer to the appropriate package inserts for further details).

Process controls for the predicate system (Tritest CD3/CD4/CD45 reagent with Trucount tubes and Multiset software on the FACSCalibur flow cytometer equipped with the BD FACS Loader).

- The process controls provided by Sponsor (normal and low) must be prepared daily and acquired.
- Verify results (i.e., AbsCD4, %CD4, AbsCD3, and %CD3) to ensure they fall within the manufacturer's lot-specific ranges.

4.1.7.3 Predicate Method for Hb – Sysmex (KX – 21)

The following are required:

- Sysmex (KX-21)
- Sysmex process controls provided by the sponsor (low, normal and high)
- 1.5 mL microcentrifuge tubes

On each day of study testing, turn on the instrument, wait for instrument self check.

- Select Quality Control Analysis and "QC Analyte"
- Place the uncapped control sample under the aspiration probe and press "Start"
- Once analysis is completed review the Hb and verify values, then accept the print.

Figure 2: Daily CD4 Predicate Method Process Controls

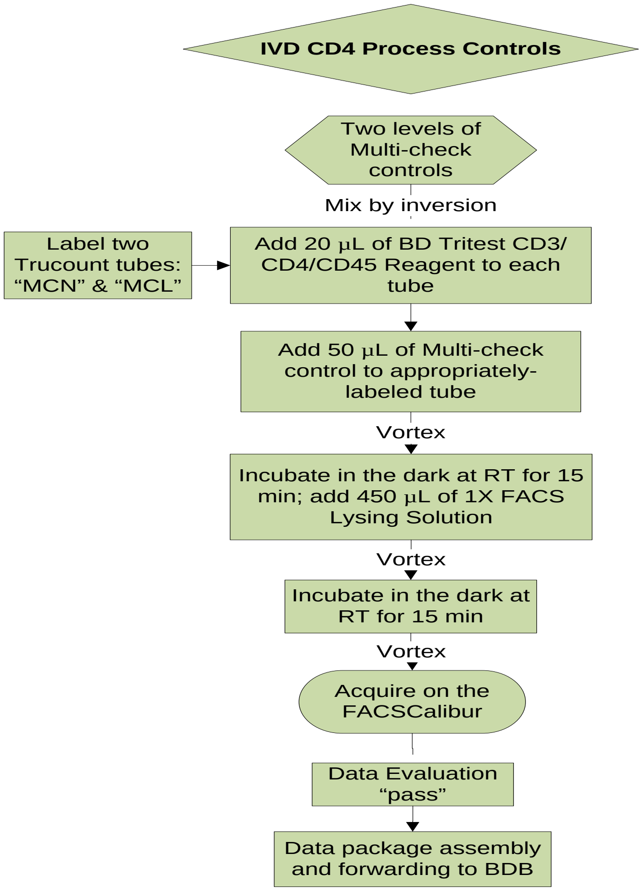

Figure 3: Sysmex Quality Control and Process Controls

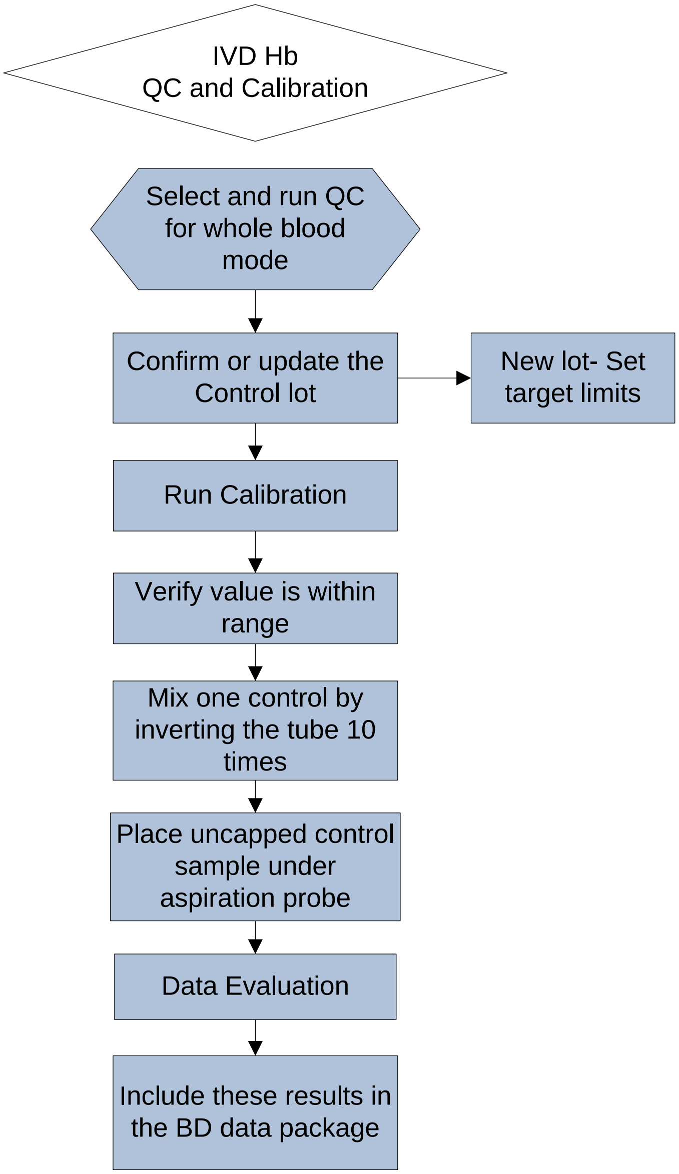

.

4.2 Enrolled Specimen Testing

4.2.1 BD FACSPresto Method: Sample Preparation

1. Label four PEO/IUO BD CD4/%CD4/Hb cartridges according to an ID scheme provided by BD.
2. Prepare samples for the PEO/IUO BD FACSPresto System as follows (see PEO/IUO BD CD4/%CD4/Hb cartridge assay IFU for additional information):
    - Open the cartridge, write patient's ID in provided white space
    - Enter sample information on the touchscreen (up to 10 samples)

Venous Blood

- Label two “PEO/IUO” BD CD4/%CD4/Hb cartridges according to an ID scheme provided by BD.

- Invert the specimen ten times, then use a transfer pipette to apply venous blood onto the PEO/IUO BD CD4/%CD4/Hb cartridge
- Incubate the cartridge for a minimum of 18 min to 2 hours on the incubation tray.
- Load sample cartridges and start the run.

Capillary Blood
- Label two “PEO/IUO” BD CD4/%CD4/Hb cartridges according to an ID scheme provided by BD.
- Prepare patient for capillary blood collection.
- Clean the finger with an alcohol swab, allow alcohol to dry
- Use a new lancet, applying it to the clean surface of the finger
- Puncture the skin and remove the first blood drop, then apply the 2nd drop of blood to the open port of the cartridge
- Incubate the cartridge for a minimum of 18 min to 2 hours on the incubation tray.
- Load sample cartridges and start the run

.

4.2.2 Predicate FACSCalibur™ Method
1. Label two BD Trucount™ tubes according to an ID scheme provided to the site by BD..
2. Prepare samples for the predicate system as follows (see the BD Tritest™ CD3/CD4/CD45 Reagent package insert for additional information):
    - Pipette 20 µL of BD Tritest™ CD3/CD4/CD45 Reagent into each labeled Trucount™ tube, being careful not to touch the Trucount™ bead pellet (according to the package insert).
    - Thoroughly mix the WB specimen by gentle inversion and pipette 50 µL (using reverse pipetting) into each Trucount™ tube.
    - Cap each tube and vortex to mix.
    - Incubate the tubes in the dark at room temperature (i.e., 20 to 25 ºC) for 15 minutes.
    - Pipette 450 µL 1X BD FACS Lysing Solution into each tube.
    - Cap each tube and vortex to mix.
    - Incubate in the dark at 20 to 25 ºC for 15 minutes.
3. Acquire the BD Trucount™ tubes on the FACSCalibur™ immediately (vortex prior to acquisition) or store in the dark at 20 to 25 ºC. Acquire within 24 hours of blood draw..
4. Review the dot plots and verify that at least twenty five hundred (2,500) lymph events have been collected, to consider the predicate data “evaluable”.

NOTES:
- *Re-analysis of a sample is allowed. A sample can be acquired two times.*
- *BD MedLab will be the sole site that will use venous blood manipulated samples to test Hb concentration*

4.2.3 Predicate Hb Method
- Label two microcentrifuge tubes according to the ID scheme provided to the site by BD before transferring the blood
- Select whole blood mode

- Mix the specimen by inverting the tube 10 times
- Transfer 100µL into a minicentrifuge tube
- Acquire sample
- For the next sample, mix specimen by inverting the tube 10 times, transfer 100mL into a microcentrifuge and acquire the sample.
- Print sample results

4.2.4 Enrolled Sample Acquisition and Analysis

4.2.4.1 BD FACSPresto Method

- Enter the specimen ID and date information using the touch screen
    - Once incubation is finished, load the investigational cartridge into the instrument.
    - The instrument will automatically read and calculate Abs CD4, %CD4 and Hb concentration.
    - Print sample results. Review results, if there is quality control message , refer to IFU for troubleshooting
    - Repeat steps with second replicate cartridge

4.2.4.2 Predicate FACSCalibur™ Method

- Acquire the BD Trucount™ tubes on the FACSCalibur™ immediately (vortex prior to acquisition) or store in the dark at 20 to 25 ºC.
- Acquire within the timeframes specified in the BD Tritest™ reagent package insert.
- Print and review results

4.2.4.3 Predicate Hb Method

- Place the well mixed and uncapped specimen under the aspiration probe and analyze
- When the analysis is complete, review and print results
    - Write results onto the corresponding CRF
    - Repeat procedure with a second tube

4.2.4.4All Methods

Review the acquisition data for acceptability (see Section 7.3 for further details) and, if no reacquisition is required, discard the IUO vials and IVD tubes.

Figure 4: Enrolled Specimen Testing

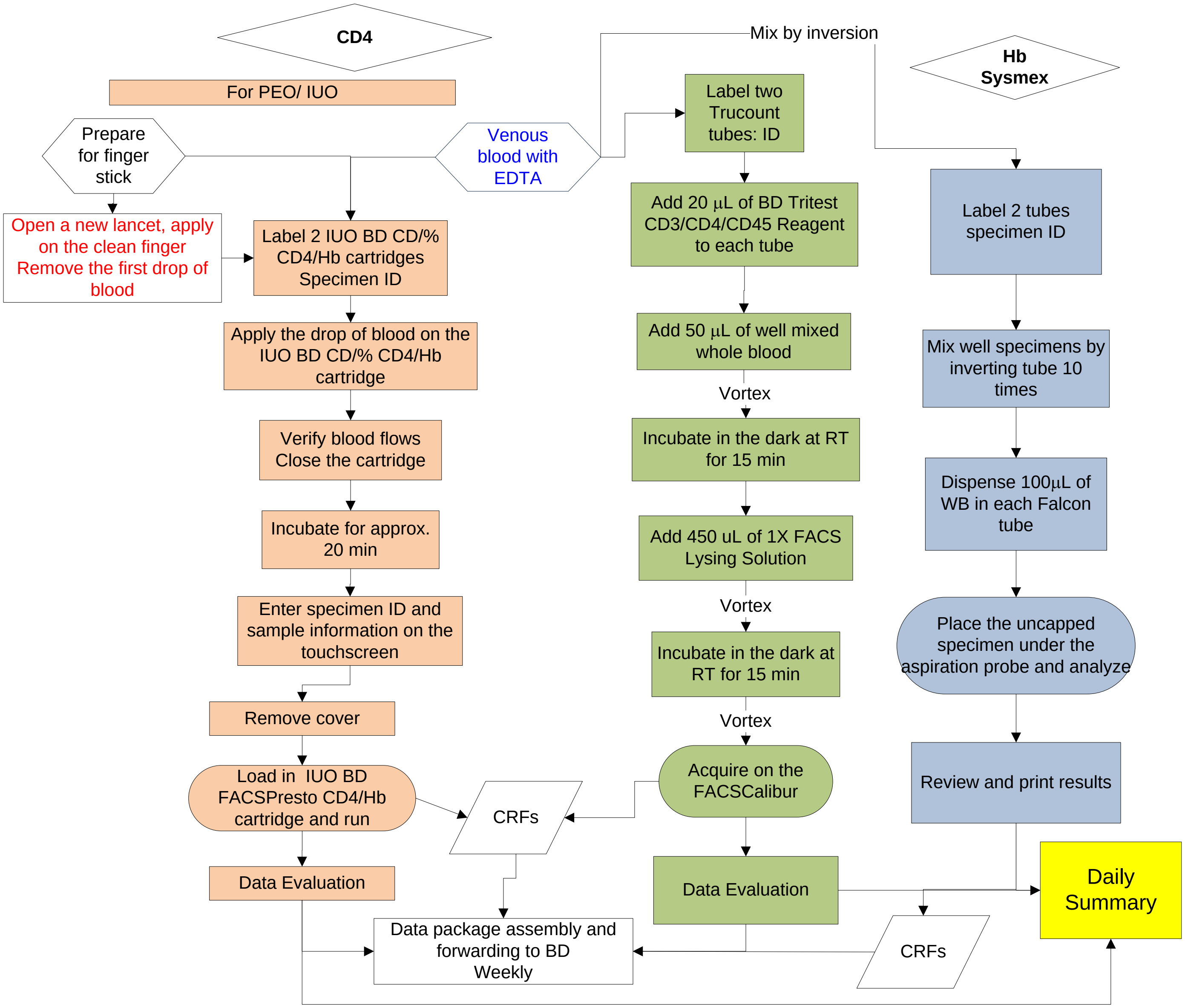
CD4
For PEO/ IUO
Prepare for finger stick
Open a new lancet, apply on the clean finger Remove the first drop of blood
Label 2 IUO BD CD/% CD4/Hb cartridges Specimen ID
Apply the drop of blood on the IUO BD CD/% CD4/Hb cartridge
Verify blood flows Close the cartridge
Incubate for approx. 20 min
Enter specimen ID and sample information on the touchscreen
Remove cover
Load in IUO BD FACSPresto CD4/Hb cartridge and run
Data Evaluation
CRFs
Data package assembly and forwarding to BD Weekly
Venous blood with EDTA
Mix by inversion
Label two Trucount tubes: ID
Add 20 µL of BD Tritest CD3/CD4/CD45 Reagent to each tube
Add 50 µL of well mixed whole blood
Vortex
Incubate in the dark at RT for 15 min
Add 450 uL of 1X FACS Lysing Solution
Vortex
Incubate in the dark at RT for 15 min
Vortex
Acquire on the FACSCalibur
Data Evaluation
CRFs
Hb Sysmex
Label 2 tubes specimen ID
Mix well specimens by inverting tube 10 times
Dispense 100µL of WB in each Falcon tube
Place the uncapped specimen under the aspiration probe and analyze
Review and print results
Daily Summary

4.2.5 Customer's Ease of Use

Ease of Use the PEO/IUO BD FACSPresto System will be evaluated in a minimum of 2 operators per site after they have completed Proficiency Training and before the start of enrollment. Ease of Use Assessment will be done using a survey addressing specific aspects of the functionality of the PEO/IUO BD FACSPresto System and BD CD4/%CD4/Hb cartridge. The operators will complete and return the Eases of Use survey to BD.

Ease of use survey will consist of questions addressing workflow and functionality of the PEO/IUO BD FACSPresto System. Each question will have 6 possible answers with a numeric value assigned for analysis. Example of the answers as the following:

- Extremely easy/satisfied, numeric value =5
- Somewhat easy/satisfy, numeric value =4
- Neutral, numeric value =3
- Slightly difficult/ dissatisfied, numeric value =2
- Very difficult/ dissatisfied, numeric value =1
- Not applicable (NA), numeric value =0

5.0 DISCONTINUATION OF SPECIMEN TESTING

Post-enrollment, instances may occur that will require the discontinuation of the WB specimen testing such as:

- If further visual inspection of an enrolled specimen reveals clotting and/or hemolysis that initially were not apparent, this specimen would be unsuitable for study testing or, if results have been generated, these results would not be included in analysis.
- If an enrolled specimen (of sufficient quality) is broken/spilled, this "quality not sufficient (QNS)" specimen would require discontinuation of study testing
- If an enrolled specimen (with adequate time post-draw to enroll for study testing) cannot be stained within 24 hrs due to unanticipated testing difficulties, this specimen would require discontinuation of study testing and document reason for withdrawal.

6.0 PRODUCT ERRORS/ DEFECTS/ FAILURES

Errors, defects or unanticipated failures observed for the investigation study product(s) will be documented using the study error forms and communicated immediately to the Study Monitor.

These events will be documented and escalated in accordance with BD internal procedures.

7.0 DATA COLLECTION AND MANAGEMENT

7.1 Data Collection and Source Documents

Study data will be collected in the form of standardized case report forms (CRFs)/ electronic CRFs (eCRF) and other data collection forms that will be provided for use at all investigational sites. Instrument data will also be collected in the form of instrument records (electronic files or printouts) such as from instrument set-up and sample acquisition results in different file formats (CSV, EXP, PDF, FCS, daily summary in Excel spreadsheet, etc.) are considered source documents.

Source documents may also include supporting medical records and informed consents, diagnostic test results from other laboratory equipment, or even CRFs, when the original collection of data is done directly on the case report form. Investigators will maintain all source documents associated with the protocol, including laboratory results and instrument reports. All hard copy and electronic data will be secured to ensure confidentiality. The source documents will be used for study monitoring and data management review to verify study compliance.

In verifying data integrity, the monitor will ensure that the data in the CRFs are consistent with protocol requirements. It is not necessary for every data entry point captured on a CRF to have a matching source, however, where data appears in both, they must agree.

7.2 Submission of Data Packages to Sponsor

Investigators are responsible for the collection, review, and timely submission of all required study data to BD for processing, including instrument electronic files, which will be acquired and saved in specified study archives.

It is anticipated that each site will provide daily update of the acquired data according to the process that the sponsor will define during site initiation and training.

All study data that include paper and electronic documentation of the study, will be submitted to BD in a weekly package by courier. The PI or designee will ensure that the data and documentation are accurate, complete, and in compliance with applicable documentation requirements, prior to assembling and submitting data packages to BD.

BD will provide a checklist during site initiation and training that can be referenced during data package compilation and review. This checklist may contain, but not be limited to:

- List of the minimum required package items
- Key elements from the user documentation to be referenced for:
  - Data evaluation (e.g., flagged due to low lymphocyte events)
  - Predicate evaluation of plots (e.g., sub-optimal resolution of specified populations)
  - Instrument printouts

Quality assurance procedures should be followed to ensure that complete, accurate and timely data are submitted, that protocol requirements are followed, and that product failures, errors, adverse events and adverse device effects are detected, managed and reported as required, if applicable.

Upon completion of data package review, the site will forward this package to BD in regular shipments at intervals agreed upon by the CRA and PI (typically, once per week).

Figure 6: Study Data and Submission to BD

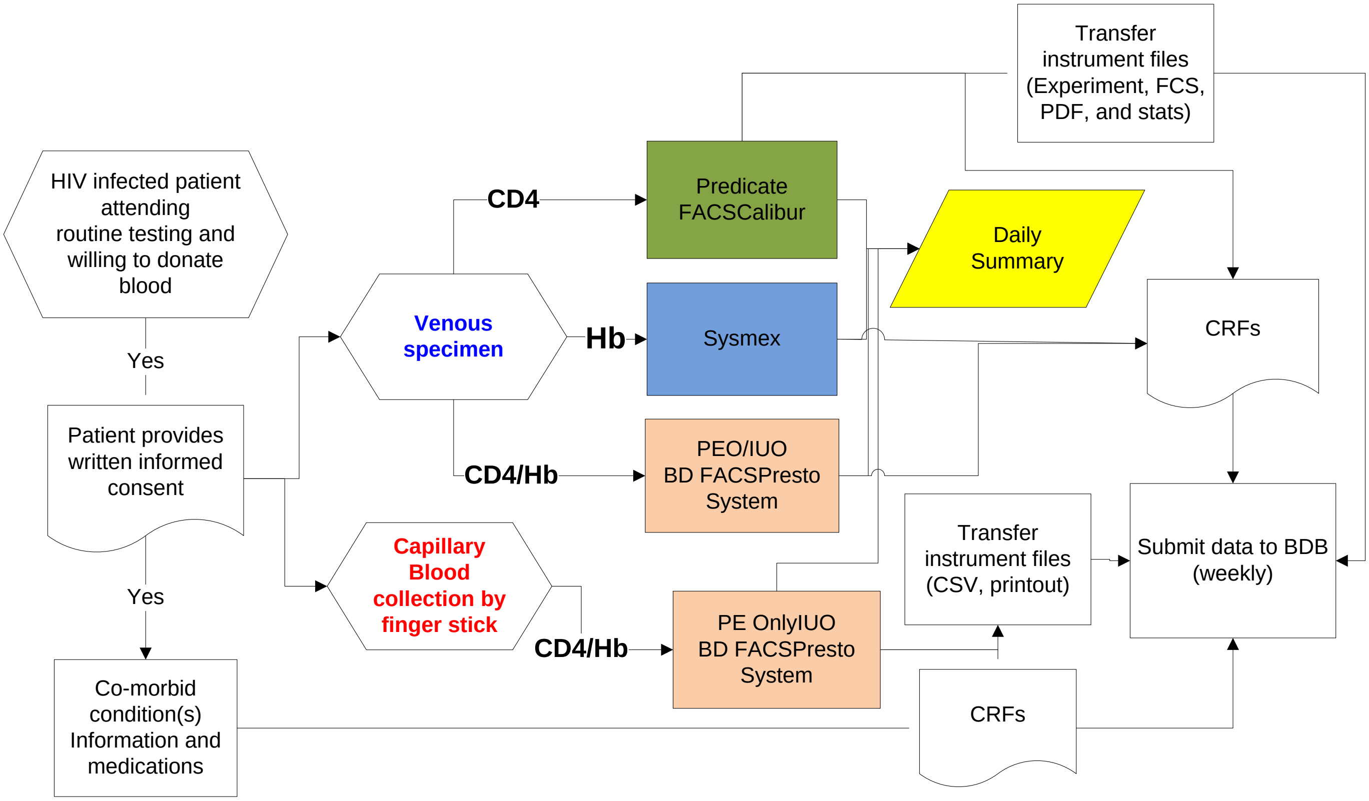

7.3 Data Evaluation for Inclusion/Exclusion from Analysis

After receipt of data packages from the sites, the BD CRA/study monitor or designee will review the contents to ensure that the data are accurate, complete, and in compliance with all applicable requirements.
In instances where data is found to be missing or incomplete, the CRA will contact the site to resolve data discrepancies using a data clarification form (DCF) for query resolution. Discrepancies may be addressed via calls to the investigational sites or during visits by site monitors. Based on the outcome of this review, the CRA may identify and provide a reason for specified data to be deemed not evaluable and possibly excluded from analysis (but not removed from the study database).

BD has targeted a minimal and maximum number of specimens to enroll based on age and CD4 bins (see section 4.2 Specimen Enrollment). As data is reviewed and all queries resolved, BD will track the number of evaluable specimens in each CD4 bin. When the evaluable number reaches the target number within each bin, BD will notify the sites so that specimens can preferentially be enrolled for other bins.

In the case of excess data points for any bin, data for any other specimens enrolled in that bin will be excluded from the final data analysis (but not from the study database). When selecting between multiple data points for inclusion in the final analysis, the data with an earlier enrollment date will be given preference.

7.4 Data Management and Storage

BD Data Management will be responsible for the entry, processing and maintaining of study data. All original CRFs will be sent to BD as part of the periodic submission package or be retrieved by the site monitor with a copy kept at the site. BD Data Management will process the data and will carry out edit checks, error checks and audits, if needed. The Investigator will be queried on issues concerning data completeness and consistency.
All above-mentioned tasks will be performed according to the Sponsor's relevant internal procedures; ensuring adherence to GCPs. Audits may be performed for quality assurance of data handling.

8.0 STATISTICAL METHODS

Statistical analyses will be conducted by the BD Biostatistics group.

8.1 Sample Size Determination

For the Accuracy procedure, a sample size of 400 pediatric, adolescent and adult patient WB specimens, from the study site that provide valid results (i.e. meeting enrollment and protocol testing criteria) will be required to determine the bias (expected difference) between the "PEO/IUO" system versus the predicate systems.

8.2 Eligibility, Exclusions, Missing Data, Interim Analysis

All specimens meeting the eligibility criteria and tested with the BD FACSPresto System will be analyzed for primary and secondary endpoints. It is anticipated that a minimum of 80% of the data will from matching venous and capillary blood specimens from the same subject for analysis. Management of dropouts and missing data will depend on their frequency and the particular outcome measure; any such adjustments will be described completely and documented. All valid data will be included in the analysis; if outliers are determined to exist, the monitor will investigate the identified outliers.

8.3 General Statistical Considerations

All statistical analyses will be performed using commercially available statistical software. Adequate source document verification and/or audit activities will be utilized to assure the validity of study conclusions. Analysis shall be performed based on methods described in statistical analysis plan or methods described in section of the Analysis Method of the protocol. All related and resulting reports, documents and data shall be produced and maintained in such a way as to ensure their control and the protection of subject privacy as far as is reasonably practicable. Data files and analytic reports will be archived according to requisite regulatory standards.

8.4 Study Endpoints / Outcome Measures

8.4.1 Primary Endpoint

The primary endpoint is the bias (expected difference) between the investigational system versus the predicate system measured as Abs CD4 and %CD4 in peripheral venous blood specimens from a minimum of 400specimens with valid results tested at three or more sites.

8.4.2 Secondary Endpoint

Secondary endpoints have been defined for the analysis of the AbsCD4, %CD4 and Hb results from investigational BD FACSPresto System and predicate methods using capillary and venous blood.

8.4.2.1 Evaluation of CD4 and Percentage of CD4 in capillary blood
Expected difference between the investigational system using capillary blood specimens versus the predicate system using venous blood specimens measured as AbsCD4 and %CD4.

8.4.2.2 Evaluation of Hb concentration in venous and capillary blood
Expected difference between the investigational system testing capillary blood specimens versus the predicate system using venous blood specimens measured as Hemoglobin concentration.

8.4.2.3 Enrolled Sample Acquisition and Analysis

The customers' response on the "ease of use" is a qualitative and quantitative assessment of how quickly, easy and safely an instrument-naïve operator should be able to use the PEO/IUO BD FACSPresto System , after 3 hrs or less training period and the "anxiety" factor as to fear of breaking something or losing large amount of patient data without noticing it..

8.5 Acceptance Criteria

8.5.1Primary Endpoint

- For Abs CD4 count, the 95% confidence interval for the slope estimated using Deming Regression shall be within 0.9 to 1.1 when regressing the investigational device to the predicate device. The R2 from the Deming regression shall be ≥0.9. The intercept estimated from the Deming Regression shall be within ±20 counts.

- For %CD4, the 95% confidence interval for the slope estimated using Deming Regression shall be within 0.9 to 1.1 when regressing the investigational device to the predicate device. The R2 from the Deming regression shall be ≥0.9. The intercept estimated from the Deming Regression shall be within ±3%.

8.5.2 Secondary Enpoints

CD4 in capillary blood

- For Abs CD4 count in capillary blood, the 95% confidence interval for the slope estimated using Deming Regression shall be within 0.90 to 1.10 when regressing the investigational device to the predicate device. The R2 from the Deming regression shall be ≥0.90. The intercept estimated from the Deming Regression shall be within ±20 counts.

- For %CD4 in capillary blood, the 95% confidence interval for the slope estimated using Deming Regression shall be within 0.90 to 1.10 when regressing the investigational device to the

predicate device. The R2 from the Deming regression shall be ≥0.90. The intercept estimated from the Deming Regression shall be within ±3%.

Hb in venous and capillary blood

- For Hb, the the 95% confidence interval for the slope estimated using Deming Regression shall be within0.9 to 1.1when regressing the investigational device to the predicate device. The R2 from the Deming regression shall be ≥0.9. The intercept estimated from the Deming Regression shall be within ±0.5 g/dL.
- For capillary blood Hb concentration, the mean difference (bias) for samples with Hb concentration of 10.5 ± 1 g/dL (clinical decision point) between the BD FACSPresto System result and the predicate system result shall be <7%, with > 95% confidence, with respect to specimens typical in the target markets.

OR

8.6 Methods to Reduce Bias

Blinding of diagnostic results from study personnel is facilitated by the de-linking process during study enrollment: study personnel who have access to subject information will not be involved in acquiring or analyzing specimens. The site personnel who will perform sample preparation and acquisition will have study-specific training to ensure consistent techniques to minimize bias.

8.7 Analysis Method for the Accuracy Evaluation Procedure

For the study data generated at the sites, the minimum number of WB specimens that provide valid study results (i.e., meeting all inclusion criteria and protocol requirements) is400 specimens.It is anticipated that the total number of enrolled specimens will exceed 400 to achieve the required number of evaluable specimen in each age, CD4 and Hb bins. The desired number for each CD4 and Hb bin is listed below:

8.7.1 Primary Endpoint CD4 Binning

Abs CD4 Bins for Accuracy

Bins (cells/mL)	Total # Desired	Minimum
1) 50 ≤ CD4 < 250	100	80
2) 250 ≤ CD4 < 500	100	80
3) 500 ≤ CD4 < 1000	100	80
4) 1000 ≤ CD4 ≤ 5000	100	80

%CD4 Bins for Accuracy

Bins (%CD4)	Total # Desired	Minimum
1) 5 ≤ CD4% <20%	133	110
2) 20 ≤ CD4% < 35%	134	110
3) 35 ≤ CD4% ≤ 65%	133	110

8.7.2 Secondary Endpoints CD4 and Hb Binning

The secondary endpoint binning for AbsCD4 and %CD4 in capillary blood will apply the same bins described for CD4 in venous blood in section 8.7.1.
The secondary endpoints will address Hb bias determination between the investigation and predicate methods using venous blood. Additional bias analysis will be required around the clinical decision point of 10.5 (9.5-11.5) for determination of Hb in capillary blood using investigational versus the predicate method using venous blood. Three bins have been defined for Hb.

Hb Bins for Accuracy

**Bins (g/dL)**	**Total # Desired**	**Minimum**
1) 2≤ Hb <10	140	90
2) 10≤ Hb <13	180	100
3) 13≤ Hb <20	80	60

Clinical Decision Points for HB (Only capillary blood)

Bins (g/dL)	Total # Desired	Minimum
**4.5 ± 1**	**60**	**40**
**10.5 ± 1**	**60**	**40**
**17**	**60**	**40**

For each specimen, the mean differences between investigational and predicate results (for both Abs CD4 and %CD4), will be calculated and pooled across all specimens to produce a mean difference. Estimates from the Deming regression will be used to evaluate the equivalency between the investigational and predicate systems, according to the acceptance criteria presented in Section 8.5

It is anticipated that the total number of enrolled specimens will exceed 400 in order to achieve the required number of evaluable specimens in each age and CD4 bin.

For each Hb specimen, the mean differences between investigational and predicate results will be calculated and pooled across all specimens to produce a mean difference. Bias analysis at the clinical decision point of 10.5±1 will be used to evaluate the equivalency between the investigational and predicate systems, according to the acceptance criteria presented in Section 8.4. Additional analysis at 4.5± and 17±1 may be included for information only
Note that when the number of evaluable specimens reaches the desired target within each CD4 bin, data for any remaining specimens enrolled in that bin may be excluded from final data

analysis (see Data Evaluation for eligibility from Analysis in section 8. 2for details). BD will track bin enrollment during the course of the study and will notify sites when a target for any bin has been reached, so that sites can preferentially enroll specimens for other bins.

Ease of Use data will be discrete and qualitative variables obtained from small number of individuals at the participating clinical sites in the study. Data will be summarize and presented in tables or graphs. Additional statistical analysis is anticipated to be limited. Note that a subset of the total Accuracy data (i.e., from the site enrolling the most specimens) may be analyzed separately for the purpose of updating the technical file for CE declaration.

8.8 Biostatistics Report

A statistical report will be prepared by the biostatistician after data analysis is complete.

9.0 STUDY MATERIALS AND SUPPLIES

9.1 Investigational Product Labeling

The following products will be provided bearing “PEO/IUO” label/labeling indicating that they may only be used for performance evaluation purposes only:
The “PEO/IUO” instrument, reagents, controls, and software will be provided bearing labels/labeling indicating “PEO/IUO”. Additional information regarding system operation and reagent composition will be contained in Performance Evaluation only instructions for use and package inserts that will be provided to the site prior to or during study initiation.

Additional information regarding product composition, handling, etc. will be contained in a “PEO /IUO” package insert.

9.2 Study Products Shipment and Receipt

BD will include appropriate shipping log(s) in all material shipments to the sites: Study Accountability Log(s) for PEO/IUO/IVD Instrument, Reagents, Software; and/or Miscellaneous Materials Shipment Log(s).

All “PEO/IUO” supplies for initial training will be labeled “Training only”. All “PEO/IUO” or IVD supplies for training and for the study will be verified upon delivery by site personnel and entered on the center's Study Accountability Logs. After receipt has been documented on Study Accountability logs, the log originals are retained by the site to allow for documentation of usage and final disposition of all investigational materials (i.e., used, unused and destroyed at the site, or unused and returned to BD).

9.3 Study Product Accountability

The Sponsor will provide the Investigator with investigational product upon receipt of all necessary study documentation (i.e. IRB/IEC approval letter and approved clinical trial agreement).

- During the investigation, the Investigator is responsible for ensuring that: Only specimens that qualify for study enrollment are run on the IUO system;
- At the testing laboratory, all investigational products and devices are maintained under controlled access storage, and the use of investigational products are strictly controlled;

- **For the duration of the study**, , any device or components that are removed from their packaging and not used are segregated from general use.

The Investigator is responsible for maintaining accurate accountability records for the use of investigational materials on the Study Accountability Logs. These records will be reviewed at all monitoring visits to ensure compliance, and will document:

- Receipt dates and quantity,
- Dates and quantity used, including identification of samples, and
- Amount used, discarded or returned.

Study Accountability Logs will be verified by the site monitor against the Shipping Logs.

9.4 Required Study Materials and Supplies

9.4.1 Instrumentation

If any of the following are not available at the site, required items will be provided by BD.

- BD FACSCalibur™with FACS Loader
- BD FACStation workstation
- BD Multiset Software version 1.1 or later
- BD FACStation Software version 4.2 or later (includes FACSComp™ and Worklist Manager)
- PEO/IUO BD FACSPresto Systme instrument and accessories
- PEO/IUO BD FACSPresto Software 1.0
- Sysmex analyzer (KX-21)

9.4.2 Reagents, and Controls

- BD Calibrite beads
- BD Tritest™ CD3/CD4/CD45 reagent
- BD Multicheck Process Controls (low or normal) or Lymphosure (Low and Normal) or CD-Chex Plus/Chex CD4 Low
- BD Trucount™ tubes
- BD FACS Lysing Solution
- BD FACSFlow for the FACSCalibur™
- PEO/IUO BD FACSPresto™ Reagent Kit (includes reagent PEO/IUO BD CD4/%CD4/Hb cartridges, lancets, alcohol swabs and transfer pipettes)
- Eurotrol 301 (Low, Normal and High)
- Sysmex reagents (SYSPK-30L; SYSSWH-200A)
- Sysmex L, N and H controls (three clinical sites; part number: SYS140-3004-0)

9.4.3 General Product Use and Study-Specific Documents

PEO/IUO BD FACSPresto System Instructions For Use
PEO/IUO BD FACSPresto System Safety and Limitations Gudie Review
PEO/IUO BD CD4/%CD4/Hb cartridge Instructions for Use

9.4.4 Ancillary Items

BD will provide the following:

- Lancets for capillary blood collection

- Transfer pipettes
- Cotton swabs
- Alcohol pads
- Band aids
- USB memory sticks for archival of IRB/EC results files
- CDs or USB memory sticks for archival of the instrument electronic files
- BD FACSPresto System Printer rolls
- Sysmex printer rolls
- 50 μL reverse pipette
- Portable Scanner

Sites should have access to the following:

- 450 μL pipette and pipette tips (to dispense 50 and 450 μL)
- Vortex mixer
- Lab timer

9.5 Biohazard Containment

As transmission of HIV and other blood-borne pathogens can occur through contact with blood, blood products and contaminated needles and needles, appropriate precautions will be used by all staff in the drawing of blood, and in shipping and handling of all specimens in this study in accordance to KEMRI/CDC recommendations. Post-exposure prophylaxis with antiretroviral medications will be available to all staff who experience a work-related needle stick injury or other blood or body fluid exposure. The employee, however, must agree to a baseline HIV test immediately following the injury and another one 3 months later. If the staff member is found to be HIV-infected at the time of the occupational exposure, post-exposure prophylaxis will not be provided, but rather they will be referred for appropriate HIV care and treatment.

9.6 Disposition

At the end of the study or upon request by the site monitor, any remaining investigational devices, used or unused, will be handled as follows:

Instruments

The BD PEO/IUO BD FACSPresto System: , at the site a staff member shall decontaminate as per laboratory procedures all BD-provided instruments or components prior to returning them to BD San Jose. PEO/IUO BD FACSPresto System will be returned in accordance with BD procedures for shipping and transportation and US customs. The monitor will coordinate shipping and return of the instrument to:

Imelda Omana-Zapata, PhD
Clinical Operations
BD Biosciences
2350 Qume Dr.
San Jose, CA 95131

Reagents

The study monitor will instruct whether all used "PE**O**/IUO" reagents, as well as those opened but not used, will be returned in accordance with BD procedures for shipping and transportation and US customs, or defaced and discarded at the site. No experimental devices or supplies will be used for any other purpose except for testing the specimens enrolled in this clinical study.

Unused reagents: At the end of the study or as instructed by the site monitor, the Investigator or his/her designate will return or deface and discard all remaining unused supplies to BD.

10.0 MONITORING

10.1 Medical Monitor

The medical monitor's responsibilities include but are not limited to the following:

- Review and approve clinical protocol and protocol modifications
- Review and approve CRFs and CRF modifications
- Review and approve protocol exceptions including eligibility and procedural deviations
- Review monitoring and study progress reports, and document and resolve issues as required
- Review adverse events, and document and resolve issues as required
- Support monitoring staff, and facilitate Investigator and study staff interactions as required
- Review and resolve any events or issues that may impact trial success, or patient, Investigator or study staff welfare
- Participate in the drafting and review of study analyses and reports regarding study outcomes

Medical monitors will be appropriately qualified by education and / or experience to perform all specified and necessary monitoring tasks. Medical monitoring will comply with the requirements of ISO 14155:2011, Clinical investigations of medical devices.

All study monitoring activities will be conducted in accordance to the Study Monitoring Plan and in accordance with the KEMRI/CDC policies and procedures.

10.2 Study Monitors

The study monitor's responsibilities are to ensure that:

- the study protocol is followed,
- timely and accurate data are submitted,
- inconsistent, incomplete or inaccurate data is corrected,
- the site facilities, staff and performance continue to be sufficient to ensure the validity of the scientific data, adherence to study requirements, and the protection of the health, safety and welfare of the study subjects.
- prompt reporting as required by the investigational plan and FDA regulations is achieved, especially regarding Unexpected Adverse Device Effects (21CFR part 812.50)

Study monitoring will comply with the requirements of GCP Guidelines and Section 9 of ISO 14155: 2011, Clinical investigations of medical devices. Study monitoring will be performed by qualified personnel, with appropriate education and / or experience to perform all specified and necessary monitoring tasks.

10.3 Monitoring Procedures

10.3.1 Case Report Forms (CRFs) Management

The site monitor will ensure that CRFs are completed for each included specimen in a timely fashion. Electronic files and instrument printouts will be monitored on-site and in-house.

Corrections and modifications made to data already written or completed on the CRF page should be legible, initialed and dated by approved personnel. The reasons for significant changes must be provided. Correction fluid or covering label must not be used.

10.3.2 Monitoring Procedure and Documentation

Monitoring visits will be conducted in accordance with Clinical Operations Standard Operating Procedures (SOPs), Desk Procedures (DPs), the study protocol, monitoring plan and applicable regulatory requirements. Monitoring visits to the study site will be scheduled periodically during the study, to ensure that Investigators and their staff understand and accept their defined responsibilities and that all aspects of the current approved protocol/amendment(s) are followed. Data collection forms, electronic files and study data will be reviewed for cross referencing for accuracy.

The Investigator/institution guarantees access to study documents and source documents related to the study by designated BD personnel and appropriate regulatory authorities. It is important that the Investigator and their relevant personnel are available during the monitoring visits and possible audits and that sufficient time is devoted to the process. The Site monitor will complete a report documenting completion of the each site visit.

All site visits will be recorded in a monitoring log maintained at each investigational site documenting the monitor and the date of the monitoring visit.

- **<u>Site Initiation Visit</u>**: Prior to a clinical investigation, the site monitor will ensure that the Investigator and the investigational site are prepared for the study. All the appropriate training materials and the user documentation for this clinical investigation will be provided to study personnel during the site initiation visit. Documentation of all necessary approvals, agreements, and training on the study and experimental treatment will be gathered during the visit.
- **<u>Interim Site Visit (s):</u>** A BD monitor will schedule and conduct Interim Monitoring Visits to monitor the clinical investigation throughout its active phase to ensure compliance with this protocol, IOU device accountability and data accuracy. The first Monitoring Visit during the active phase of the study will be scheduled according to the study monitoring plan to:
    - Review and cross review and cross reference informed consents for completion with specimen enrollment
    - Cross referencestudy data with source documents. BD Biostatistician will randomize all specimens and provide a randomization list, during site visit the monitor will cross reference study data with patient's source records. This requirement will be met when approximately 20% of the enrolled specimens are cross-referenced with the source documents.

- **<u>Site Close-out Visit</u>:** The BD monitor will assure that all site closeout activities are addressed when the study has been completed or terminated at the clinical site as indicated in the study monitoring plan.
- **<u>Compliance visits</u>**: In the event that a study site requires additional assistance to meet study protocol or regulatory requirements, monitoring visits will be conducted at more frequent intervals and / or with additional personnel to assure compliance.
- **<u>Other visits</u>**: the study site may be inspected by appropriate regulatory authorities; this includes visits by the Ministry of Health Representative(s).

10.4 Adverse Events

Anticipated adverse events for the study are:

- Mild discomfort
- Bruising
- Rare possible adverse events include fainting or infection at the site of the skin puncture

Adverse events will be reported using the appropriate form, email or fax to the KEMRI Ethics Committee and to the designate BD study monitor:

BD Biosciences
Clinical Operations
FAX: 408-954-6313

10.5 Audits/Inspections

The site may be selected for an audit by the sponsor (e.g., BD Medical and Quality Assurance groups) or by a regulatory authority. The PI must facilitate the auditing process by granting all required access to the study site, study documentation, source data and study personnel. When the PI is notified of an upcoming audit by a regulatory authority, he/she must notify BD as soon as possible.

11.0 SAFETY REPORTING

Injuries or safety issues related to the use of an investigational study product(s) must be reported immediately to the Sponsor. This report should provide details of any unusual event concerning an operator/user of the device/system and the steps taken to provide treatment or abatement of any injuries or correction of the event. Upon receipt, the Sponsor will evaluate each report to determine if the event could affect the integrity of the study data or the operation of the system, or if it meets criteria of an "Adverse Event" as determined by BD Medical and Regulatory Affairs. All Serious Adverse Events (SAE) must be reported by the Principal Investigator to the IRB/IEC (and, if appropriate, any regulatory agency) within the specified time periods described in local/country regulations.

All safety events occurred during the conduct of the study will be reported to Sponsor using the appropriate form.

12.0 STUDY SUSPENSION OR TERMINATION

Should a determination be made that the study should be suspended or terminated at one or all sites (for example, by the Sponsor, by IRB/IEC request), then:

- new specimen enrollment shall be suspended or terminated at said site(s),
- enrolled specimens will be tested according to the protocol, which may be amended to accommodate study suspension or termination,
- the Sponsor shall promptly inform the Investigators and the IRB/IEC of the suspension or termination and the reason(s) for it.

13.0 STUDY ETHICS/ GOOD PRACTICES

The procedures set forth in this study protocol are designed to ensure that BD and clinical investigators abide by the ICH GCP Guidelines, regulations of the FDA, and the Declaration of Helsinki in the conduct, evaluation and documentation of the study. **In Kenya,** this study will be carried out in an ISO 15189 accredited laboratory and all procedures will follow due standard specifications.

13.1 Ethics Review and Approval

Before the start of specimen enrollment, all required documents will be submitted by site personnel to the KEMRI Ethical Review Committee (ERC) and Scientific Steering Committee (SSC) with respect to ethical and scientific compliance with applicable research and human subject's regulations.
The IRB/IEC must have the Federalwide Assurance (FWA) for the protection of Human Subjects for Domestic (U.S.) Institutions.

Before the start of specimen enrollment and testing, BD must have the following documents from the clinical site:

- CVs signed and dated from the Principal investigator and site staff participating in the trial (Sub-Investigator, Technologists, etc.)
- Approved Investigator's Agreement
- Principal investigator and Sub-Investigator financial disclosure
- EC/IRB approval letter for protocol
- EC/IRB approval for Informed consent

Clinical site personnel will inform BD that the EC/IRB approval has been obtained before any testing under this protocol can be performed. A copy of the EC/IRB approval letter will be placed in the study binder. Protocol amendments will be approved by the reviewing EC/IRB before implementation.

In view of the risk of new equipments being recalled after being implemented in certain regions due to lack of prior adequate validations, this study seeks to conduct a rigorous validation of the new BD FACSPresto (CD4 Point of Care device) system to ensure its efficiency in the low income countries.
Adequate measures will be put in place to protect the privacy of the study participants. Specimens will be de-identified upon participants' enrollment in the study and all related and resulting reports, documents and data shall be produced and maintained in such a way as to ensure their control and the protection of subject privacy as far as is reasonably practicable.

Findings from this study will be presented at study sites, in conferences and publication(s) and feedback to the community given as needed or requested by the community though KEMRI.

Any matters of intellectual property right will be handled according to KEMRI guidelines.

13.2 Informed Consent

Venous and Capillary Blood Specimens
The site will collect specimens prospectively from HIV infected subjects (**Phase I, II & phase III objective 1& 2) and HIV negative subjects (Phase III objective 3)** under EC/IRB approved informed consent. The principal investigator will prepare and submit the informed consent for EC/IRB review and also translate all the consent documents into the local language and verify the accuracy of the translation by performing independent back-translation. The EC/IRB may require that additional information be included in the consent form(s). The site will provide a copy of the EC/IRB documentation (i.e. approval letter) to BD.

Written informed consent must be obtained before the donor participates in the study. The clinical site is responsible for obtaining consent in accordance with the country regulations, state, local laws and internal or facility procedures (SOPs). A thorough explanation of study procedures and implications of study participation (risks/benefits) will be provided. If the potential participant is younger than 18 years of age and not a mature minor (see below), the study details will be explained to at least one parent/guardian for parental permission and then to the minor for assent. Mature minors are defined in the Kenya National VCT guidelines as persons under 18 years of age who are pregnant, married, or a parent, and who are able to consent for themselves. However, mature minors will be strongly advised to speak with and involve their parents/guardians in the study participation process if they feel it is safe to do so. Consent may be withdrawn at any time by the donor/participant. BD can withdraw donors enrolled in the study at any time.

13.3 Risks and Benefits

Venous and Capillary Blood Specimens
There are minimal medical risks to a subject other than a possible mild discomfort or bruising as can be expected with normal venous and capillary blood collection procedures. Phlebotomy will be performed by experienced clinical staff and particular attention will be made to avoid discomfort. Additionally, there is no risk of disclosure of confidential personal health information because the specimens will be de-identified upon enrollment in the study. There are no direct benefits to the subject for participation in this study and no investigational results will be reported.

13.4 Donor Identification and Confidentiality

Personal information will be maintained confidential in accordance with the site's procedures. Donors will be assigned a unique identification number when making the donation and recoded in a coded list or maintained according to the site policy for de-identified specimens. The coded list will remain with the clinical site or principal investigator or the site specific policy will be followed. All records that contain names or other personal identifiers, and informed consent forms, will be stored separately from study records identified by code number and will have limited access. All databases will be password-protected for security of access. Forms, lists, logbooks, and any other listings that link participant ID numbers to other identifying information will be stored in a separate, locked file in an area with limited access only by the data manager and principal investigator or appropriate designee.

Participant's study information will not be released outside of the study without the written permission of the participant, except as necessary for monitoring by KEMRI ERC and SSC, and regulatory authorities.

13.5 Donor Honoraria

Eligible persons enrolled in the study will also receive an honorarium in appreciation for their time and effort to complete the questionnaire and blood donation for the study. Consistent with local standards of practice for honoraria in other KEMRI/CDC studies and activities, the study proposes to offer enrolled participants with a bar of soap and 300 Kenya shillings (approximately US$ 4) to cover estimated costs for travel.

13.6 Study Limitations

- The scope of the study design is to validate that the results from the new system are equivalent to the predicates. The study does not attempt to address patient treatment or the effect of patient treatment.
- The stability section of the study addresses stability for capillary blood and venous blood collected in EDTA within 24 hours. There is no intent for extending the time window.
- All the Accuracy and Stability testing will be done in a controlled environment with the KEMRI staffs who are experienced in working with capillary blood collection and flow cytometry methodology and not necessarily the end user in low level health facilities.

14.0 INVESTIGATOR

14.1 Clinical Investigators

The Clinical Investigator and staff will meet GCP requirements. The Investigator, approved sub-Investigators, study operators and all supporting staff at the Investigator's site must be appropriately qualified by education and / or experience to perform their tasks.

14.2 Submission and obtaining Ethics Approval

The principal investigator must ensure to obtain the initial and at least annual approval from the IRB/ IEC for approving clinical studies. Furthermore, the study must not begin until the ethics approval letter is received by BD.

The principal investigator will notify the IRB/IEC the end of study, after close-out visit or during annual review.

14.2.1 Source Documents

The Investigator agrees that the Study monitors, Sponsor's employees, contractors or designees, as well as any regulatory bodies as required, will have the right to audit and review pertinent medical and study records relating to this clinical trial. The Investigator and staff will assist with the production, review, interpretation and / or correction of such records as required.

14.2.2 Device Accountability

Investigator or designee will maintain an accurate Accountability Log(s) and records of the investigational product received, used and unused. Study supplies must be used only for specimen enrollment and testing described in this protocol.

Principal investigator must supervise the use of investigational device only for the purpose of this study and under the Investigator's supervision. An Investigator shall not supply an investigational device to any person not authorized to receive or use it.

14.2.3 Data Recording and Record Retention

The Investigator(s) will ensure that the Case Report Forms, instrument records/ printouts, medical records (if applicable) and other study documents are maintained in a secure and confidential manner. The Investigator(s) will ensure that the medical records, Case Report Forms and other study documents are made available for review by the study monitor and any government regulatory bodies as required.

The Sponsor or Sponsor's designee will collect the original copy of the CRF.

A copy of all essential study documentation (e.g., CRFs, other data collection forms, instrument printouts, and electronic files) must be retained by the PI for a minimum of two years after notification from BD that the study is completed, terminated, or that the investigational product has been successfully released for commercialization, whichever is the longer time period. In the instance where the study PI withdraws as the responsible party, written notification containing the contact information of the PI designee must be forwarded to BD.

As the study sponsor, BD will retain all essential study documentation indefinitely (either in-house or off-site).

14.3 INVESTIGATOR REPORTS

14.3.1 Withdrawal of EC/IRB Approval

The Investigator shall report to Sponsor within 5 working days if, for any reason, the IRB/IEC withdraws approval to conduct the investigation, including a complete description of the reason(s) for which approval was withdrawn.

14.3.2 Deviation from the Study Protocol

The Investigator shall notify Sponsor and the reviewing EC/IRB of any changes in, or deviations from the Protocol. Such notice shall be given as soon as possible, but in no event later than 5 working days. Prior approval by Sponsor is required for changes in or deviations from the protocol. If such changes or deviations may affect the scientific soundness of the plan, or the rights, safety or welfare of the study subjects, then FDA and IRB/IEC approval is also required.

14.3.3 Use of Device without Informed Consent

No specimen will be tested with the investigational device without prior written Informed Consent, when applicable. If Investigator does proceed; the Investigator must report this use to BD and the IRB/IEC within 5 working days after it occur.

14.3.4 Final Report

If applicable, the Investigator will inform the institution of the termination of the trial, and should provide BD with all required reports, and provide the EC/IRB with a summary of the trial's outcome upon completion of the study.

14.3.5 Other Reports

Upon request of the Sponsor, FDA or IRB/IEC, the Investigator shall provide the stipulated information in accurate, complete and current form.

14.4 Responsibility for Investigator Reports

The Principal Investigator may delegate a qualified associate(s) to complete one or more of the above functions. However, the Principal Investigator retains the overall responsibility for the accuracy and

timeliness of any delegated report, proper conduct of the study including obtaining subject consent, compliance with this study plan, and the collection of all required data, as well as subject safety where applicable.

14.5 Financial Disclosure

A clinical Investigator shall disclose to the Sponsor sufficient accurate financial information to allow the applicant to submit complete and accurate certification or disclosure statements required by the regulations. The Investigator shall promptly update this information if any relevant changes occur during the course of the investigation and for 1 year following completion of the study.

15.0 SPONSOR

15.1 Sponsor Staff Qualifications

The BD Clinical Operations employees and contractors, including study monitors, will be appropriately qualified by education and / or experience to perform their tasks. Resources, systems, standard operating procedures and training adequate to oversee the scientific, ethical and regulatory aspects of the clinical study will be maintained throughout the period of the clinical investigation.

15.2 Beginning the Study

Sponsor shall not begin an investigation or part of an investigation until EC/IRB approval has been obtained. A copy of the EC/IRB written approval will be forwarded to BD.

15.3 Selecting Investigators

Sponsor shall select Investigators qualified by training and experience to investigate the Accuracy Evaluation of the Investigational BD FACSPresto System: Instrument, Software, and BD CD4/Hb Assay.

15.4 Ensuring Proper Monitoring

Sponsor shall select monitor(s) qualified by training and experience to monitor the progress of the investigation.

15.5 Ensuring Proper Notification

Sponsor shall ensure that any significant new information arising during the investigation is provided to all reviewing IRB/IEC, regulatory body and Investigators.

15.6 Control of Device

Sponsor shall ship investigational devices only to qualified Investigators participating in the study.

15.7 Obtaining Agreements

Sponsor shall obtain a signed and dated Investigator Agreement; the Investigator’s CV; a statement of the Investigator’s relevant experience (if applicable), including dates, location and type of experience; explanation of circumstances leading to termination of studies previously undertaken by the Investigator (if applicable); a statement that the Investigator is committed to conduct the investigation in accordance with the agreement, the investigational plan, other applicable FDA regulations, and other conditions imposed by the Ethics Committee/FDA; an agreement that the Investigator will supervise all testing of the investigational device involving donors and will ensure that the requirements for obtaining informed consent are met.

15.8 Securing Compliance

Sponsor must ensure that the Investigator continues to comply with the signed agreement, the investigational plan, other applicable FDA regulations and other conditions imposed by the Ethics Committee/FDA, or discontinue shipments of device to the Investigator, terminate the Investigator's participation in the investigation and require such an Investigator to return device, unless this action would jeopardize the rights, safety or welfare of a patient.

15.9 Resumption of Suspended or Terminated Studies

Sponsor may not resume a suspended or terminated investigation without Ethics Committee and any necessary country regulatory approval.

15.10 Reporting

A Final Study Report meeting the requirements of ISO 14155:2011, Annex C and relevant country regulations will be prepared by the Sponsor.

15.11 Records

Sponsor or delegate shall maintain accurate, complete, and current study documentation.

16.0 PUBLICATION

Publication of the study results in the medical literature will be attempted, under the authorship of the Investigators; however; BD reserves the right to comment on publications prior to submission in order to protect intellectual property rights and confidential information.

17.0 REFERENCES

1. E6, International Conference on Harmonization: Good Clinical Practice: Consolidated (Published in the Federal Register May 9, 1997).
2. Estaquier J, Idziorek T, de Bels F, Barré-Sinoussi F, Hurtrel B, Aubertin AM, Venet A, Mehtali M, Muchmore E, Michel P, Mouton Y, Girard M, Ameisen JC. Programmed cell death and AIDS: significance of T-cell apoptosis in pathogenic and nonpathogenic primate lentiviral infections. Proc Natl Acad Sci U S A. 1994 Sep 27;91(20):9431-5.
3. Antiretroviral Therapy of HIV Infection in Adults and Adolescents: Recommendations for a Public Health Approach. World Health Organization, 2010 revision.
4. Volberding PA, Lagakos SW, Koch MA, Pettinelli C, Myers MW, Booth DK, Balfour HH Jr, Reichman RC, Bartlett JA, Hirsch MS, Zidovudine in asymptomatic human immunodeficiency virus infection. A controlled trial in persons with fewer than 500 CD4-positive cells per cubic millimeter. The AIDS Clinical Trials Group of the National Institute of Allergy and Infectious Diseases. N Engl J Med. 1990 Apr 5;322 (14):941-9.
5. Cohen MS, Chen YQ, McCauley M, Gamble T, Hosseinipour MC, Kumarasamy N, *et al*. Prevention of HIV-1 infection with early antiretroviral therapy. *N Engl J Med* 2011; **365(6)**:493-505.
6. Thomas TK, Masaba R, Borkowf CB, Ndivo R, Zeh C, Misore A, *et al*. Triple-antiretroviral prophylaxis to prevent mother-to-child HIV transmission through breastfeeding--the Kisumu Breastfeeding Study, Kenya: a clinical trial. *PLoS Med* 2011; **8(3)**:e1001015.

7. Henry DH, Beall GN, Benson CA, Carey J, Cone LA, Eron LJ, Fiala M, Fischl MA, Gabin SJ, Gottlieb MS, et al. Recombinant human erythropoietin in the treatment of anemia associated with human immunodeficiency virus (HIV) infection and zidovudine therapy. Overview of four clinical trials. Ann Intern Med. 1992 Nov 1;117(9):739-48.
8. Firnhaber C, Smeaton L, Saukila N, Flanigan T, Gangakhedkar R, Kumwenda J, La Rosa A, Kumarasamy N, De Gruttola V, Hakim JG, Campbell TB. Comparisons of anemia, thrombocytopenia, and neutropenia at initiation of HIV antiretroviral therapy in Africa, Asia, and the Americas. Int J Infect Dis. 2010 Dec; 14(12):e1088-92. Epub 2010 Oct 18.
9. **CLSI.** Method Comparison and Bias Estimation Using Patient Samples; Approved Guideline-Second Edition. CLSI document EP9-A2; 2002.
10. Ernst DJ, Balance LO, Calam, RR, McCall R, Szamosi DI, Tyndall L.CLSI. Procedures and devices for the collection of diagnostic capillary blood specimens; Approved Guideline- Sixth Edition. CLSI document H04-06. Wayne, PA: Clinical and Laboratory Standards Institute; 2008.
11. Zeh C, Amornkul PN, Inzaule S, Ondoa P, Oyaro B, et al. (2011) Population-Based Biochemistry, Immunologic and Hematological Reference Values for Adolescents and Young Adults in a Rural Population in Western Kenya. PLoS ONE 6(6): e21040. doi:10.1371/journal.pone.0021040
12. Sekesai M, Memory C, Douglas M, Owen M, Stephano G, Karin H, Alexio M, Gaurav B,. Jonathan L, Trevor P. Evaluation of the PIMA Point-of-Care CD4 Analyzer in VCT Clinics in Zimbabwe. J Acquir Immune Defic Syndr 2010;55:1–7.

18.0 BUDGET

		Kshs	USD
Total Personnel			
Equipment			
Lab Supplies			
Telecommunication Supplies			
Office Supplies			
Travels			
Contractuals			
Others: A1			
Total Direct Costs			
BASE (% of Total Direct)	20%		

Grand Total Cost of Project	KES 11,270,000.00	$140,875

Justification of the budget

Personnel: In order to enroll 624 study participants at different time intervals over a period of 28 weeks, we will need 1 administrative officer, 1 clinical officer/study coordinator, 1 nurse-counselor, 4 community interviewers staff, 1 laboratory technologist and 1 QA Officer.

Supplies: Office supplies for the clinic will be bought. Some lab supplies will also have to be bought and this is included in the budget.

Travel: Some expenses will be incurred for staff for work related meetings and activities.

Contractual services: A lot of communication and networking will have to be done between the staff and community members. Scratch cards for mobile phones will be supplied to staff for communication. Training and consultation meetings will be held with community members on periodical basis of which expenditures will be made.

The study will be required to print study documents such as reports and consent documents.

Other costs: We will need one vehicle to transport staff and supplies and conduct other study-related business (e.g. for the study clinic, for community mobilization and for participant tracking) on a daily basis. The total estimated mileage needed is 3 km. These vehicles will require periodic maintenance.

Staff will require to be trained and cross-trained on the study requirements in order to harmonize and build their capacities. Training costs cover training on SOPs and other training related to study procedures.

APPENDICES

1. **Study Sites**
2. Study Screening and Eligibility Form (to be completed during screening)
3. Enrollment Questionnaire for adult and children
4. Adult Informed Consent Form (Consent 01)
5. Parental/Guardian Informed Consent Form (Consent 02)
6. Minor Assent Form (Assent 03)
7. **Donor Questionnaire**
8. Release of Medical Information Form for Adults
9. Release of Medical Information Form for Minors/Children
10. **Methods for Capillary blood Collection**

APPENXIX 1 – STUDY SITES

Investigator	Site	Address
Dr. Clement Zeh	KEMRI/CDC Research and Public Health Collaboration (KEMRI)	HIV Research Laboratory<br>ISO 15189 Accredited<br>KEMRI/CDC Research and Public Health Collaboration<br>PO Box 1578 Kisumu, Kenya<br><br>KEMRI/CDC Field Research Station<br>Busia-Kisumu Rd. (Opposite Kisian Railroad Station). Kisumu, Kenya
Dr. Mardhuri Takhar	National AIDS Research Institute (NARI)	Serology and Immunology Department, National AIDS Research Institute73, 'G'-Block, MIDC, Bhosari,<br>Pune 411 026<br>Maharashtra, IndiaPost Box No: 1895
Dr. Kovit Pattanapanyasat	Siriraj Hospital	Faculty Of Medicine Siriraj Hospital, Mahidol University,<br>2 Prannok Road, Siriraj,<br>Bangkok,10700 , Thailand
Laurie Byrne	BD MedLab	BD Biosciences<br>2350 Qume Dr. San Jose,<br>California, 95131 USA

APPENDIX 2: STUDY SCREENING AND ELIGIBILITY FORM

(To be completed during screening)

TITLE OF STUDY: Evaluation of the Investigational BD FACSPresto™ System: Instrument, Software, and BD CD4%CD4/Hb Cartridge Assay

Screening date: ____DD-MMM-YYYY______ Screening ID: ____________

Health facility: ______________________ Patient Facility ID: ________________

Inclusion Criteria: ***Does potential participant/specimen meet the following criteria?***

Subject:

1. Has been infected with HIV **for phase I, II and III (objective 1&2) and persons for phase III (objective 3)** and willing to provide written informed consent/assent and if minor/child, parent(s)/legal guardian are willing to provide informed consent to draw venous and capillary blood from the child ?. yes/no
2. Allows access to her medical records and agrees to disclose age and gender ? yes/no (if yes___ date of birth? _____ Gender? ____)
3. Agrees to disclose co-morbid conditions information: malaria, tuberculosis, anemia, sickle cell anemia, infection diseases, thalassemia and other current medical conditions ? yes/no

Is the Specimen:

1. Venous blood that meets requirements for specimen donation as required. yes/no
2. Venous blood collected in a blood collection tube with EDTA anticoagulant and stored at room temperature (20-25°C) and according to collection tube manufacturer's guidelines until enrollment ? yes/no
3. Venous blood drawn within an adequate time to perform post-enrollment staining within 24 hours? yes/no
4. Of acceptable quality for flow cytometry testing (e.g., no hemolysis or clots and acceptable pre-analytical handling)? yes/no
5. Venous blood of sufficient residual volume: >1 mL for Sample Preparation? yes/no
6. Capillary blood that meets requirements for specimen donation as required?. yes/no
7. Capillary blood applied onto the PEO/IUO BD CD4/%CD4/Hb cartridge? yes/no
8. Capillary blood specimen draw date and time is recorded yes/no
9. Signed Consent? Yes/no

All of the following criteria must be met for the subject or the peripheral residual whole blood specimen to be enrolled into this study:

**10. The potential participant is:**	Eligible **yes/no (proceed to question 11)**<br>Not Eligible **yes/no (Stop, do not enroll)**

11. Study ID ***(If ALL the above are answered "Yes", then assign study number , collect/have the specimen collected and complete the enrollment questionnaire) Study ID*** ___________

12. Enrollment date: ___DD-MMM-YYYY__

APPENDIX 3: ENROLLMENT QUESTIONNAIRE FOR ADULT AND CHILDREN

TITLE OF STUDY: Evaluation of the Investigational BD FACSPresto™ System: Instrument, Software, and BD CD4%CD4/Hb Cartridge Assay

Enrollment date: ____DD-MMM-YYYY______

Health facility: ________________________ ________________

Study ID; _________________________ **Screening ID: _______________________**

Date of Birth: ___DD-MMM-YYYY______ Sex (gender): _____________

Date of specimen collection ___DD-MMM-YYYY______ Time of collection _____Hr: Min

Hand used for capillary blood collection: ___________________

Hello, thank you for agreeing/allowing your child to be in this study. I'd like to ask you a few questions about yourself/your child.

1. When did you find out you/your child had HIV? _____/_____ (mo/yr)

2. Are you/or your child currently taking any of the following medications? Check all drugs being taken
   a. Antiretroviral drugs **yes/no**
   b. Antimalarial drugs **yes/no**
   c. Anti tuberculosis drugs **yes/no**
   d. Other: ______________________

   } **if yes, please specify bellow**

Specify:____
____
____
____

3. Have you/your child ever been diagnosed with the following conditions in the last three months? Check all that apply
   a. Anemia
   b. Sickle cell Anemia
   c. Tuberculosis
   d. Malaria
   e. Pneumonia
   f. Other (specify): ____

Thank you for your time. Do you have any questions that I can answer?We appreciate you being a part of this study.

APPENDIX 4: ADULT INFORMED CONSENT FORM (CONSENT 01A)

This form covers the adult HIV Positive Male and Female (18 yrs or older) registered at an HIV Care and treatment facility in Western Kenya.

TITLE OF STUDY: Evaluation of the Investigational BD FACSPresto™ System: Instrument, Software, and BD CD4%CD4/Hb Cartridge Assay

BACKGROUND

You have the Human Immunodeficiency Virus (HIV) which is the virus that causes Acquired Immunodeficiency Syndrome (AIDS) and you are registered for care at this HIV care and treatment facility. You and your doctor have agreed that it is time for you to take a CD4 test and because you are taking the test, you are being asked to be in a research study to validate the performance of a new CD4 and Hemoglobin testing device. This study will evaluate the performance of a new investigational portable and point of care CD4 and Hemoglobin testing device against the current devices being used to test for CD4 and Hemoglobin in the HIV care and treatment hospitals/laboratories in Western Kenya. This study is from the U.S. Centers for Disease Control and Prevention (CDC), the Kenya Medical Research Institute (KEMRI), and the Kenya Ministry of Health (MOH).

In the study, 5 milliliters of blood (mL) will be collected from your arm (vein) and finger (via finger stick) and will be given an identity that is unique and different from the identity on your health/clinic records and sent to the KEMRI/CDC HIV-Research laboratory at the Jaramogi Oginga Odinga Teaching & Referral Hospital for testing.

Before you choose to be in this study, you need to know about any good or bad things that could happen if you decide to join. This form tells you about the study, you can ask any questions you have at any time

BEING IN THE STUDY IS YOUR CHOICE

This consent form tells you about the study. This information will be discussed with you. Once you know about the study and decide if you want to be in this study, you will be asked to sign this consent form and we will give you a copy to keep.

Before we tell you more about the study, you should know that:

- It is your choice to join.
- If you choose to be in the study, you can withdraw at any time.
- If you choose to be in the study, will be asked to respond to certain questions
- You may choose not to join the study.
- If you choose not to be in the study, you will still get your regular CD4 tests and the usual care.

<u>The goal of this study is to:</u>

1. **Asses the performance of the new, lighter and easy to carry CD4 and Hb testing device also known as the "BD FACSPresto™ System and CD4/%CD4/Hb cartridge" against that of the tests currently approved and being used in hospitals and laboratories to test for CD4 (called FACSCalibur flow cytometer) in blood collected from the veins (arms) and from finger pricks. We will place a unique number/code on the collected blood specimen so as to protect the participant's right to privacy and confidentiality. We will also find out the level of CD4 and**

Hb that normal healthy people have (also known as normal reference levels) and this would in future guide the grouping or classifying of CD4 and Hb results from the point-of-care testing devices as either high or low.

2. **Asses the performance of the new, lighter and easy to carry CD4 and Hb testing device against that of the tests currently approved and being used in hospitals and laboratories to test for Hemoglobin (Hb) levels.**

3. **To find out from those (customers) who conduct CD4 and Hb tests using the BD FACSPresto system how easy or challenging it is to do this.**

WHAT HAPPENS DURING THE STUDY

- When you join the study, a member of the study staff will ask you questions about your background, age, illness and medication history. Participation in the study is once.
- For the study, about one teaspoon (4mL) of blood will be taken from a vein in your arm and from your finger when you join the study.
- As part of regular care people with HIV and those on ARVs should have regular blood tests done. If your doctor requests these to be done, the blood will be taken at the same time as the blood for the study test which will be sent to KEMRI/CDC HIV R Lab at the **Jaramogi Oginga Odinga Teaching & Referral Hospital (JOOTRH** formerly Nyanza Provincial Hospital) for processing.
- During this study, you will continue to visit the clinic for your regular care. Being in this study will take a little extra time (approximately 55 minutes) but your participation will only be required once.
- After each clinic visit we will look at your medical record. This is to help us learn about your illness and medication history. All the information will be kept private.
- All specimens in the study will be given an identity that is unique and different from the patient's personal health information by the assignment of a study-specific specimen ID.

IF YOU CHOOSE TO LEAVE THE STUDY

You can choose not to participate in this study. This will not affect the health care or HIV medicine you get in the future.

RISKS TO YOU

There are minimal medical risks to you other than a possible mild discomfort or bruising as can be expected with normal venous and capillary (finger stick) blood collection procedures. Additionally, there is no risk of disclosure of confidential personal health information because the specimens will be uniquely identified upon enrollment in the study.

BENEFITS TO YOU

There are no direct benefits to you for participation in this study other than the opportunity to contribute to the performance evaluation of a new investigational Point of Care CD4 testing device. No investigational results will be reported.

REASONS FOR TAKING YOU OUT OF THE STUDY WITHOUT YOUR CONSENT

You may be taken out of the study without your consent if:

- Your doctor decides that being in the study may cause harm to you;
- Your specimen is found to be unsuitable for testing i.e. **if it has clotted**

- The study is stopped by sponsor, KEMRI, or MOH;
- Other reasons beyond our control.

COSTS TO YOU

- There is no cost to you for participating in the study
- Upon participation in the study, you will receive a token of a bar of soap as appreciation and 300 Kenya shillings (approximately US$ 4) to cover estimated costs for travel.

YOUR RECORDS WILL BE PRIVATE

Your research records will be kept private as allowed by the law. Your study record will have a code number. Any information from your records will not be given out unless you agree in writing. Your name will not be in any reports about this study. People who review the study for KEMRI and the sponsor may need to look at your study records from time to time. For study needs, we will make copies of your medical records. All copies will be kept in a locked file cabinet. Only study staff will have access to this cabinet.

BLOOD SAMPLES

Your blood samples or specimen will be stored in Kenya, at the KEMRI/CDC laboratories, for the duration of the study. They will be destroyed once the study is completely over.

PROBLEMS OR QUESTIONS

For questions about this study or a research-related injury, contact:

- Frank Angira at the KEMRI/CDC study clinic at Jaramogi Oginga Odinga Teaching & Refferral Hospital (JOOTRH) off Kakamega Road, Kisumu (P.O. Box 1578, Kisumu) or phone number: 057-2022929 Ext 619, Email: fangira@kemricdc.org

- Dr. **John Vulule Principal Investigator and** Director KEMRI/CDC Field Station, KEMRI, Center for Global Health Research, off Kisumu-Busia Road, Kisumu) Tel: 0572022983/2022959/2022902 KEMRI/CDC, P.O. Box 1578, Kisumu, Kenya

For questions about your rights as a research subject, contact:

The Secretary, **KEMRI Ethics Review Committee**
P.O. Box 54840-00200, Nairobi, Kenya
Telephone: 020-2722541, 0722 205901, 0733 400003
Email Address : **ERCadmin@kemri.orgt**

Principal Investigator: Clement Zeh
Co-investigators: Lisa A. Mills, Imelda O. Zapata, Frank Angira, Benta Akoth, Boaz Oyaro, Oscar Segurado, Lorlelei Lee-Haynes, Jerry Zhang, Jeannine Paliotta, Henok Tilahun, Kayla Laserson, John Vulule

STATEMENT OF CONSENT:

Please sign your name below once you:

1. have read (have been explained) and know the reasons for the study,
2. know the steps to be followed in the study,
3. know the risks and benefits to you from being in the study,
4. AND choose to enroll in this study of your own free will.

**Volunteer's Name**<br>**Type or print**	***Volunteer's Signature***<br>(Volunteer does **NOT** sign here if she is under age 18)	**Date**

**Witness's Name**<br>**Type or print**	**Witness's Signature**	**Date**

I have explained the purpose of this study to the volunteer. To the best of my knowledge, she understands the purpose, procedures, risks and benefits of this study.

**Investigator/Designee Name**<br>***Type or print***	**Investigator/Designee Signature**	**Date**

NOTE: This consent form with original signatures must be retained on file by the principal investigator. A copy must be given to the volunteer. A copy should be placed in the volunteer's medical record (if applicable).

APPENDIX 5: PARENTAL/GUARDIAN CONSENT FORM (CONSENT 02A)

This form covers the HIV infected male and female children registered at an HIV Care and treatment facility in Western Kenya.

TITLE OF STUDY: Evaluation of the Investigational BD FACSPresto™ System: Instrument, Software, and BD CD4%CD4/Hb Cartridge Assay

BACKGROUND

Your child has the Human Immunodeficiency Virus (HIV) which is the virus that causes Acquired Immunodeficiency Syndrome (AIDS) and is registered for care at this HIV care and treatment facility. You and your doctor have agreed that it is time for him/her to take a CD4 test and because he/she is taking the test, you are being asked if your child may be in aresearch study to validate the performance of a new CD4 and Hemoglobin testing device. This study will evaluate the performance of a new investigational portable and point of care CD4 and Hemoglobin testing device against the current devices being used to test for CD4 and Hemoglobin in the HIV care and treatment hospitals/laboratories in Western Kenya. This study is from the U.S. Centers for Disease Control and Prevention (CDC), the Kenya Medical Research Institute (KEMRI), and the Kenya Ministry of Health (MOH).

In the study, 5 milliliters of blood will be collected from your child's arm (vein) and finger (via finger stick) and will be given an identity that is unique and different from the identity on his/her health/clinic records and sent to the KEMRI/CDC HIV-Research laboratory at the Jaramogi Oginga Odinga Teaching & Referral l Hospital for testing.

Before you allow your child to be in this study, you need to know about any good or bad things that could happen if you decide that your child joins the study. This form tells you about the study, you can ask any questions you have at any time

BEING IN THE STUDY IS YOUR CHOICE

This consent form tells you about the study. This information will be discussed with you. Once you know about the study and decide if you want your child to be in this study, you will be asked to sign this consent form and we will give you a copy to keep.

Before we tell you more about the study, you should know that:

- It is your choice to allow your child to be in the study.
- If you choose to allow your child to be in the study, you can withdraw him/her at any time.
- If you choose to allow your child to be in the study, will be asked to respond to certain questions.
- You may choose not to allow your child to join the study.
- If you choose not to allow your child to be in the study, your child will still get his/her regular CD4 tests and the usual care.

The goal of this study is to:

1. **Asses the performance of the new, lighter and easy to carry CD4 and Hb testing device also known as the "BD FACSPresto™ System and CD4/%CD4/Hb cartridge" against that of the tests currently approved and being used in hospitals and laboratories to test for CD4 (called FACSCalibur flow cytometer) in blood collected from the veins (arms) and from finger pricks. We will place a unique number/code on the collected blood specimen so as to protect the participant's right to privacy and confidentiality. We will also find out the level of CD4 and Hb that normal healthy people have (also known as normal reference levels) and this would in**

future guide the grouping or classifying of CD4 and Hb results from the point-of-care testing devices as either high or low.

2. **Asses the performance of the new, lighter and easy to carry CD4 and Hb testing device against that of the tests currently approved and being used in hospitals and laboratories to test for Hemoglobin (Hb) levels.**

3. **To find out from those (customers) who conduct CD4 and Hb tests using the BD FACSPresto system how easy or challenging it is to do this.**

WHAT HAPPENS DURING THE STUDY

- When your child joins the study, a member of the study staff will ask you questions about your child's background, age, illness and medication history. Participation in the study is once.
- For the study, less than a teaspoon (4ml) of blood will be taken from a vein in your child's arm and from his/her finger when he/she joins the study.
- As part of regular care children with HIV and those on ARVs should have regular blood tests done. If your child's doctor requests these to be done, the blood will be taken at the same time as the blood for the study test which will be sent to KEMRI/CDC HIV R Lab at the **Jaramogi Oginga Odinga Teaching & Referral Hospital (JOOTRH** formerly Nyanza Provincial Hospital) for processing.
- During this study, you will continue to visit the clinic for your regular care. Being in this study will take a little extra time (approximately 55 minutes) but your participation will only be required once.
- After each clinic visit we will look at your child's medical record. This is to help us learn about your child's illness and medication history. All the information will be kept private.
- All specimens in the study will be given an identity that is unique and different from the one in the patient's personal health information by the assignment of a study-specific specimen ID.

IF YOU CHOOSE TO TAKE YOUR CHILD FROM THE STUDY

You can choose not to participate in this study. This will not affect the health care or HIV medicine you get in the future.

RISKS TO YOUR CHILD

There are minimal medical risks to your child other than a possible mild discomfort or bruising as can be expected with normal venous and capillary (finger stick) blood collection procedures. Additionally, there is no risk of disclosure of confidential personal health information because the specimens will be uniquely identified upon enrollment in the study.

BENEFITS TO YOUR CHILD

There are no direct benefits to your child for participation in this study other than the opportunity to contribute to the performance evaluation of a new investigational Point of care CD4 testing device. No investigational results will be reported.

REASONS FOR TAKING YOUR CHILD OUT OF THE STUDY WITHOUT YOUR CONSENT

Your child may be taken out of the study without your consent if:

- Your child's doctor decides that being in the study may cause harm to him/her;
- Your child's specimen is found to be unsuitable for testing i.e. if it has clotted.

- The study is stopped by sponsor, KEMRI, or MOH;
- Other reasons beyond our control.

COSTS TO YOUR CHILD

- There is no cost to you for participating in the study
- Upon participation in the study, you/your child will receive a token of a bar of soap as appreciation and 300 Kenya shillings (approximately US$ 4) to cover estimated costs for travel.

YOUR CHILD'S RECORDS WILL BE PRIVATE

Your child's research records will be kept private as allowed by the law. Your child's study records will have a code number. Any information from your child's records will not be given out unless you agree in writing. Your child's name will not be in any reports about this study. People who review the study for KEMRI and the sponsor may need to look at your child's study records from time to time. For study needs, we will make copies of your child's medical records. All copies will be kept in a locked file cabinet. Only study staff will have access to this cabinet.

BLOOD SAMPLES

Your child's blood samples or specimen will be stored in Kenya, at the KEMRI/CDC HIV-Research laboratories, for the duration of the study. They will be destroyed once the study is completely over.

PROBLEMS OR QUESTIONS

For questions about this study or a research-related injury, contact:

- Frank Angira at the KEMRI/CDC study clinic at Jaramogi Oginga Odinga Teaching & Refferral Hospital (JOOTRH) off Kakamega Road, Kisumu (P.O. Box 1578, Kisumu) or phone number: 057-2022929 Ext 619, Email: fangira@kemricdc.org

- Dr. **John Vulule, Principal Investigator and** Director KEMRI/CDC Field Station, KEMRI, Center for Global Health Research, off Kisumu-Busia Road, Kisumu) Tel: 0572022983/2022959/2022902 KEMRI/CDC, P.O. Box 1578, Kisumu, Kenya

For questions about your rights as a research subject, contact:

The Secretary, **KEMRI Ethics Review Committee**
P.O. Box 54840-00200, Nairobi, Kenya
Telephone: 020-2722541, 0722 205901, 0733 400003
Email Address : **ERCadmin@kemri.orgt**

Principal Investigator: Clement Zeh
Co-investigators: Lisa A. Mills, Imelda O. Zapata, Frank Angira, Benta Akoth, Boaz Oyaro, Oscar Segurado, Lorlelei Lee-Haynes, Jerry Zhang, Jeannine Paliotta, Henok Tilahun, Kayla Laserson, John Vulule

STATEMENT OF CONSENT:

Please sign your name below once you:

1. have read (have been explained) and know the reasons for the study,
2. know the steps to be followed in the study,
3. know the risks and benefits to your child for being in the study,
4. AND choose to enroll your child in this study of your own free will.

______________________ **Child's Name** **Type or print**	______________________	______________________
______________________ **Name of Child's Parent or Legal Guardian** (must be over 18 years old) ***Type or print***	______________________ **Legal Guardian's Signature**	______________________ **Date**
______________________ **Witness's Name** ***Type or print***	______________________ **Witness's Signature**	______________________ **Date**

I have explained the purpose of this study to the volunteer. To the best of my knowledge, he/she understands the purpose, procedures, risks and benefits of this study.

______________________ **Investigator/Designee Name** ***Type or print***	______________________ **Investigator/Designee Signature**	______________________ **Date**

NOTE: This consent form with original signatures must be retained on file by the principal investigator. A copy must be given to the volunteer. A copy should be placed in the volunteer's medical record (if applicable).

APPENDIX 6: MINOR ASSENT FORM (ASSENT 03A)

This form covers the HIV Positive Male and Female registered at an HIV Care and treatment facility in Western Kenya. It must be signed if the potential participant is aged between 13 to 17 years. A legal guardian must sign the consent form.

TITLE OF STUDY: Evaluation of the Investigational BD FACSPresto™ System: Instrument, Software, and BD CD4%CD4/Hb Cartridge Assay

BACKGROUND

You have the Human Immunodeficiency Virus (HIV) which is the virus that causes Acquired Immunodeficiency Syndrome (AIDS) and you are registered for care at this HIV care and treatment facility. You and your doctor have agreed that it is time for you to take a CD4 test and because you are taking the test, you are being asked to be in research study to make valid the performance of a new CD4 and Hemoglobin testing device. This study will evaluate the performance of a new investigational portable and point of care CD4 and Hemoglobin testing device against the current devices being used to test for CD4 and Hemoglobin in the HIV care and treatment hospitals/laboratories in Western Kenya. This study is from the U.S. Centers for Disease Control and Prevention (CDC), the Kenya Medical Research Institute (KEMRI), and the Kenya Ministry of Health (MOH).

In the study, 5 milliliters of blood will be collected from your arm (vein) and finger (via finger stick) and will be given an identity that is unique and different from the one in your health/clinic records and sent to the KEMRI/CDC HIV-Research laboratory at the Jaramogi Oginga Odinga Teaching & Refferral Hospital for testing.

Before you choose to be in this study, you need to know about any good or bad things that could happen if you decide to join. This form tells you about the study, you can ask any questions you have at any time.

BEING IN THE STUDY IS YOUR CHOICE

This assent form tells you about the study. This information will be discussed with you. Once you know about the study and decide if you want to be in this study, you will be asked to sign this assent form and we will give you a copy to keep.

Before we tell you more about the study, you should know that:

- It is your choice to join.
- If you choose to be in the study, you can withdraw at any time.
- If you choose to be in the study, you will be asked to respond to certain questions
- You may choose not to join the study.
- If you choose not to be in the study, you will still get your regular CD4 tests and the usual care.

<u>The goal of this study is to:</u>

1. **Asses the performance of the new, lighter and easy to carry CD4 and Hb testing device also known as the "BD FACSPresto™ System and CD4/%CD4/Hb cartridge" against that of the tests currently approved and being used in hospitals and laboratories to test for CD4 (called FACSCalibur flow cytometer) in blood collected from the veins (arms) and from finger pricks. We will place a unique number/code on the collected blood specimen so as to protect the participant's right to privacy and confidentiality. We will also find out the level of CD4 and Hb**

that normal healthy people have (also known as normal reference levels) and this would in future guide the grouping or classifying of CD4 and Hb results from the point-of-care testing devices as either high or low.

2. **Asses the performance of the new, lighter and easy to carry CD4 and Hb testing device against that of the tests currently approved and being used in hospitals and laboratories to test for Hemoglobin (Hb) levels.**

3. **To find out from those (customers) who conduct CD4 and Hb tests using the BD FACSPresto system how easy or challenging it is to do this.**

WHAT HAPPENS DURING THE STUDY

- When you join the study, a member of the study staff will ask you questions about your background, age, illness and medication history. Participation in the study is once.
- For the study, less than a teaspoon (4mL) of blood will be taken from a vein in your arm and from your finger when you join the study.
- As part of regular care people with HIV and those on ARVs should have regular blood tests done. If your doctor requests these to be done, the blood will be taken at the same time as the blood for the study test which will be sent to KEMRI/CDC HIV-Research Laboratory at the **Jaramogi Oginga Odinga Teaching & Referral Hospital (JOOTRH** formerly Nyanza Provincial Hospital) for processing.
- During this study, you will continue to visit the clinic for your regular care. Being in this study will take a little extra time (approximately 55 minutes) but your participation will only be required once.
- After each clinic visit we will look at your medical record. This is to help us learn about your illness and medication history. All the information will be kept private.
- All specimens in the study will be given an identity that is unique and different from the one in your personal health information by the assignment of a study-specific specimen ID.

IF YOU CHOOSE TO LEAVE THE STUDY

You can choose not to participate in this study. This will not affect the health care or HIV medicine you get in the future.

RISKS TO YOU

There are minimal medical risks to you other than a possible mild discomfort or bruising as can be expected with normal venous and capillary (finger stick) blood collection procedures. Additionally, there is no risk of disclosure of confidential personal health information because the specimens will be uniquely identified upon enrollment in the study.

BENEFITS TO YOU

There are no direct benefits to you for participation in this study other than the opportunity to contribute to the performance evaluation of a new investigational Point of Care CD4 testing device. No investigational results will be reported.

REASONS FOR TAKING YOU OUT OF THE STUDY WITHOUT YOUR ASSENT

You may be taken out of the study without your assent if:

- Your doctor decides that being in the study may cause harm to you;
- Your specimen is found to be unsuitable for testing i.e. if it has clotted.
- The study is stopped by sponsor, KEMRI, or MOH;
- Other reasons beyond our control.

COSTS TO YOU

- There is no cost to you for participating in the study
- Upon participation in the study, you will receive a token of a bar of soap as appreciation and 300 Kenya shillings (approximately US$ 4) to cover estimated costs for travel.

YOUR RECORDS WILL BE PRIVATE

Your research records will be kept private as allowed by the law. Your study record will have a code number. Any information from your records will not be given out unless you agree in writing. Your name will not be in any reports about this study. People who review the study for KEMRI and the sponsor may need to look at your study records from time to time. For study needs, we will make copies of your medical records. All copies will be kept in a locked file cabinet. Only study staff will have access to this cabinet.

BLOOD SAMPLES

Your blood samples or specimen will be stored in Kenya, at the KEMRI/CDC laboratories, for the duration of the study. They will be destroyed once the study is completely over.

PROBLEMS OR QUESTIONS

For questions about this study or a research-related injury, contact:

- Frank Angira at the KEMRI/CDC study clinic at Jaramogi Oginga Odinga Teaching & Refferral Hospital (JOOTRH) off Kakamega Road, Kisumu (P.O. Box 1578, Kisumu) or phone number: 057-2022929 Ext 619, Email: fangira@kemricdc.org

- Dr. **John Vulule, Principal Investigator and** Director KEMRI/CDC Field Station, KEMRI, Center for Global Health Research, off Kisumu-Busia Road, Kisumu) Tel: 0572022983/2022959/2022902 KEMRI/CDC, P.O. Box 1578, Kisumu, Kenya

For questions about your rights as a research subject, contact:

The Secretary, **KEMRI Ethics Review Committee**
P.O. Box 54840-00200, Nairobi, Kenya
Telephone: 020-2722541, 0722 205901, 0733 400003
Email Address : **ERCadmin@kemri.orgt**

Principal Investigator: Clement Zeh
Co-investigators: Lisa A. Mills, Imelda O. Zapata, Frank Angira, Benta Akoth, Boaz Oyaro, Oscar Segurado, Lorlelei Lee-Haynes, Jerry Zhang, Jeannine Paliotta, Henok Tilahun, Kayla Laserson, John Vulule

STATEMENT OF ASSENT:

Please sign your name below once you:

5. have read (have been explained) and know the reasons for the study,
6. know the steps to be followed in the study,
7. know the risks and benefits to you from being in the study,
8. AND choose to enroll in this study of your own free will.

________________________	________________________	____________________
**Minor's name**<br>**Type or print**	**Minor's signature**	**Date**

________________________	________________________	____________________
**Witness's Name**<br>**Type or print**	**Witness's Signature**	**Date**

I have explained the purpose of this study to the volunteer. To the best of my knowledge, she understands the purpose, procedures, risks and benefits of this study.

________________________	________________________	____________________
**Investigator/Designee Name**<br>***Type or print***	**Investigator/Designee Signature**	**Date**

NOTE: This assent form with original signatures must be retained on file by the principal investigator. A copy must be given to the volunteer. A copy should be placed in the volunteer's medical record (if applicable).

APPENDIX 7: DONOR'S QUESTIONNAIRE

Date: (DD/MMM/YYYY) _______/________/_______

Subject ID:____________

Age____________ Sex: M F Weight_________ Kg Height: _________m

1. Do you consider yourself healthy? Y N
2. Do you exercise regularly? Y N

   How often (hr/wk)? _______________________

   Degree of activity: (light) 1 2 3 4 5 6 7 8 9 10 (Vigorous)

3. Have you been sick recently? Y N

   If YES, when? ________________________ Describe illness________________________

4. Are taking any prescribed medications? Y N

   If YES, what?

5. Do you have high blood pressure? Y N
6. Do you take vitamin supplements or herbal remedies? Y N

   If YES, what? _____________________________

7. Are you exposed to any hazardous chemicals in your job? Y N

   If YES, what? _______________________________

8. Do you use tobacco? Y N

   If YES, in what form?________________________ How often?_______________________

9. Do you eat special diet? Y N

   If YES, please describe?_____________________________________

10. Do you drink alcohol? Y N

    If YES, in what form?_____________ How often?_____________

11. Are you currently under a Doctor's care? Y N

    If YES, why? ________________________

12. Have you been hospitalized recently? Y N

If YES, why? ________________________ When? ________________________

13. Are there any inherited health disorders in your family? Y N

    If YES, please describe? __________________________

14. Have you taken aspirin or any pain medication recently? Y N

    If YES, What?________________________ When? ________________________

15. Have you taken any cold or allergy medicine recently? Y N

    If YES, What? ________________________ When? ________________________

16. Are taking diet pills?

For Women

1. Are you still menstruating? Y N

   If YES, when was your last period?

   If NO, are you on hormone replacement therapy? Y N

2. Are you breastfeeding? Y N
3. Are you pregnant? Y N

   If YES, what is your due date?

4. Are you using contraceptives? Y N

APPENDIX 8: RELEASE OF MEDICAL INFORMATION FOR ADULTS (ENGLISH)

TITLE OF STUDY: Evaluation of the Investigational BD FACSPresto^{TM} System: Instrument, Software, and BD CD4%CD4/Hb Cartridge Assay

Date: ___________________________

Patient Facility ID: ______________________

Patient Name: _________________________________

Facility: ______________________________________

I am currently a patient registered at _______________________________ for my HIV care and treatment. I am now also enrolled in the Evaluation of the Investigational BD FACSPresto^{TM} System: Instrument, Software, and BD CD4%CD4/Hb Cartridge Assay study. I give my permission to release to the study team for a period of 6 months from:
[Enrollment date (dd/mmm/yyyy)]:______________ ____
to [6 months later (dd/mmm/yyyy)]:_________________
all findings and information in connection with my examinations, care, treatment, and diagnosis.

I understand that I may change my mind and withdraw my consent to participate in the study in which case my records should no longer be released to the study staff. I also understand that information obtained will be kept strictly confidential and will be used only for study purposes.

Patient's signature/thumbprint: ___________________________________

Date: ______________

Witness: ____

Date: _____________

Study staff obtaining permission for release of medical information:

Name: _____________________________

Signature:___________________________

Date: _____________

APPENDIX 9: RELEASE OF MEDICAL INFORMATION FOR CHILDREN (ENGLISH)

TITLE OF STUDY: Evaluation of the Investigational BD FACSPresto^{TM} System: Instrument, Software, and BD CD4%CD4/Hb Cartridge Assay

Date: __________________

Patient Facility ID: ______________

Patient (Child) Name: ________________________

Facility: _______________________

My child is currently a patient registered at _______________________________ for HIV care and treatment and now also enrolled in the Evaluation of the Investigational BD FACSPresto^{TM} System: Instrument, Software, and BD CD4%CD4/Hb Cartridge Assay study. I give my permission to release to the study team for a period of 6 months from:
[Enrollment date (dd/mmm/yyyy)]:______________ ____
to [6 months later (dd/mmm/yyyy)]:_________________
all findings and information in connection with my child's examinations, care, treatment, and diagnosis.

I understand that I may change my mind and withdraw my consent for my child to participate in the study in which case my child's records should no longer be released to the study staff. I also understand that information obtained will be kept strictly confidential and will be used only for study purposes.

Parent/Guardian's name:_______________________________________

Parent/Guardian's signature/thumbprint: ___________________________________

Date: ______________

Witness: ____

Date: _____________

Study staff obtaining permission for release of medical information:

Name: ____________________________

Signature:___________________________

Date: _____________

APPENDIX 10: METHODS FOR CAPILLARY BLOOD COLLECTION

Capillary blood collection will be done according to the site approved standard procedures and CLSI H04-A6[9] and the method of capillary blood collection as shown in this appendix.

- Ensure all materials for capillary blood collection are assembled
  - Gloves, warming device (moist towel or other warming device), 70% isopropyl alcohol swabs, Contact-Activated Lancets, pre-labeled PEO/IUO CD4/Hb cartridges and cuvettes
- Wash hands thoroughly and put on gloves.
- Select the site
  - Patient should be sitting or lying down
  - Have patient hold their hand in a downward position, allowing gravity to increase blood supply to the hand
  - Select middle or ring finger. Identify the desired puncture site (shaded area).

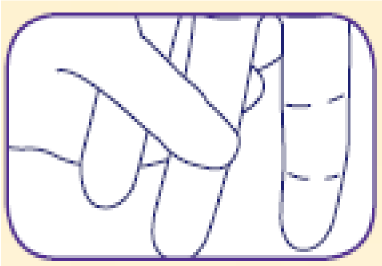

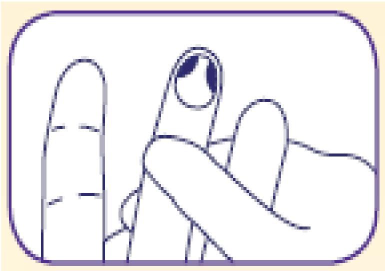

- Disinfect the site (Air dry) cleaning the puncture site using alcohol pad and according to the facility established procedure. The site must be allowed to air dry in order to provide effective disinfection and to prevent possible hemolysis or erroneous results from residual alcohol.
- Remove protective tab from the lancet. Twist off the tab to break the seal and discard
- Perform the puncture. Position the lancet firmly against the puncture site as illustrated. Hold the lancet between fingers.
- To activate, press lancet firmly against the puncture site. Do not remove the device from the site until an audible click is heard

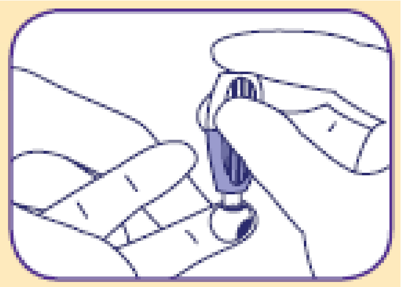

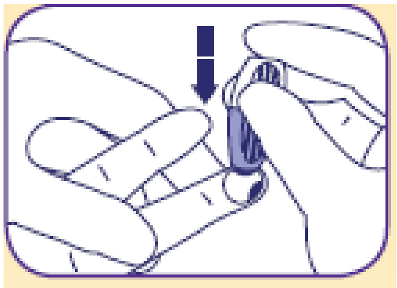

- Discard used lancets into sharp containers according to the facility established procedures
- Collect specimen by gently applying intermittent pressure along finger capillaries up to the puncture site. Hold hand below elbow level to obtain the required blood volume. Strong repetitive pressure (milking) **must** be avoided; it may cause hemolysis or tissue-fluid contamination of the specimen.

- Wipe away the first drop if blood with sterile gauze pad, as this drop may contain an excess of tissue fluids that could cause erroneous results.
- Turn the patient's hand palm down.
- Position the PEO/IUO CD4/Hb cartridge directly beneath the puncture site. Apply blood to the port of the first PEO/IUO CD4/Hb cartridge, ensure that blood is flowing into the channel.
- Repeat applying a drop of blood to the second PEO/IUO CD4/Hb cartridge, ensure blood flows into the channel
- Apply a drop of blood into the first Hemocue cuvette. Clean up the excess of blood, place it into the Hemocue 301 and read (refer to Sections 4.3.3 Sample Preparation and 4.3.4 Acquisition and Analysis).
- Repeat procedure with a second cuvette and write instrument read-out.
    - If necessary apply gentle intermittent pressure along finger capillaries to allow them to refill with blood to help ensure continuous flow.
- Complete procedure. Wipe the site dry and apply direct pressure with a sterile gauze pad until the bleeding has stopped. Dispose of all used material following the facility's established procedures.

APPENDIX 11: ADULT INFORMED CONSENT FORM (CONSENT 04A)

This form covers the adult Male and Female (18 yrs or older) participants.

TITLE OF STUDY: Evaluation of the Investigational BD FACSPresto™ System: Instrument, Software, and BD CD4%CD4/Hb Cartridge Assay

BACKGROUND

You are being asked to take part in a medical research; research is also called a study. A medical study can look at what causes a disease as well as ways to **better identify, treat, prevent or control a disease. In this study, we will assess the performance of a new, lighter and easy to carry CD4 and Hemoglobin (Hb) testing devices (also referred to as CD4/Hb point-of-care tests) against the CD4 and Hb tests that are currently being used in hospitals and laboratories in Western Kenya. We will also find out the level of CD4 and Hb that normal healthy people have (also known as normal reference levels) and this would in future guide the grouping or classifying of CD4 and Hb results from the point-of-care testing devices as either high or low.** This study is from the U.S. Centers for Disease Control and Prevention (CDC), the Kenya Medical Research Institute (KEMRI), and the Kenya Ministry of Health (MOH).

In the study, 5 milliliters of blood (mL) will be collected from your arm (vein) and finger (via finger stick) and will be given an identity that is unique and different from the identity on your health/clinic records and sent to the KEMRI/CDC HIV-Research laboratory at the Jaramogi Oginga Odinga Teaching & Referral Hospital for testing.

Before you choose to be in this study, you need to know about any benefits or risks that could happen if you decide to join. This form tells you about the study and you can ask any questions you have at any time

BEING IN THE STUDY IS YOUR CHOICE

This information will be discussed with you. Once you know about the study and decide if you want to be in this study, you will be asked to sign this consent form and we will give you a copy to keep.

Before we tell you more about the study, you should know that:

- It is your choice to join.
- If you choose to be in the study, you can withdraw at any time.
- If you choose to be in the study, will be asked to respond to certain questions
- You may choose not to join the study.
- If you choose not to be in the study, you will still get your tests and the usual care.

<u>The goal of this study is to:</u>

4. **Asses the performance of the new, lighter and easy to carry CD4 and Hb testing device also known as the "BD FACSPresto™ System and CD4/%CD4/Hb cartridge" against that of the tests currently approved and being used in hospitals and laboratories to test for CD4 (called FACSCalibur flow cytometer) in blood collected from the veins (arms) and from finger pricks. We will place a unique number/code on the collected blood specimen so as to protect the participant's right to privacy and confidentiality. We will also find out the level of**

CD4 and Hb that normal healthy people have (also known as normal reference levels) and this would in future guide the grouping or classifying of CD4 and Hb results from the point-of-care testing devices as either high or low.

5. **Asses the performance of the new, lighter and easy to carry CD4 and Hb testing device against that of the tests currently approved and being used in hospitals and laboratories to test for Hemoglobin (Hb) levels.**

6. **To find out from those (customers) who conduct CD4 and Hb tests using the BD FACSPresto system how easy or challenging it is to do this.**

WHAT HAPPENS DURING THE STUDY

- When you join the study, a member of the study staff will ask you questions about your background, age, illness and medication history. Participation in the study is once.
- For the study, about one teasp**oo**n (5mL) of blood will be taken from a vein in your arm and from your finger when you join the study.
- As part of regular care, if your doctor requests blood tests to be done, the blood will be taken at the same time as the blood for the study test which will be sent to KEMRI/CDC HIV R Laboratory at the **Jaramogi Oginga Odinga Teaching & Referral Hospital (JOOTRH** formerly Nyanza Provincial Hospita **-** NPGH**)** for processing.
- Being in this study will take a little extra time (approximately 55 minutes) but your participation will only be required once.
- After each clinic visit we will look at your medical record. This is to help us learn about your illness and medication history. All the information will be kept private.
- All specimens in the study will be given an identity that is unique and different from the patient's personal health information by the assignment of a study-specific specimen ID.

IF YOU CHOOSE TO LEAVE THE STUDY

You can choose not to participate in this study. This will not affect the health care you get in the future.

RISKS TO YOU

There are minimal medical risks to you other than a possible mild discomfort or bruising as can be expected with normal venous and capillary (finger stick) blood collection procedures. Additionally, there is no risk of disclosure of confidential personal health information because the specimens will be uniquely identified upon enrollment in the study.

BENEFITS TO YOU

There are no direct benefits to you for participation in this study other than the opportunity to contribute to the performance evaluation of a new investigational Point of Care CD4/Hb testing device. No investigational results will be reported.

REASONS FOR TAKING YOU OUT OF THE STUDY WITHOUT YOUR CONSENT

You may be taken out of the study without your consent if:

- Your doctor decides that being in the study may cause harm to you;
- Your specimen is found to be unsuitable for testing i.e. if it has clotted
- The study is stopped by sponsor, KEMRI, or MOH;
- Other reasons beyond our control.

COSTS TO YOU

- There is no cost to you for participating in the study
- Upon participation in the study, you will receive a token of a bar of soap as appreciation and 300 Kenya shillings (approximately US$ 4) to cover estimated costs for travel.

YOUR RECORDS WILL BE PRIVATE

Your research records will be kept private as allowed by the law. Your study record will have a code number. Any information from your records will not be given out unless you agree in writing. Your name will not be in any reports about this study. People who review the study for KEMRI and the sponsor may need to look at your study records from time to time. For study needs, we will make copies of your medical records. All copies will be kept in a locked file cabinet. Only study staff will have access to this cabinet.

BLOOD SAMPLES

Your blood samples or specimen will be stored in Kenya, at the KEMRI/CDC laboratories, for the duration of the study. They will be destroyed once the study is completely over.

PROBLEMS OR QUESTIONS

For questions about this study or a research-related injury, contact:

- Frank Angira at the KEMRI/CDC study clinic at Jaramogi Oginga Odinga Teaching & Refferral Hospital (JOOTRH) off Kakamega Road, Kisumu (P.O. Box 1578, Kisumu) or phone number: 057-2022929 Ext 619, Email: fangira@kemricdc.org

- Dr. **John Vulule Principal Investigator and** Director KEMRI/CDC Field Station, KEMRI, Center for Global Health Research, off Kisumu-Busia Road, Kisumu) Tel: 0572022983/2022959/2022902 KEMRI/CDC, P.O. Box 1578, Kisumu, Kenya

For questions about your rights as a research subject, contact:

The Secretary, **KEMRI Ethics Review Committee**
P.O. Box 54840-00200, Nairobi, Kenya
Telephone: 020-2722541, 0722 205901, 0733 400003
Email Address : **ERCadmin@kemri.orgt**

Principal Investigator: Clement Zeh
Co-investigators: Lisa A. Mills, Imelda O. Zapata, Frank Angira, Benta Akoth, Boaz Oyaro, Oscar Segurado, Lorlelei Lee-Haynes, Jerry Zhang, Jeannine Paliotta, Henok Tilahun, Kayla Laserson, John Vulule

STATEMENT OF CONSENT:

Please sign your name below once you:

9. have read (have been explained) and know the reasons for the study,
10. know the steps to be followed in the study,
11. know the risks and benefits to you from being in the study,
12. AND choose to enroll in this study of your own free will.

**Volunteer's Name**<br>**Type or print**	***Volunteer's Signature***<br>(Volunteer does **NOT** sign here if she is under age 18)	**Date**

**Witness's Name**<br>**Type or print**	**Witness's Signature**	**Date**

I have explained the purpose of this study to the volunteer. To the best of my knowledge, she understands the purpose, procedures, risks and benefits of this study.

**Investigator/Designee Name**<br>***Type or print***	**Investigator/Designee Signature**	**Date**

NOTE: This consent form with original signatures must be retained on file by the principal investigator. A copy must be given to the volunteer. A copy should be placed in the volunteer's medical record (if applicable).

APPENDIX 12: PARENTAL/GUARDIAN CONSENT FORM (CONSENT 05A)

This form covers the male and female children.

TITLE OF STUDY: Evaluation of the Investigational BD FACSPresto™ System: Instrument, Software, and BD CD4%CD4/Hb Cartridge Assay

BACKGROUND

You are being asked to allow your child to take part in a medical research; research is also called a study. A medical study can look at what causes a disease as well as ways to better **identify, treat, prevent or control a disease. In this study, we will assess the performance of a new, lighter and easy to carry CD4 and Hemoglobin (Hb) testing devices (also referred to as CD4/Hb point-of-care tests) against the CD4 and Hb tests that are currently being used in hospitals and laboratories in Western Kenya. We will also find out the level of CD4 and Hb that normal healthy people have (also known as normal reference levels) and this would in future guide the grouping or classifying of CD4 and Hb results from the point-of-care testing devices as either high or low.** This study is from the U.S. Centers for Disease Control and Prevention (CDC), the Kenya Medical Research Institute (KEMRI), and the Kenya Ministry of Health (MOH).

In the study, 5 milliliters of blood will be collected from your child's arm (vein) and finger (via finger stick) and will be given an identity that is unique and different from the identity on his/her health/clinic records and sent to the KEMRI/CDC HIV-Research laboratory at the Jaramogi Oginga Odinga Teaching & Referral l Hospital for testing.

Before you allow your child to be in this study, you need to know about any benefits or risks that t could happen if you decide that your child joins the study. This form tells you about the study and you can ask any questions you have at any time

BEING IN THE STUDY IS YOUR CHOICE

This information will be discussed with you. Once you know about the study and decide if you want your child to be in this study, you will be asked to sign this consent form and we will give you a copy to keep.

Before we tell you more about the study, you should know that:

- It is your choice to allow your child to be in the study.
- If you choose to allow your child to be in the study, you can withdraw him/her at any time.
- If you choose to allow your child to be in the study, will be asked to respond to certain questions.
- You may choose not to allow your child to join the study.
- If you choose not to allow your child to be in the study, your child will still get his/her tests and the usual care.

<u>The goal of this study is to:</u>

1. **Asses the performance of the new, lighter and easy to carry CD4 and Hb testing device also known as the "BD FACSPresto™ System and CD4/%CD4/Hb cartridge" against that of the tests currently approved and being used in hospitals and laboratories to test for CD4 (called FACSCalibur flow cytometer) in blood collected from the veins (arms) and from finger pricks. We will place a unique number/code on the collected blood specimen so as to protect the**

participant's right to privacy and confidentiality. We will also find out the level of CD4 and Hb that normal healthy people have (also known as normal reference levels) and this would in future guide the grouping or classifying of CD4 and Hb results from the point-of-care testing devices as either high or low.

2. **Asses the performance of the new, lighter and easy to carry CD4 and Hb testing device against that of the tests currently approved and being used in hospitals and laboratories to test for Hemoglobin (Hb) levels.**

3. **To find out from those (customers) who conduct CD4 and Hb tests using the BD FACSPresto system how easy or challenging it is to do this.**

WHAT HAPPENS DURING THE STUDY

- When your child joins the study, a member of the study staff will ask you questions about your child's background, age, illness and medication history. Participation in the study is once.
- For the study, less than a teaspoon (5ml) of blood will be taken from a vein in your child's arm and from his/her finger when he/she joins the study.
- As part of regular care if your child's doctor requests blood tests to be done, the blood will be taken at the same time as the blood for the study test which will be sent to KEMRI/CDC HIV R Laboratory at the **Jaramogi Oginga Odinga Teaching & Referral Hospital (JOOTRH** formerly Nyanza Provincial Hospital) for processing.
- Being in this study will take a little extra time (approximately 55 minutes) but your participation will only be required once.
- After each clinic visit we will look at your child's medical record. This is to help us learn about your child's illness and medication history. All the information will be kept private.
- All specimens in the study will be given an identity that is unique and different from the one in the patient's personal health information by the assignment of a study-specific specimen ID.

IF YOU CHOOSE TO TAKE YOUR CHILD FROM THE STUDY

You can choose not to participate in this study. This will not affect the health care you get in the future.

RISKS TO YOUR CHILD

There are minimal medical risks to your child other than a possible mild discomfort or bruising as can be expected with normal venous and capillary (finger stick) blood collection procedures. Additionally, there is no risk of disclosure of confidential personal health information because the specimens will be **uniquely identified** upon enrollment in the study.

BENEFITS TO YOUR CHILD

There are no direct benefits to your child for participation in this study other than the opportunity to contribute to the performance evaluation of a new investigational Point of care CD4/Hb testing device. No investigational results will be reported.

REASONS FOR TAKING YOUR CHILD OUT OF THE STUDY WITHOUT YOUR CONSENT

Your child may be taken out of the study without your consent if:

- Your child's doctor decides that being in the study may cause harm to him/her;
- Your child's specimen is found to be unsuitable for testing i.e. if it has clotted.
- The study is stopped by sponsor, KEMRI, or MOH;

- Other reasons beyond our control.

COSTS TO YOUR CHILD

- There is no cost to you for participating in the study
- Upon participation in the study, you/your child will receive a token of a bar of soap as appreciation and 300 Kenya shillings (approximately US$ 4) to cover estimated costs for travel.

YOUR CHILD'S RECORDS WILL BE PRIVATE

Your child's research records will be kept private as allowed by the law. Your child's study records will have a code number. Any information from your child's records will not be given out unless you agree in writing. Your child's name will not be in any reports about this study. People who review the study for KEMRI and the sponsor may need to look at your child's study records from time to time. For study needs, we will make copies of your child's medical records. All copies will be kept in a locked file cabinet. Only study staff will have access to this cabinet.

BLOOD SAMPLES

Your child's blood samples or specimen will be stored in Kenya, at the KEMRI/CDC HIV-Research laboratories, for the duration of the study. They will be destroyed once the study is completely over.

PROBLEMS OR QUESTIONS

For questions about this study or a research-related injury, contact:

- Frank Angira at the KEMRI/CDC study clinic at Jaramogi Oginga Odinga Teaching & Refferral Hospital (JOOTRH) off Kakamega Road, Kisumu (P.O. Box 1578, Kisumu) or phone number: 057-2022929 Ext 619, Email: fangira@kemricdc.org
-
- Dr. **John Vulule Principal Investigator and** Director KEMRI/CDC Field Station, KEMRI, Center for Global Health Research, off Kisumu-Busia Road, Kisumu) Tel: 0572022983/2022959/2022902 KEMRI/CDC, P.O. Box 1578, Kisumu, Kenya

For questions about your rights as a research subject, contact:

The Secretary, **KEMRI Ethics Review Committee**
P.O. Box 54840-00200, Nairobi, Kenya
Telephone: 020-2722541, 0722 205901, 0733 400003
Email Address : **ERCadmin@kemri.orgt**

Principal Investigator: Clement Zeh
Co-investigators: Lisa A. Mills, Imelda O. Zapata, Frank Angira, Benta Akoth, Boaz Oyaro, Oscar Segurado, Lorlelei Lee-Haynes, Jerry Zhang, Jeannine Paliotta, Henok Tilahun, Kayla Laserson, John Vulule

STATEMENT OF CONSENT:

Please sign your name below once you:

5. have read (have been explained) and know the reasons for the study,
6. know the steps to be followed in the study,
7. know the risks and benefits to your child for being in the study,
8. AND choose to enroll your child in this study of your own free will.

**Child's Name**<br>**Type or print**		
**Name of Child's Parent or Legal Guardian**<br>(must be over 18 years old)<br>***Type or print***	**Legal Guardian's Signature**	**Date**
**Witness's Name**<br>***Type or print***	**Witness's Signature**	**Date**

I have explained the purpose of this study to the volunteer. To the best of my knowledge, he/she understands the purpose, procedures, risks and benefits of this study.

**Investigator/Designee Name**<br>***Type or print***	**Investigator/Designee Signature**	**Date**

NOTE: This consent form with original signatures must be retained on file by the principal investigator. A copy must be given to the volunteer. A copy should be placed in the volunteer's medical record (if applicable).

<u>APPENDIX 13: MINOR ASSENT FORM (ASSENT 06A)</u>

This form covers Male and Female study participants aged between 13 and 17 years. A legal guardian must sign the consent form.

TITLE OF STUDY: Evaluation of the Investigational BD FACSPresto™ System: Instrument, Software, and BD CD4%CD4/Hb Cartridge Assay

BACKGROUND

You are being asked to take part in a medical research; research is also called a study. A medical study can look at what causes a disease as well as ways to better **identify, treat, prevent or control a disease. In this study, we will assess the performance of a new, lighter and easy to carry CD4 and Hemoglobin (Hb) testing devices (also referred to as CD4/Hb point-of-care tests) against the CD4 and Hb tests that are currently being used in hospitals and laboratories in Western Kenya. We will also find out the level of CD4 and Hb that normal healthy people have (also known as normal reference levels) and this would in future guide the grouping or classifying of CD4 and Hb results from the point-of-care testing devices as either high or low.** This study is from the U.S. Centers for Disease Control and Prevention (CDC), the Kenya Medical Research Institute (KEMRI), and the Kenya Ministry of Health (MOH).

In the study, 5 milliliters of blood (mL) will be collected from your arm (vein) and finger (via finger stick) and will be given an identity that is unique and different from the identity on your health/clinic records and sent to the KEMRI/CDC HIV-Research laboratory at the Jaramogi Oginga Odinga Teaching & Referral Hospital for testing.

Before you choose to be in this study, you need to know about any benefits or risks that could happen if you decide to join. This form tells you about the study and you can ask any questions you have at any time

BEING IN THE STUDY IS YOUR CHOICE

This information will be discussed with you. Once you know about the study and decide if you want to be in this study, you will be asked to sign this consent form and we will give you a copy to keep.

Before we tell you more about the study, you should know that:

- It is your choice to join.
- If you choose to be in the study, you can withdraw at any time.
- If you choose to be in the study, you will be asked to respond to certain questions
- You may choose not to join the study.
- If you choose not to be in the study, you will still get your tests and the usual care.

<u>The goal of this study is to:</u>

1. **Asses the performance of the new, lighter and easy to carry CD4 and Hb testing device also known as the "BD FACSPresto™ System and CD4/%CD4/Hb cartridge" against that of the tests currently approved and being used in hospitals and laboratories to test for CD4 (called FACSCalibur flow cytometer) in blood collected from the veins (arms) and from finger pricks. We will place a unique number/code on the collected blood specimen so as to protect the**

participant's right to privacy and confidentiality. We will also find out the level of CD4 and Hb that normal healthy people have (also known as normal reference levels) and this would in future guide the grouping or classifying of CD4 and Hb results from the point-of-care testing devices as either high or low.

2. **Asses the performance of the new, lighter and easy to carry CD4 and Hb testing device against that of the tests currently approved and being used in hospitals and laboratories to test for Hemoglobin (Hb) levels.**

3. **To find out from those (customers) who conduct CD4 and Hb tests using the BD FACSPresto system how easy or challenging it is to do this.**

WHAT HAPPENS DURING THE STUDY

- When you join the study, a member of the study staff will ask you questions about your background, age, illness and medication history. Participation in the study is once.
- For the study, less than a teaspoon (5mL) of blood will be taken from a vein in your arm and from your finger when you join the study.
- As part of regular care if your doctor requests blood tests to be done, the blood will be taken at the same time as the blood for the study test which will be sent to KEMRI/CDC HIV-Research Laboratory at the **Jaramogi Oginga Odinga Teaching & Referral Hospital (JOOTRH** formerly Nyanza Provincial Hospital) for processing.
- During this study, you will continue to visit the clinic for your regular care. Being in this study will take a little extra time (approximately 55 minutes) but your participation will only be required once.
- After each clinic visit we will look at your medical record. This is to help us learn about your illness and medication history. All the information will be kept private.
- All specimens in the study will be given an identity that is unique and different from the one in your personal health information by the assignment of a study-specific specimen ID.

IF YOU CHOOSE TO LEAVE THE STUDY

You can choose not to participate in this study. This will not affect the health care you get in the future.

RISKS TO YOU

There are minimal medical risks to you other than a possible mild discomfort or bruising as can be expected with normal venous and capillary (finger stick) blood collection procedures. Additionally, there is no risk of disclosure of confidential personal health information because the specimens will be uniquely identified upon enrollment in the study.

BENEFITS TO YOU

There are no direct benefits to you for participation in this study other than the opportunity to contribute to the performance evaluation of a new investigational Point of Care CD4/Hb testing device. No investigational results will be reported.

REASONS FOR TAKING YOU OUT OF THE STUDY WITHOUT YOUR ASSENT

You may be taken out of the study without your assent if:

- Your doctor decides that being in the study may cause harm to you;
- Your specimen is found to be unsuitable for testing i.e. if it has clotted.

- The study is stopped by sponsor, KEMRI, or MOH;
- Other reasons beyond our control.

COSTS TO YOU

- There is no cost to you for participating in the study
- Upon participation in the study, you will receive a token of a bar of soap as appreciation and 300 Kenya shillings (approximately US$ 4) to cover estimated costs for travel.

YOUR RECORDS WILL BE PRIVATE

Your research records will be kept private as allowed by the law. Your study record will have a code number. Any information from your records will not be given out unless you agree in writing. Your name will not be in any reports about this study. People who review the study for KEMRI and the sponsor may need to look at your study records from time to time. For study needs, we will make copies of your medical records. All copies will be kept in a locked file cabinet. Only study staff will have access to this cabinet.

BLOOD SAMPLES

Your blood samples or specimen will be stored in Kenya, at the KEMRI/CDC laboratories, for the duration of the study. They will be destroyed once the study is completely over.

PROBLEMS OR QUESTIONS

For questions about this study or a research-related injury, contact:

- Frank Angira at the KEMRI/CDC study clinic at Jaramogi Oginga Odinga Teaching & Refferral Hospital (JOOTRH) off Kakamega Road, Kisumu (P.O. Box 1578, Kisumu) or phone number: 057-2022929 Ext 619, Email: fangira@kemricdc.org

- Dr. **John Vulule Principal Investigator and** Director KEMRI/CDC Field Station, KEMRI, Center for Global Health Research, off Kisumu-Busia Road, Kisumu) Tel: 0572022983/2022959/2022902 KEMRI/CDC, P.O. Box 1578, Kisumu, Kenya

For questions about your rights as a research subject, contact:

The Secretary, **KEMRI Ethics Review Committee**
P.O. Box 54840-00200, Nairobi, Kenya
Telephone: 020-2722541, 0722 205901, 0733 400003
Email Address : **ERCadmin@kemri.org**

Principal Investigator: Clement Zeh
Co-investigators: Lisa A. Mills, Imelda O. Zapata, Frank Angira, Benta Akoth, Boaz Oyaro, Oscar Segurado, Lorlelei Lee-Haynes, Jerry Zhang, Jeannine Paliotta, Henok Tilahun, Kayla Laserson, John Vulule

STATEMENT OF ASSENT:

Please sign your name below once you:

13. have read (have been explained) and know the reasons for the study,
14. know the steps to be followed in the study,
15. know the risks and benefits to you from being in the study,
16. AND choose to enroll in this study of your own free will.

______________________	______________________	______________________
**Minor's name**<br>**Type or print**	**Minor's signature**	**Date**

______________________	______________________	______________________
**Witness's Name**<br>**Type or print**	**Witness's Signature**	**Date**

I have explained the purpose of this study to the volunteer. To the best of my knowledge, she understands the purpose, procedures, risks and benefits of this study.

______________________	______________________	______________________
**Investigator/Designee Name**<br>***Type or print***	**Investigator/Designee Signature**	**Date**

NOTE: This assent form with original signatures must be retained on file by the principal investigator. A copy must be given to the volunteer. A copy should be placed in the volunteer's medical record (if applicable).
