# Supplementary material for: Clinical Evaluation of the BD FACSPresto™ Near-Patient CD4 Counter in Kenya
Source: PLoS One. 2016 Aug 2;11(8):e0157939. doi: 10.1371/journal.pone.0157939 (PMC4970792; doi:10.1371/journal.pone.0157939)
Supplement: S2 File — (PDF) [file pone.0157939.s003.pdf]

Clinical Study Protocol

Precision Evaluation of the Investigational BD FACSPresto™ System: Instrument, Software, and BD CD4/%CD4/Hb Cartridge

**Protocol Number:**	CAS-PCPRE
**Version:**	1.0
**Date:**	29/JUL/2013

**Principal Investigator**	Laurie Byrne<br>Medical Laboratory Manager<br>Phone: (408) 954-6069<br>Fax : (408) 954-6313<br>Email: Laurie_Byrne@bd.com
**Study Sites**	BD Biosciences, MEDLab<br>2350 Qume Drive<br>San Jose, CA 95131
**Sponsor**	BD Biosciences<br>2350 Qume Drive<br>San Jose, CA 95131

For Investigational Use Only

The Performance characteristics of this product have not been established.

All investigational material supplied by BD Biosciences must be used for study purposes only. Results from investigational testing must not be used as a diagnostic procedure without confirmation of the diagnosis by another medically established diagnostic product or procedure.

Confidential

The product information and data disclosed through this study protocol are confidential and are subject to a Confidential Disclosure Agreement. No content may be disclosed without prior written consent of Becton, Dickinson and Company.

SPONSOR

BD Biosciences
2350 Qume Drive
San Jose, CA 95131

Medical Affairs	*Kevin Judge, MD*<br>*VP of Medical Affairs and Medical Monitor*<br>Phone: 201-847-4454<br>Email: kevin_judge@bd.com
Clinical Operations	*Lorlelei Lee-Haynes, MPH*<br>*Director of Clinical Operations*<br>Phone: 408-954-6309<br>Fax: 408-954-6313<br>Email: lorlelei-lee_haynes@bd.com
CPL / Study Manager	*Imelda Omana-Zapata, MD, PhD*<br>*Clinical Project Lead*<br>Phone: 408-954-2323<br>Fax: 408-954-6313<br>Email: imelda_omana-zapata@bd.com
CRA / Study Monitor	*Maryam Saleminik*<br>*Clinical Research Associate*<br>Phone: 408-518-5037<br>Fax: 408-954-6313<br>Email: MSaleminik@bd.com
Biostatistics	*Beverly Lu*<br>*Biostatistician*<br>Phone: 408-954-6301<br>Email: beverly_lu@bd.com
Data Management	*Jeannine Paliotta*<br>*Data Manager*<br>Phone: 408-954-4111<br>Email: Jeannine_Paliotta@bd.com

TABLE OF CONTENTS

1. INTRODUCTION **7**
1.1. Statement of Compliance *7*
1.2. Background Information and Scientific Rationale *7*
1.3. Proposed Intended Use *7*

2. OBJECTIVES **7**
2.1. Primary Objective(s) *7*
2.1.1. Repeatability Procedure 7
2.1.2. Precision Procedure 8
2.2. Secondary Objective(s) *8*
2.2.1. Repeatability Procedure 8
2.2.2. Precision Procedure 8

3. STUDY DESIGN **8**
3.1. Overall Study Design / Outcome Measures *8*
3.2. Expected Study Duration *8*
3.3. Study Device(s) *8*
3.3.1. Investigational Device(s) 8
3.3.2. Predicate Device 9

4. STUDY POPULATION **9**
4.1. Specimen Requirements *9*
4.2. Inclusion Criteria *9*
4.3. Exclusion Criteria *9*
4.4. Sample Size *9*

5. STUDY METHODS/PROCEDURES **9**
5.1. Daily Study Activities *9*
5.1.1. Instrument Setup/ Instrument QC 10
5.1.2. Process Controls 10
5.2. Specimen Enrollment *10*
5.3. Enrolled Specimen Preparation *10*
5.3.1. Repeatability Procedure 10
5.3.2. Precision Procedure 10
5.3.3. Enrolled Sample Acquisition and Analysis 11
5.3.4. Acquisition Schedule for Precision Procedure 11

6. DISCONTINUATION OF SPECIMEN TESTING **12**

7. PRODUCT ERRORS/ DEFECTS/ FAILURES **13**

8. DATA COLLECTION AND MANAGEMENT **13**
8.1. Data Collection and Source Documents *13*
8.2. Submission of Data to Sponsor *13*

8.2.1. Data Packages 13
8.3. Data Evaluation for Inclusion/Exclusion from Analysis 13
8.4. Data Management and Storage 14

9. STATISTICAL METHODS 14
9.1. Sample Size Determination 14
9.2. Eligibility, Exclusions, Missing Data, Interim Analysis 14
9.3. General Statistical Considerations 15
9.4. Study Endpoints / Outcome Measures 15
9.4.1. Primary endpoint 15
Repeatability Procedure 15
9.4.2. Precision Procedure 15
9.4.3. Secondary Endpoints 15
Repeatability Procedure 15
Precision Procedure 15
9.5. Acceptance Criteria 15
9.6. Methods to Reduce Bias 16
9.7. Analysis Method 16
9.7.1. Statistical Quality Control Chart 16
9.7.2. Outlier Check 16
9.8. Biostatistics Report 16

10. STUDY MATERIALS AND SUPPLIES 17
10.1. Investigational Product Labeling 17
10.2. Study Products Shipment and Receipt 18
10.3. Study Product Accountability 17
10.4. Required Study Materials and Supplies 17
10.4.1. Instrumentation 17
10.4.2. Reagents, and Controls 17
10.4.3. General Product Use and Study-Specific Documents 18
10.4.4. Ancillary Items 18
10.5. Disposition 19
10.5.1. Reagents 19

11. MONITORING 19
11.1.1. Study Monitors 19
11.2. Monitoring Procedures 19
11.2.1. Case Report Forms (CRFs) Management 19
11.2.2. Monitoring Procedure and Documentation 19
11.3. Audits/Inspections 19

12. SAFETY REPORTING 19

13. STUDY SUSPENSION OR TERMINATION 20

14. STUDY ETHICS/ GOOD PRACTICES 20
14.1. Ethics Review and Approval 20
14.2. Informed Consent 20
14.3. Risks and Benefits 20
14.4. Donor Identification and Confidentiality 20
14.5. Donor Remuneration 20

15. INVESTIGATOR 20
15.1. Clinical Investigators 20
15.2. Submission and obtaining Ethics Approval 20
15.2.1. Source Documents 21
15.2.2. Device Accountability 21
15.2.3. Data Recording and Record Retention 21
15.3. Investigator Reports 21
15.3.1. Withdrawal of EC/IRB Approval 21
15.3.2. Deviation from the Study Protocol 21
15.3.3. Use of Device without Informed Consent (applicable if Informed Consent is required) 21
15.3.4. Final Report 21
15.3.5. Other Reports 21
15.4. Responsibility for Investigator Reports 22
15.5. Financial Disclosure 22

16. SPONSOR 22
16.1. Sponsor Staff Qualifications 22
16.2. Beginning the Study 22
16.3. Selecting Investigators 22
16.4. Ensuring Proper Monitoring 22
16.5. Ensuring Proper Notification 22
16.6. Control of Device 22
16.7. Obtaining Agreements 22
16.8. Securing Compliance 23
16.9. Resumption of Suspended or Terminated Studies 23
16.10. Reporting 23
16.11. Records 23

17. PUBLICATION 23

18. REFRENCES 23

19. CHANGE HISTORY 24

LIST OF ABBREVIATIONS AND DEFINITION OF TERMS

%CD4	Percentage of $CD4^+$ T lymphocytes of total lymphocytes
AbsCD4	Absolute cell count (cells/μL) of all T lymphocytes that are $CD4^+$
AIDS	Acquired Immunodeficiency Syndrome
ART	Antiretroviral therapy
BD	Becton Dickinson and Company. BD Biosciences, hereafter referred to as sponsor
CBC	Complete blood counts
CD4	Antigen present on the “helper/inducer” T lymphocyte subpopulation
CI	Confidence Interval
CRA	Clinical Research Associate
CRF	Case Report Form
DCF	Data Clarification Form
FDA	Food and Drug Administration
GCP	Good Clinical Practice
HIV	Human Immunodeficiency Virus
ICH	International Conference on Harmonization for Registration of Pharmaceuticals for Human Use; as adopted by the FDA (i.e., Good Clinical Practice E6)
ICF	Informed Consent Form
EC/IRB	An Institutional Review Board or Independent Ethics Committee
IUO	Investigational Use Only
IVD medical device	Any medical device which is a reagent, reagent product, calibrator, control material, kit, instrument, apparatus, equipment, or system, whether used alone or in combination, intended by the manufacturer to be used *in vitro* for the examination of specimens, including blood and tissue donations, derived from the human body, solely or principally for the purpose of providing information: concerning a physiological or pathological state, or concerning a congenital abnormality, or to determine the safety and compatibility with potential recipients, or to monitor therapeutic measures.**
Predicate or Predicate Method	The previously approved or generally accepted method
PI	Principal Investigator
QC	Quality Control
UADE	Unanticipated Adverse Device Effect
WB	Whole Blood
WBC	White cell blood counts
WHO	World Health Organization

** 98/79/EC:1998, Directive on *in vitro* diagnostic medical devices

1. INTRODUCTION

1.1. Statement of Compliance

The study is to be conducted in compliance with this protocol, Good Clinical Practice (GCP)[1], the Declaration of Helsinki, Title 21 of the Code of Federal Regulations §§ 50, 56, and 812, International Conference on Harmonization E6 and ISO Standards 14155:2011.

1.2. Background Information and Scientific Rationale

The enumeration of T lymphocytes that are positive for the CD4 antigen is used to evaluate the immune status of patients with, or suspected of developing, immune deficiencies such as AIDS. The CD4 antigen is the receptor for Human Immunodeficiency Virus (HIV) and the number of CD4+ T lymphocytes declines due to programmed cell death in an HIV infection[2]. The absolute count of CD4+ T lymphocytes (Abs CD4) and the percentage of CD4+ T lymphocytes of total lymphocytes (%CD4) are cellular parameters closely associated with HIV disease progression and patient prognosis. According to World Health Organization (WHO) guidance documents regarding HIV/AIDS patient treatment, the measurement of Abs CD4 cell counts and %CD4+ in adolescents and adults[3,4] is recommended for decision-making on initiating antiretroviral therapy (ART). Recently, the use of ART has been extended on prevention or prophylaxis.[5,6]

Anemia can be induced by ART,[7] or can be one of the hematological abnormalities frequently observed in HIV/AIDS patients in developing countries[8]. Anemia is usually diagnosed by measuring the concentration of hemoglobin (Hb) and by counting the number of red cells or hematocrit in whole blood. It is common practice during the patient's routine visit to evaluate: the presence of anemia, other hematological or functional parameters to assess HIV/AIDS disease progression or control, and adverse events induced by ART use.

The investigational-use only BD FACSPresto™ System (BD FACSPresto instrument with embedded software and BD CD4/%CD4/Hb cartridge or IUO BD cartridge) is designed to provide enumeration of CD4+ absolute counts (cells/μL) as well as the percentage $CD4^+$ T lymphocytes of the total lymphocyte count (%CD4) and Hb concentration in g/dL or g/L. Peripheral whole blood and commercialized process controls can be analyzed with this instrument.

The BD CD4/%CD4/Hb cartridge contains dried fluorochrome-conjugated antibody reagent. When whole blood reacts with the reagents, the antibodies in the reagents bind to the surface antigens on the lymphocytes and monocytes. Samples are incubated for eighteen minutes or less than two hours; then after incubation the sample is run on the instrument. The software identifies the cell populations of interst and calculates CD4 absolute counts, CD4 percentages of lymphocytes, and hemeglobin concentration.

This study is designed to confirm that the investigational system (BD FACSPresto™ instrument with embedded software and BD™ CD4/%CD4/Hb cartridge) meets design input requirements for repeatability and precision.

1.3. Proposed Intended Use

The BD FACSPresto™ is an automated system for *in vitro* diagnostic use in performing the direct enumeration of CD4 absolute count, CD4 percentage of lymphocytes, and Hb concentration in human whole blood.

2. OBJECTIVES

2.1. Primary Objective(s)

2.1.1. Repeatability Procedure

The primary objective of the Repeatability evaluation is to demonstrate that the AbsCD4+ and %CD4+ of lymphocytes in the specimen samples meet the repeatability Endpoints and Acceptance Criteria outlined in Sections 9.4 and 9.5 of this protocol.

2.1.2. Precision Procedure

The primary objective of the precision evaluation is to demonstrate that the absolute CD4+ cell counts and CD4+ as a percentage of lymphocytes in the specimen samples meet the total precision specifications outlined in the Design Input Document (DID) as listed in Section 9.5 of this protocol.

2.2. Secondary Objective(s)

2.2.1. Repeatability Procedure

The secondary objective of the Repeatability evaluation is to demonstrate that the Hb concentration in the specimen samples meet repeatability Endpoints and Acceptance Criteria outlined in Section 9.5 of this protocol.

2.2.2. Precision Procedure

The secondary objective of the precision evaluation is to demonstrate that the Hb concentration in the specimen samples meet the total precision specifications outlined in the Design Input Document (DID) as listed in Section 9.5 of this protocol.

3. STUDY DESIGN

3.1. Overall Study Design / Outcome Measures

The design and proposed analysis for both the Repeatability and Precision procedures follow recommendations given in CLSI Guideline EP5-A2: Evaluation of Precision Performance of Quantitative Measurement Methods[9].

Repeatability will be measured by staining twenty replicates of appropriate test materials (i.e. two concentration levels of process controls) using one lot of IUO BD CD4/%CD4/Hb cartridge by one operator and acquiring on one FACSPresto instrument in one day.

Precision will measure operator and instrument variability, across three FACSPresto instruments handled by three operators who will use three different lots of IUO BD CD4/%CD4/Hb cartridges. Precision measurements will be taken in two separate runs per day (i.e., morning and afternoon) using one instrument and one cartridge lot per day, over a period of 21 days. A set of process control will be stained in singlicate for each run prior testing of Precision samples. Precision samples will consist of CD4 and Hb process controls that will be stained in duplicate and acquired in each run. The two runs will be separated by a minimum of 2 hours. This period is defined as the time between the acquisition time for the last cartridge in the first run and the acquisition time for the first process control cartridge in the second run.

BD Biostatistician will provide a rotation schedule for instruments, operators and cartridge lots as part of the Workflow Notes Instruction.

3.2. Expected Study Duration

The anticipated study duration for Repeatability procedure is two days (1 day for AbsCD4+ count and %CD4 measurment and 1 day for HB measurment) and for Precision is 4-6 weeks (includes 21 working days plus supplemental data collection, if needed).

3.3. Study Device(s)

3.3.1. Investigational Device(s)

The investigational devices for this study are the IUO BD FACSPresto instrument with embedded software and IUO BD CD4/%CD4/Hb cartridge that will provide the absolute count of CD4+ T lymphocyte cells/μL of blood (Abs CD4), the percentage of CD4-positive T lymphocytes of total lymphocytes (%CD4) and Hb concentration in g/dL or g/L.

3.3.2. Predicate Device

Not applicable for this study.

4. STUDY POPULATION

4.1. Specimen Requirements

All protocol testing activities will be conducted at BD Biosciences MEDLab using Streck CD Chex Plus and Eurotrol controls as samples. As process controls, two levels of BD Multi checks (normal and low) will be used to measure the AbsCD4 and the %CD4 and 3 levels of Eurotrol Controls (Level 1, 2, 3) to measure the Hb concentration.

To eliminate sample variation, one lot of controls (Streck and Eurotrol) will be used as the test samples for this study.

For the repeatability procedure two levels of Streck CD Chex Plus control (normal and low) controls will be used to measure the AbsCD4 and the %CD4 and 2 levels of Eurotrol Controls (Level 1and 2) will be used as samples to measure the Hb concentration.

For the precision procedure two levels of Streck CD Chex Plus control (normal and low) controls will be used to measure the AbsCD4 and the %CD4 and 3 levels of Eurotrol Controls (Level 1, 2, 3) will be used as the precision samples to measure the Hb concentration.

4.2. Inclusion Criteria

For inclusion, controls must be within expiration date.

4.3. Exclusion Criteria

Controls that exhibit precipitation and/or variation in viscosity, or present other visually observable problems should be not be used and the vials discarded.

4.4. Sample Size

The sample size for the Repeatability and Precision procedures is based on CLSI guidance document EP5-A2.

For Repeatability procedure twenty replicates of test materials (two concentration levels) are required to run on one instrument by one operator in one day (one day for AbsCD4+ count and %CD4 measurment and one day for HB measurment).

For Precision precedure forty-two (42) runs (2 runs per day times 21 days) are required. Each run will consist of duplicate values for the each precision sample (Streck CD Chex normal and low, and level 1, 2, 3 of Eurotrol) and a single value for each process control. Additional runs may be conducted in excess of those noted in anticipation of non-evaluable data. The EP5-A2 guidance document recommends testing two concentrations of test material and one quality control sample per run.

5. STUDY METHODS/PROCEDURES

5.1. Daily Study Activities

Daily study activities include the following:

- FACSPresto QC and Process Controls
- Specimen Enrollment
- Sample Preparation and Acquisition
- Data review and completion of case Report Forms and applicable study logs

5.1.1. Instrument Setup/ Instrument QC

On each day of study testing, instrument QC must be performed on the investigational BD FACSPresto system and Passing results must be obtained prior to running any process controls or precision samples. Instrument setup will be done once daily. Instrument QC/setup automatically runs every time the instrument turns on.

5.1.2. Process Controls

On each day of study testing, a separate set of process controls must be stained in singlicate per run. For AbsCD4 and %CD4, BD Multi-Check control normal and CD4Low will be used as process control and for Hb concentration; Eurotrol 301 (level 1, 2, and 3) will be used as process control per following steps:

- Label one BD CD4/%CD4/Hb cartridge for each process control (Multi-Check normal and CD4 low and Eurotrol level 1, 2, and 3) as instructed in the naming convention in the workflow notes instructions. (total of 5 cartridges)
- Prepare Process Controls per their package insert and add 20-25µL well mixed process control to the well of each labeled cartridge using transfer pipette.
- Incubate cartridges for a minimum of 18 minutes at room temprature on the incubation tray.
- Enter the process control information using the touch screen as instructed in the workflow note instructions.
- Once incubation is finished, load the IUO BD cartridge into the instrument and start the run.
- Print specimen results and verify that results (i.e., AbsCD4, %CD4, and Hb concentration) fall within the manufacturer's lot-specific ranges.

5.2. Specimen Enrollment

On each day of study testing, study personnel will verify precision sample material has been stored according to the manufacturer's specifications and meets enrollment criteria.

5.3. Enrolled Specimen Preparation

5.3.1. Repeatability Procedure

- Label twenty IUO BD CD4/%CD4/Hb cartridges for each level of samples (Streck CD Chex plus normal and low and Eurotrol (level 1 and 2) as instructed in the naming convention indicated in the Workflow Notes instructions.
- Invert the specimen ten times, and then add 20-25µL well mixed sample to the well of each labeled cartridge using a transfer pipette.
- Record "Specimen addition time" on the "sample preparation" CRF.
- Incubate the cartridges at room temperature (i.e., 20 to 25 ºC) on the incubation tray for at least 18 minutes and acquire samples within 2 hours from the time of blood addition onto the cartridges.

5.3.2. Precision Procedure

Prepare Streck CD Chex plus and Eurotrol samples per package insert and stain and acquire on the investigational system as follows. Refer to the IUO BD FACSPresto Reagent kit "IUO" instructions for use for detailed sample preparation instructions.

- Label two BD IUO CD4/%CD4/Hb cartridges for each level of precision sample (Streck CD Chex plus normal and low and Eurotrol (level 1, 2, and 3) as instructed in the naming convention in the workflow notes instructions. (total of 10 cartridges)
- Invert the control sample ten times and using a transfer pipette add 20-25µL well mixed control sample to the well of each labeled cartridge.
- Record “Specimen addition time” on the “sample preparation” CRF.
- Incubate the cartridges at room temperature (i.e., 20 to 25 ºC) on the incubation tray for at least 18 minutes and acquire samples within 2 hrs. from blood addition to the cartridges.

5.3.3. Enrolled Sample Acquisition and Analysis

- Enter the specimen ID using the touch screen as instructed in the workflow note instructions.
- Once incubation is finished, load the IUO BD cartridge into the instrument. The instrument will automatically read and calculate AbsCD4, %CD4, and Hb concentration.
- Print specimen results. Review the acquisition data for acceptability and check for quality controls messages, if there is quality control message, refer to IFU for troubleshooting.

5.3.4. Acquisition Schedule for Precision Procedure

Acquire QC Control and Sample cartridges per the following run order, using the separate set of quality control and precision samples stained for each precision run. The two runs must be separated by a minimum of 2 hours. This is defined as the time between the acquisition time for the last cartridge in the first run and the acquisition time for the first cartridge in the second run.

Table 1: Run Order

Precision Run1	Streck CD Chex Plus	Multi-Check normal (n=1) control<br>Multi-Check CD4 low (n=1) control<br>Streck CD- Chex Plus normal (n=1) sample<br>Streck CD-Chex Plus CD4 low (n=1) sample<br>Streck CD- Chex Plus CD4 low (n=1) sample<br>Streck CD- Chex Plus normal (n=1) sample
Precision Run 1	Eurotrol	Eurotrol Control level 1 (n=1) control<br>Eurotrol Control level 2 (n=1) control<br>Eurotrol Control level 3 (n=1) control<br>Eurotrol Control level 1 (n=1) sample<br>Eurotrol Control level 3 (n=1) sample<br>Eurotrol Control level 2 (n=1) sample<br>Eurotrol Control level 2 (n=1) sample<br>Eurotrol Control level 3 (n=1) sample<br>Eurotrol Control level 1 (n=1) sample
Precision Run 2	Streck CD Chex Plus	Multi-Check normal (n=1) control<br>Multi-Check CD4 low CD4 low (n=1) control<br>Streck CD- Chex Plus CD4 low (n=1) sample<br>Streck CD- Chex Plus normal (n=1) sample<br>Streck CD- Chex Plus normal (n=1) sample<br>Streck CD- Chex Plus CD4 low (n=1)sample
Precision Run 2	Eurotrol	Eurotrol Control level 1 (n=1) control<br>Eurotrol Control level 2 (n=1) control<br>Eurotrol Control level 3 (n=1) control<br>Eurotrol Control level 2 (n=1) sample<br>Eurotrol Control level 1 (n=1) sample<br>Eurotrol Control level 1 (n=1) sample<br>Eurotrol Control level 3 (n=1) sample<br>Eurotrol Control level 3 (n=1) sample<br>Eurotrol Control level 2 (n=1) sample

6. DISCONTINUATION OF SPECIMEN TESTING

Not applicable – only control material is used for this study. Control materials must be used within expiration dating.

7. PRODUCT ERRORS/ DEFECTS/ FAILURES

Errors, defects or unanticipated failures observed for the investigation study product(s) will be documented using the study error forms and communicated immediately to the Study Monitor.

These events will be documented and escalated in accordance with BD internal procedures.

8. DATA COLLECTION AND MANAGEMENT

8.1. Data Collection and Source Documents

Study data will be collected in the form of standardized case report forms (CRFs) and other data collection forms that will be provided to the investigational site. Instrument data will also be collected in the form of instrument records (electronic files or printouts) such as from instrument set-up and sample acquisition results; these are considered source documents.

Investigators will maintain all source documents associated with the protocol, including laboratory results and instrument reports. All hard copy and electronic data will be secured to ensure confidentiality. The source documents will be used for study monitoring and data management review to verify study compliance.

In verifying data integrity, the monitor will ensure that the data in the CRFs are consistent with protocol requirements.

8.2. Submission of Data to Sponsor

8.2.1. Data Packages

Investigator is responsible for the collection, review, and timely submission of all required study data to BD for processing, including instrument electronic files, which will be acquired and saved in specified study archives.

All study data that include paper and electronic documentation of the study, will be submitted to BD in a weekly package by courier. The PI or designee will ensure that the data and documentation are accurate, complete, and in compliance with applicable documentation requirements, prior to assembling and submitting data packages to BD.

BD will provide a checklist during site initiation and training that can be referenced during data package compilation and review. This checklist may contain, but not be limited to:

- List of the minimum required package items

Quality assurance procedures should be followed to ensure that complete, accurate and timely data are submitted, that protocol requirements are followed, and that product failures, errors, adverse events and adverse device effects are detected, managed and reported as required, if applicable.

Upon completion of data package review, the site will forward this package to BD in regular shipments at intervals agreed upon by the CRA and PI (typically, once per week).

8.3. Data Evaluation for Inclusion/Exclusion from Analysis

After receipt of data packages from the sites, the BD CRA/study monitor or designee will review the contents to ensure that the data are accurate, complete, and in compliance with all applicable requirements.

A complete dataset is required for each sample in the study. A dataset that does not meet all requirements for evaluability as defined in this section may be excluded from analysis. All excluded samples will be justified in the study final report.

- Instrument setup results must “Pass” for each day’s data collection. Precision sample results obtained when the instrument setup result does not pass will be excluded from the study.
- Control results must be within range for each day’s data collection. Precision sample results obtained when the quality control samples fail will be excluded from the study.
- Duplicate measurements must be obtained for the precision samples. Results with only one replicate will be removed.
- Results obtained with methods not in compliance with all applicable protocol, Instructions For Use, and User’s Guide requirements will be excluded.
- Statistical outliers will be investigated and reported. Outlier results with assignable causes may be excluded pending investigation.

In instances where data is found to be missing or incomplete, the CRA will contact the site to resolve data discrepancies using a data clarification form (DCF) for query resolution. Discrepancies may be addressed via calls to the investigational site or during visits by site monitors. Based on the outcome of this review, the CRA may identify and provide a reason for specified data to be deemed not evaluable and possibly excluded from analysis (but not removed from the study database).

8.4. Data Management and Storage

BD Data Management will be responsible for the entry, processing and maintaining of study data.

All original CRFs will be sent to BD as part of the periodic submission package or be retrieved by the site monitor with a copy kept at the site. BD Data Management will process the data and will carry out edit checks and error checks, if needed. The Investigator will be queried on issues concerning data completeness and consistency.

All above-mentioned tasks will be performed according to the Sponsor’s relevant internal procedures; ensuring adherence to GCPs.

9. STATISTICAL METHODS

Statistical analyses will be conducted by the BD Biostatistics group.

9.1. Sample Size Determination

The sample size for both Repeatability and Precision Procedures are based on CLSI guidance document EP5-A2.

For Repeatability procedure twenty replicates of test materials (two concentration levels) are required to run on one instrument by one operator in one day.

For the precision procedure forty-two (42) runs (2 runs per day times 21 days) are required. Each run will consist of duplicate values for the each precision sample (Streck CD Chex normal and low, and level 1, 2, 3 of Eurotrol) and a single value for each process control. Additional runs may be conducted in excess of those noted in anticipation of non-evaluable data. The EP5-A2 guidance document recommends testing two concentrations of test material and one quality control sample per run. Two quality control (BD Multi-check normal and CD4 low and Eurotrol level 1, 2, 3) and two precision samples (Streck CD-Chex plus low and normal) and Eurotrol (level 1, 2 3) will be used in this evaluation. A separate set of quality control and precision samples will be stained for each precision run.

9.2. Eligibility, Exclusions, Missing Data, Interim Analysis

All specimens meeting the eligibility criteria and tested with the IUO BD FACSPresto System will be analyzed for primary and secondary endpoints. Management of dropouts and missing data will depend on their frequency and the particular outcome measure; any such adjustments will be described

completely and documented. All valid data will be included in the analysis; if outliers are determined to exist, the monitor will investigate the identified outliers.

No interim analyses are planned since study duration is 21 working days.

9.3. General Statistical Considerations

All statistical analyses will be performed using commercially available statistical software. Adequate source document verification and/or audit activities will be utilized to assure the validity of study conclusions. Analysis shall be performed based on methods described in Statistical Analysis Plan, briefly described in section 9.7 Analysis Method of this protocol. All related and resulting reports, documents and data shall be produced and maintained in such a way as to ensure their control and the protection of subject privacy as far as is reasonably practicable. Data files and analytic reports will be archived according to requisite regulatory standards.

9.4. Study Endpoints / Outcome Measures

9.4.1. Primary endpoint

Repeatability Procedure

The primary endpoint of the Repeatability procedure is evaluation of closeness of agreement of the FACSPresto™ system measuring AbsCD4+ and %CD4+ cells using two levels of Streck CD checks plus controls under repeatability conditions.

9.4.2. Precision Procedure

The primary endpoint of the Precision procedure is to determine precision performance of the BD FACSPresto™ system measuring percentage and absolute counts of CD4+ cells using two levels of Streck CD checks plus control during a minimum 21 days of testing.

9.4.3. Secondary Endpoints

Repeatability Procedure

The secondary endpoint of the repeatability procedure is evaluation of closeness of agreement of the BD FACSPresto™ system in measuring Hb concentration using 2 or more levels of Eurotrol under repeatability conditions.

Precision Procedure

The secondary endpoint of the precision procedure is determination of precision of the BD FACSPresto™ system (instrument, software, and BD CD4/%CD4/Hb Cartridge Assay) in measuring measuring Hb concentration using three levels of Eurotrol during a minimum twenty one days of testing.

9.5. Acceptance Criteria

The evaluation cited in EP5-A2 Guideline will be used to estimate Repeatability and total precision. Total standard deviation (SD) and coefficient of variation (CV) of the following measurements will be computed for:

- Absolute count of CD4+ cells
- CD4+ as a percentage of lymphocytes
- Hemoglobin Concentration

The acceptance criteria for absolute counts and percentage at 95% confidence bound of the SD or the CV should be:

Table 2: Acceptance Criteria

	>50 to < 200* cells/µL	≥ 200☼ cells/µL
Absolute count of CD4+ cells	CV ≤ 20%	CV ≤ 10%
	< 25%^	≥ 25%¥
%CD4+ of total lymphocyte	SD ≤ 2.5% absolute	CV ≤ 10%

* Design Input Document v3.0, MICD4-DID-383
☼ Design Input Document v3.0, MICD4-DID-384
^ Design Input Document v3.0, MICD4-DID-385
¥ Design Input Document v3.0, MICD4-DID-386

The Hemoglobin concentration results shall be precise within a coefficient of variation (CV) of less than or equal to 7% with 95% confidence (Design Input Document v3.0, MICD4-DID-397).

9.6. Methods to Reduce Bias

To minimize operator error or bias, all operators participating in the study will have documented training in the procedures for cell staining, instrument set up, sample acquisition and sample analysis.

9.7. Analysis Method

Total precision is a combination of several sources of variability, including within-run, between-run, between-instrument, between-operator and between-day. The within-run and total precision will be calculated for the absolute count of CD4+ cells, the percent CD4+ of CD45+ cells, and Hb concentration. The upper 95% confidence interval on the within-run and total precision will be calculated.

For Repeatability procedure the within-run repeatability will be calculated.

9.7.1. Statistical Quality Control Chart

Statistical quality control charts should be set up per the following procedures as described on CLSI EP5-A2. AbsCD4, %CD4 from both levels of Multi-Check controls, and Hb consentration from 3 levels of Eurotrol controls will be used for quality control.

- Calculate center lines, warning limits, and out-of-control limits from initial data collection (5 days)
- Plot all subsequent quality control data on the charts
- If at any point an out-of-control condition is detected, determine the cause, eliminate the data point, and then repeat the run. It is suggested that since there is a low statistical power with these preliminary estimates, that ±3 SDs (standard deviations) be used as indications for investigation and ±4 SDs be used for rejection. It is not acceptable to simply rerun a control sample to see if the new point is inside the control limit.
- After each of 5 days data collection, recalculate the center lines and control limits of each chart from all acceptable data collected thus far.
- If the previous acceptable results are now unacceptable, continue the precision experiment to obtain the proper number of days.
- Maintain a record of the number of rejected runs.

9.7.2. Outlier Check

Outliers will be checked based on method described in CLSI EP5-A2 section 10.7 using data from this study for the outlier test. If more than 5% of the runs need to be rejected and no assignable cause can be found, this may indicate that the device is not sufficiently stable to allow a valid variability assessment.

9.8. Biostatistics Report

A statistical report will be prepared by the biostatistician after data analysis is complete.

10. STUDY MATERIALS AND SUPPLIES

10.1. Investigational Product Labeling

The investigational products will be provided bearing “IUO” label/labeling indicating that they must only be used for investigational purposes (i.e. “For Investigational Use Only” labeling). The BD FACSPresto System (instrument, cartridges, and software) will be provided bearing labels/labeling indicating “IUO”. Additional information regarding system operation and reagent composition will be contained in IUO instructions for use and package inserts that will be provided to the site prior to or during study initiation.

Additional information regarding product composition, handling, etc. will be contained in an IUO package insert.

10.2. Study Products Shipment and Receipt

BD will include appropriate shipping log(s) in all material shipments to the sites: Study Accountability Log(s) for IUO/IVD Instrument, Reagents, Software; and/or Miscellaneous Materials Shipment Log(s).

All IUO or IVD supplies for training and for the study will be verified upon delivery by site personnel and entered on the center's Study Accountability Logs. After receipt has been documented on Study Accountability logs, the log originals are retained by the site to allow for documentation of usage and final disposition of all investigational materials (i.e., used, unused and destroyed at the site, or unused and returned to BD).

10.3. Study Product Accountability

The Sponsor will provide the Investigator with investigational product upon receipt of all necessary study documentation.

During the investigation, the Investigator is responsible for ensuring that:

- only specimens that qualify for study enrollment are run on the IUO system;
- at the testing laboratory, all investigational products and devices are maintained under controlled access storage, and the use of investigational products are strictly controlled;
- any device or components that are removed from their packaging and not used are segregated from general use for the duration of the study.

The Investigator is responsible for maintaining accurate accountability records for the use of investigational materials on the Study Accountability Logs. These records will be reviewed at all monitoring visits to ensure compliance, and will document:

- Receipt dates and quantity,
- Dates and quantity used, and
- Amount used, discarded or returned.

Study Accountability Logs will be verified by the site monitor against the Shipping Logs.

10.4. Required Study Materials and Supplies

10.4.1. Instrumentation

BD will provide IUO products:

- IUO BD FACSPresto instrument and accessories (incubation tray)

10.4.2. Reagents, and Controls

- BD Multi-Check normal and CD4 low controls

- CD-Chex Plus/ Chex CD4 Low
- Eurotrol 301 (level 1, 2, and 3))
- BD CD4/%CD4/Hb cartridges or IUO BD cartridges

10.4.3. General Product Use and Study-Specific Documents

- IUO BD FACSPresto System IFU
- IUO FACSPresto Safety and Limitations Guide
- IUO BD cartridge IFU

10.4.4. Ancillary Items

BD will provide the following:

- Transfer pipettes
- CDs or USB memory sticks for archival of the BD FACSPresto result files
- BD FACSPresto printer rolls

Site should have access to the following:

- Vortex mixer
- Lab timer

10.5. Disposition

At the end of the study or upon request by the site monitor, any remaining investigational devices, used or unused, will be handled as follows:

10.5.1. Reagents

The study monitor will instruct whether all used IUO BD cartridges, as well as those opened but not used, will be returned or defaced and discarded at the site. No experimental devices or supplies will be used for any other purpose except for testing the specimens enrolled in this clinical study.

Unused reagents: At the end of the study or as instructed by the site monitor, the Investigator or his/her designate will return or deface and discard all remaining unused supplies to BD.

11. MONITORING

11.1.1. Study Monitors

The study monitor's responsibilities are to ensure that:

- the study protocol is followed,
- timely and accurate data are submitted,
- inconsistent, incomplete or inaccurate data is corrected,
- site facilities, staff and performance continue to be sufficient to ensure the validity of the scientific data, adherence to study requirements, and the protection of the health, safety and welfare of the study subjects.
- prompt reporting as required by the investigational plan and FDA regulations is achieved, especially regarding Unexpected Adverse Device Effects (21CFR part 812.50)

Study monitoring will comply with the requirements of GCP Guidelines and Section 9 of ISO 14155:2011, Clinical investigations of medical devices. Study monitoring will be performed by qualified personnel, with appropriate education and / or experience to perform all specified and necessary monitoring tasks.

11.2. Monitoring Procedures

11.2.1. Case Report Forms (CRFs) Management

The site monitor will ensure that CRFs are completed for each included specimen in a timely fashion. Electronic files and instrument printouts will be monitored on-site and in-house.

Corrections and modifications made to data already written or completed on the CRF page should be legible, initialed and dated by approved personnel. The reasons for significant changes must be provided. Correction fluid or covering label must not be used.

11.2.2. Monitoring Procedure and Documentation

Monitoring visits will be conducted in accordance with Clinical Operations Standard Operating Procedures (SOPs), Desk Procedures (DPs), the study protocol, monitoring plan and applicable regulatory requirements. Monitoring visits to the study site will be scheduled periodically during the study, to ensure that Investigators and their staff understand and accept their defined responsibilities and that all aspects of the current approved protocol/amendment(s) are followed. Data collection forms, electronic files and study data will be reviewed for cross referencing for accuracy.

The Investigator/institution guarantees access to study documents and source documents related to the study by designated BD personnel and appropriate regulatory authorities.
It is important that the Investigator and their relevant personnel are available during the monitoring visits and possible audits and that sufficient time is devoted to the process. The Site monitor will complete a report documenting completion of the each site visit.

All site visits will be recorded in a monitoring log maintained at each investigational site documenting the monitor and the date of the monitoring visit.

- Site Initiation Visit: Prior to a clinical investigation, the BD monitor will ensure that the Investigator and the investigational site are prepared for the study. All the appropriate training materials and the user documentation for this clinical investigation will be provided to study personnel during the site initiation visit. Documentation of all necessary approvals, agreements, and training on the study and experimental treatment will be gathered during the visit.
- Interim Site Visit: A BD monitor will schedule and conduct Interim Monitoring Visits to monitor the clinical investigation throughout its active phase to ensure compliance with this protocol, IUO device accountability and data accuracy. The first Monitoring Visit during the active phase of the study will be scheduled according to the study monitoring plan.
- Site Close-out Visit: The BD monitor will assure that all site closeout activities are addressed when the study has been completed or terminated at the clinical site as indicated in the study monitoring plan.
- Compliance visits: In the event that a study site requires additional assistance to meet study protocol or regulatory requirements, monitoring visits will be conducted at more frequent intervals and / or with additional personnel to assure compliance.
- Other visits: the study site may be inspected by appropriate regulatory authorities this includes visits by Ministry of Health representative(s).

11.3. Audits/Inspections

The site may be selected for an audit by the sponsor (e.g., BD Medical and Quality Assurance groups) or by a regulatory authority. It is responsibility of the PI to facilitate the auditing process by granting all required access to the study site, study documentation, source data and study personnel. When the PI is notified of an upcoming audit by a regulatory authority, he/she must notify BD as soon as possible.

12. SAFETY REPORTING

Injuries or safety issues related to the use of an investigational study product(s) must be reported immediately to the Sponsor. This report should provide details of any unusual event concerning an operator/user of the device/system and the steps taken to provide treatment or abatement of any injuries or correction of the event.

Upon receipt, the Sponsor will evaluate each report to determine if the event could affect the integrity of the study data or the operation of the system, or if it meets criteria of an "Adverse Event" as determined by BD Medical and Regulatory Affairs. All Serious Adverse Events (SAE) must be reported by the Principal Investigator to the EC/IRB (and, if appropriate, any regulatory agency) within the specified time periods described in local/country regulations.

All adverse events occurred during the conduct of the study will be reported to Sponsor using the appropriate form.

13. STUDY SUSPENSION OR TERMINATION

Either the BDB Clinical Operations or BDB program team may terminate this study for a reasonable cause. Reasons for suspension or early termination may include the following:

- Previously unanticipated and insurmountable administrative or scientific difficulties or obstacles
- Change in BDB project priorities

14. STUDY ETHICS/ GOOD PRACTICES

The procedures set forth in this study protocol are designed to ensure that BD and clinical investigators abide by the ICH GCP Guidelines, regulations of the FDA, and the Declaration of Helsinki in the conduct, evaluation and documentation of the study.

14.1. Ethics Review and Approval

IRB approval is not required for this study, as only commercial control materials are used for testing.

14.2. Informed Consent

Informed consent is not applicable for this study, as only commercial control materials are used for testing.

14.3. Risks and Benefits

There are no specimen donors required for this study, as all testing will be performed using commercial control material. All specimens should be handled as if capable of transmitting infectious agents. Operators must follow any additional site-specific biohazard safety requirements.

14.4. Donor Identification and Confidentiality

This is not applicable since commercial control will be used fro this study.

14.5. Donor Remuneration

This is not applicable for this study.

15. INVESTIGATOR

15.1. Clinical Investigators

The Clinical Investigator and staff will meet GCP requirements. The Investigator, approved sub-Investigators, study operators and all supporting staff at the Investigator's site must be appropriately qualified by education and / or experience to perform their tasks.

15.2. Submission and obtaining Ethics Approval

This is not applicable for this study.

15.2.1. Source Documents

Case Report Forms and instrument data will be source document for this study.The Investigator agrees that the Study monitors, Sponsor's employees, contractors or designees, as well as any regulatory bodies as required, will have the right to audit and review source documents and study records relating to this clinical trial. The Investigator and staff will assist with the production, review, interpretation and / or correction of such records as required.

15.2.2. Device Accountability

Investigator or designee will maintain an accurate Accountability Log(s) and records of the investigational product received, used and unused. Study supplies must be used only for specimen enrollment and testing described in this protocol.

Principal investigator must supervise the use of investigational device only for the purpose of this study and under the Investigator's supervision. An Investigator shall not supply an investigational device to any person not authorized to receive or use it.

15.2.3. Data Recording and Record Retention

The Investigator(s) will ensure that the Case Report Forms, instrument records/ printouts, and other study documents are maintained in a secure and confidential manner. The Investigator(s) will ensure that the medical records, Case Report Forms and other study documents are made available for review by the study monitor and any government regulatory bodies as required.

The Sponsor or designee will ensure accuracy and completion of CRFs and hard copies are maintained in the study files.

All essential study documentation (e.g., CRFs, other data collection forms, instrument printouts, and electronic files) must be retained by the PI for a minimum of two years after notification from BD that the study is completed, terminated, or that the investigational product has been successfully released for commercialization, whichever is the longer time period. In the instance where the study PI withdraws as the responsible party, written notification containing the contact information of the PI designee must be forwarded to BD.

As the study sponsor, BD will retain all essential study documentation indefinitely (either in-house or off-site).

15.3. Investigator Reports

Controls will be used for this study so this section is not applicable.

15.3.1. Withdrawal of EC/IRB Approval

Controls will be used for this study so this section is not applicable.

15.3.2. Deviation from the Study Protocol

The Investigator shall notify Sponsor of any changes in, or deviations. BDB will determine the effect of the protocol deviation on the scientific soundness of the clinical data and document according to internal procedures.

15.3.3. Use of Device without Informed Consent (applicable if Informed Consent is required)

Controls will be used for this study so this section is not applicable.

15.3.4. Final Report

Not applicable for this study.

15.3.5. Other Reports

Not applicable for this study.

15.4. Responsibility for Investigator Reports

The Principal Investigator may delegate a qualified associate(s) to complete one or more of the above functions. However, the Principal Investigator retains the overall responsibility for the proper conduct of the study, compliance with this study plan, and the collection of all required data, as well as subject safety where applicable.

15.5. Financial Disclosure

A clinical Investigator shall disclose to the Sponsor sufficient accurate financial information to allow the applicant to submit complete and accurate certification or disclosure statements required by the regulations. The Investigator shall promptly update this information if any relevant changes occur during the course of the investigation and for 1 year following completion of the study.

16. SPONSOR

16.1. Sponsor Staff Qualifications

The BD Clinical Operations employees and contractors, including study monitors, will be appropriately qualified by education and / or experience to perform their tasks. Resources, systems, standard operating procedures and training adequate to oversee the scientific, ethical and regulatory aspects of the clinical study will be maintained throughout the period of the clinical investigation.

16.2. Beginning the Study

Before the start of study and testing, BD must have the following documents from the clinical site:

- CVs signed and dated from the Principal investigator and site staff participating in the trial (Sub-Investigator, Technologists, etc.)
- Approved Investigator's Agreement
- Principal investigator and Sub-Investigator financial disclosure

16.3. Selecting Investigators

Sponsor shall select Investigators qualified by training and experience to investigate the Precision Evaluation of the IUO BD FACSPresto System and IUO BD FACSPresto cartridge.

16.4. Ensuring Proper Monitoring

Sponsor shall select monitor(s) qualified by training and experience to monitor the progress of the investigation.

16.5. Ensuring Proper Notification

Not applicable for this study.

16.6. Control of Device

Sponsor shall ship investigational devices only to qualified Investigators participating in the study.

16.7. Obtaining Agreements

Sponsor shall obtain a signed and dated Investigator Agreement; the Investigator's CV; a statement of the Investigator's relevant experience (if applicable), including dates, location and type of experience; explanation of circumstances leading to termination of studies previously undertaken by the Investigator (if applicable); a statement that the Investigator is committed to conduct the investigation in accordance

with the agreement, the investigational plan, other applicable FDA regulations, and other conditions imposed by the Ethics Committee/FDA.

16.8. Securing Compliance

Sponsor must ensure that the Investigator continues to comply with the signed agreement, the investigational plan, other applicable FDA regulations and other conditions imposed by the Ethics Committee/FDA, or discontinue shipments of device to the Investigator, terminate the Investigator's participation in the investigation and require such an Investigator to return device, unless this action would jeopardize the rights, safety or welfare of a subject.

16.9. Resumption of Suspended or Terminated Studies

Either the BDB Clinical Operations or BDB program team may terminate this study for a reasonable cause. Reasons for suspension or early termination may include the following:

- Previously unanticipated and insurmountable administrative or scientific difficulties or obstacles
- Change in BDB project priorities

16.10. Reporting

A Final Study Report meeting the requirements of ISO 14155:2011, Annex C and relevant country regulations will be prepared by the Sponsor.

16.11. Records

Sponsor or delegate shall maintain accurate, complete, and current study documentation.

17. PUBLICATION

Publication of the study results in the medical literature will be attempted, under the authorship of the Investigators; however; BD reserves the right to comment on publications prior to submission in order to protect intellectual property rights and confidential information.

18. REFRENCES

1. E6, International Conference on Harmonization: Good Clinical Practice: Consolidated (Published in the Federal Register May 9, 1997)
2. Estaquier J, Idziorek T, de Bels F, Barré-Sinoussi F, Hurtrel B, Aubertin AM, Venet A, Mehtali M, Muchmore E, Michel P, Mouton Y, Girard M, Ameisen JC. Programmed cell death and AIDS: significance of T-cell apoptosis in pathogenic and nonpathogenic primate lentiviral infections. Proc Natl Acad Sci U S A. 1994 Sep 27;91(20):9431-5.
3. Antiretroviral Therapy of HIV Infection in Adults and Adolescents: Recommendations for a Public Health Approach. World Health Organization, 2010 revision.
4. Volberding PA, Lagakos SW, Koch MA, Pettinelli C, Myers MW, Booth DK, Balfour HH Jr, Reichman RC, Bartlett JA, Hirsch MS, Zidovudine in asymptomatic human immunodeficiency virus infection. A controlled trial in persons with fewer than 500 CD4-positive cells per cubic millimeter. The AIDS Clinical Trials Group of the National Institute of Allergy and Infectious Diseases. N Engl J Med. 1990 Apr 5;322 (14):941-9.
5. Cohen MS, Chen YQ, McCauley M, Gamble T, Hosseinipour MC, Kumarasamy N, et al. Prevention of HIV-1 infection with early antiretroviral therapy. N Engl J Med 2011; 365(6):493-505.
6. Thomas TK, Masaba R, Borkowf CB, Ndivo R, Zeh C, Misore A, et al. Triple-antiretroviral prophylaxis to prevent mother-to-child HIV transmission through breastfeeding--the Kisumu

Breastfeeding Study, Kenya: a clinical trial. PLoS Med 2011; 8(3):e1001015.

7. Henry DH, Beall GN, Benson CA, Carey J, Cone LA, Eron LJ, Fiala M, Fischl MA, Gabin SJ, Gottlieb MS, et al. Recombinant human erythropoietin in the treatment of anemia associated with human immunodeficiency virus (HIV) infection and zidovudine therapy. Overview of four clinical trials. Ann Intern Med. 1992 Nov 1;117(9):739-48.

8. Firnhaber C, Smeaton L, Saukila N, Flanigan T, Gangakhedkar R, Kumwenda J, La Rosa A, Kumarasamy N, De Gruttola V, Hakim JG, Campbell TB. Comparisons of anemia, thrombocytopenia, and neutropenia at initiation of HIV antiretroviral therapy in Africa, Asia, and the Americas. Int J Infect Dis. 2010 Dec; 14(12):e1088-92. Epub 2010 Oct 18

9. Clinical and Laboratory Standards Institute. Evaluation of Precision Performance of Quantitative Measurement Methods; Approved Guideline, Second edition. CLSI document EP5-A2. Vol 24, No. 25, 2004.

19. CHANGE HISTORY

Version	Date	Name	Change Description
1.0	29JUL2013	Maryam Saleminik	Initial Version
