# Supplementary material for: Clinical Evaluation of the BD FACSPresto™ Near-Patient CD4 Counter in Kenya
Source: PLoS One. 2016 Aug 2;11(8):e0157939. doi: 10.1371/journal.pone.0157939 (PMC4970792; doi:10.1371/journal.pone.0157939)
Supplement: S3 File — (PDF) [file pone.0157939.s004.pdf]

Clinical Study Protocol

Linearity Evaluation of the Investigational BD FACSPresto™ System: Instrument, Software, and BD CD4/%CD4/Hb Cartridge

Protocol Number: CAS-PCLIN
Version: 1.1
Date: 17/DEC/2013

Principal Investigators
Laurie Byrne
Medical Laboratory Manager
Phone: (408) 954-6069
Fax : (408) 954-6313
Email:Laurie_Byrne@bd.com

Study Sites
BD Biosciences, MEDLab
2350 Qume Drive
San Jose, CA 95131

Sponsor
BD Biosciences
2350 Qume Drive
San Jose, CA 95131

For Investigational Use Only

The Performance characteristics of this product have not been established.

All investigational material supplied by BD Biosciences must be used for study purposes only. Results from investigational testing must not be used as a diagnostic procedure without confirmation of the diagnosis by another medically established diagnostic product or procedure.

Confidential

The product information and data disclosed through this study protocol are confidential and are subject to a Confidential Disclosure Agreement. No content may be disclosed without prior written consent of Becton, Dickinson and Company.

SPONSOR

BD Biosciences
2350 Qume Drive
San Jose, CA 95131

Medical Affairs	*Kevin Judge, MD*<br>*VP of Medical Affairs and Medical Monitor*<br>Phone: 201-847-4454<br>Email: kevin_judge@bd.com
Clinical Operations	*Lorlelei Lee-Haynes, MPH*<br>*Director of Clinical Operations*<br>Phone: 408-954-6309<br>Fax: 408-954-6313<br>Email: lorlelei-lee_haynes@bd.com
CPL / Study Manager	*Imelda Omana-Zapata, MD, PhD*<br>*Clinical Project Lead*<br>Phone: 408-954-2323<br>Fax: 408-954-6313<br>Email: imelda_omana-zapata@bd.com
CRA / Study Monitor	*Maryam Saleminik*<br>*Clinical Research Associate*<br>Phone: 408-518-5037<br>Fax: 408-954-6313<br>Email: MSaleminik@bd.com
Biostatistics	*Beverly Lu*<br>*Biostatistician*<br>Phone: 408-954-6301<br>Email: beverly_lu@bd.com
Data Management	*Jeannine Paliotta*<br>*Data Manager*<br>Phone: 408-954-4111<br>Email: Jeannine_Paliotta@bd.com

CHANGE OVERVIEW

Section	Rationale	Version 1.0	Version 1.1
9.5.1	DID updated to Version 6.0 and this protocol updated to reflect changes for Acceptance criteria based on DID changes	Across the dynamic range of the assay for AbsCD4 (50 to 4,000 cells per µL), the system shall be linear if 2nd and 3rd order coefficients of the regression lines are not significant. If the coefficients from the higher order polynomial fit (2nd and 3rd) tested are statistically significant, then the difference between the first order linear fit and the higher order linear fit must be within 10% of the first order linear fit for concentration levels that are >100 cells/ µL, or within ±15 cells/µL of the first order linear fit for concentration levels that are ≤100 cells/ µL. (Design Input Document v4.0, MICD4-DID-388	Across the dynamic range of the assay for AbsCD4 (50 to 4,000 cells per µL), the system shall be linear if 2nd and 3rd order coefficients of the regression lines are not significant. If the coefficients from the higher order polynomial fit (2nd and 3rd) tested are statistically significant, then the difference between the first order linear fit and the higher order linear fit must be within 10% of the first order linear fit for concentration levels that are >200 cells/ µL, or within ±20 cells/µL of the first order linear fit for concentration levels that are ≤200 cells/ µL. The coefficient of determination ((R ^ 2) shall be >95% for linear fit. (Design Input Document v6.0, MICD4-DID-388)
9.5.2		Across the dynamic range of the assay for total lymphocte count (200 to 10,000 cells per µL), the system shall be linear if 2nd and 3rd order coefficients of the regression lines are not significant. If the coefficients from the higher order polynomial fit (2nd and 3rd) tested are statistically significant, then the difference between the first order linear fit and the higher order linear fit must be within 10% of the first order linear fit for concentration levels that are >200 cells/ µL, or within ±20 cells/ µL for concentration levels that are ≤200 cells/ µL. (Design Input Document v.4.0, MICD4-DID-467)	Across the dynamic range of the assay for total lymphocte count (200 to 10,000 cells per µL), the system shall be linear if 2nd and 3rd order coefficients of the regression lines are not significant. If the coefficients from the higher order polynomial fit (2nd and 3rd) tested are statistically significant, then the difference between the first order linear fit and the higher order linear fit must be within 10% of the first order linear fit for concentration levels that are >200 cells/ µL, or within ±20 cells/ µL for concentration levels that are ≤200 cells/ µL. The coefficient of determination ((R ^ 2) shall be >95% for linear fit. (Design Input Document v.6.0, MICD4-DID-467)
9.5.3		Across the dynamic range of the assay for Hb concentration (2 to 20 g/dL), the system shall be linear if 2nd and 3rd order coefficients of the regression lines are not significant. If the coefficients term from higher order polynomial fit (2nd and 3rd order terms) tested are statistically significant, then the difference between the first order linear fit and the higher order linear fit must be < 0.5 g/dL from the first order linear fit for each concentration level. (Design Input Document v4.0, MICD4-DID-393)	Across the dynamic range of the assay for Hb concentration (2 to 20 g/dL), the system shall be linear if 2nd and 3rd order coefficients of the regression lines are not significant. If the coefficients term from higher order polynomial fit (2nd and 3rd order terms) tested are statistically significant, then the difference between the first linear fit and the higher order linear fit must be < 7% different from the linear fit, except for Hb ≤ 7.1 g/dL, at which point it must be within 0.5 g/dL from the first order linear fit at each level for Hemoglobin counts. The coefficient of determination ((R ^ 2) shall be >95% for linear fit. Design Input Document v6.0, MICD4-DID-393)

TABLE OF CONTENTS

1. INTRODUCTION 8

1.1. Statement of Compliance 8

1.2. Background Information and Scientific Rationale 8

1.3. Proposed Intended Use 8

2. OBJECTIVES 8

2.1. Primary Objective(s) 8

2.2. Secondary Objective(s) 9

3. STUDY DESIGN 9

3.1. Overall Study Design / Outcome Measures 9

3.1.1. CD4+ T-lymphocyte absolute count 9
3.1.2. Total lymphocyte count 9
3.1.3. Hemoglobin concentration 10

3.2. Expected Study Duration 10

3.3. Study Device(s) 10

3.3.1. Investigational Device(s) 10
3.3.2. Predicate Device 10

4. STUDY POPULATION 10

4.1. Specimen Requirements 10

4.2. Inclusion and Exclusion Criteria 10

4.3. Sample Size 11

5. STUDY METHODS/PROCEDURES 11

5.1. Daily Study Activities 11

5.1.1. Instrument Setup/ Instrument QC 11
5.1.2. Process Controls 11

5.2. Specimen Enrollment 11

5.3. Enrolled Specimen Preparation 11

5.3.1. CD4+ T lymphocyte Preparation 11
5.3.2. Total Lymphocyte Preparation 12
5.3.3. Hemoglobin Concentration Preparation: 13
5.3.4. Sample Testing 13
5.3.5. Enrolled Sample Acquisition and Analysis 13

6. DISCONTINUATION OF SPECIMEN TESTING 14

7. PRODUCT ERRORS/ DEFECTS/ FAILURES 14

8. DATA COLLECTION AND MANAGEMENT 15

8.1. Data Collection and Source Documents 15

8.2. Submission of Data Package to Sponsor 15

8.3. Data Evaluation for Inclusion/Exclusion from Analysis 15

8.4. Data Management and Storage 16

9. STATISTICAL METHODS 16

9.1. Sample Size Determination 16

9.2. Eligibility, Exclusions, Missing Data, Interim Analysis 16

9.3. General Statistical Considerations 16

9.4. Study Endpoints / Outcome Measures 16

9.4.1. Primary Endpoint 16
9.4.2. Secondary Enpoints 17

9.5. Acceptance Criteria 17

9.5.1. CD4+ T-Lymphocyte Absolut Count 17
9.5.2. Total lymphocyte count 17
9.5.3. Hemoglobin Concentration 17

9.6. Methods to Reduce Bias 17

9.7. Analysis Method for the Linearity Procedure 17

9.8. Biostatistics Report 18

10. STUDY MATERIALS AND SUPPLIES 18

10.1. Investigational Product Labeling 18

10.2. Study Products Shipment and Receipt 18

10.3. Study Product Accountability 18

10.4. Required Study Materials and Supplies 19

10.4.1. Instrumentation 19
10.4.2. Reagents, and Controls 19
10.4.3. General Product Use and Study-Specific Documents 19
10.4.4. Ancillary Items 19

10.5. Disposition 19

10.6. Reagents 19

11. MONITORING 19

11.1. Study Monitors 20

11.2. Monitoring Procedures 20

11.2.1. Case Report Forms (CRFs) Management 20
11.2.2. Monitoring Procedure and Documentation 20

11.3. Audits/Inspections 21

12. SAFETY REPORTING 21

13. STUDY SUSPENSION OR TERMINATION 21

14. STUDY ETHICS/ GOOD PRACTICES 21

14.1. Ethics Review and Approval 21

14.2. Informed Consent 21
14.3. Risks and Benefits 22
14.4. Donor Identification and Confidentiality 22
14.5. Donor Remuneration 22

15. INVESTIGATOR 22
15.1. Clinical Investigators 22
15.2. Submission and obtaining Ethics Approval 22
15.3. Source Documents 22
15.4. Device Accountability 22
15.5. Data Recording and Record Retention 22
15.6. Investigator Reports 23
15.7. Withdrawal of EC/IRB Approval 23
15.8. Deviation from the Study Protocol 23
15.9. Use of Device without Informed Consent (applicable if Informed Consent is required) 23
15.10. Final Report 23
15.11. Other Reports 23
15.12. Responsibility for Investigator Reports 23
15.13. Financial Disclosure 23

16. SPONSOR 23
16.1. Sponsor Staff Qualifications 23
16.2. Beginning the Study 24
16.3. Selecting Investigators 24
16.4. Ensuring Proper Monitoring 24
16.5. Ensuring Proper Notification 24
16.6. Control of Device 24
16.7. Obtaining Agreements 24
16.8. Securing Compliance 24
16.9. Resumption of Suspended or Terminated Studies 24
16.10. Reporting 24
16.11. Records 25

17. PUBLICATION 25

18. REFRENCES 25

19. CHANGE HISTORY 25

LIST OF ABBREVIATIONS AND DEFINITION OF TERMS

%CD4	Percentage of $CD4^+$ T lymphocytes of total lymphocytes
AbsCD4	Absolute cell count (cells/µL) of all T lymphocytes that are $CD4^+$
AIDS	Acquired Immunodeficiency Syndrome
ART	Antiretroviral therapy
BD	Becton Dickinson and Company. BD Biosciences, hereafter referred to as sponsor
CBC	Complete blood counts
CD4	Antigen present on the “helper/inducer” T lymphocyte subpopulation
CI	Confidence Interval
CRA	Clinical Research Associate
CRF	Case Report Form
CV	Coefficient of Variatioin
DCF	Data Clarification Form
FDA	Food and Drug Administration
GCP	Good Clinical Practice
HIV	Human Immunodeficiency Virus
ICH	International Conference on Harmonization for Registration of Pharmaceuticals for Human Use; as adopted by the FDA (i.e., Good Clinical Practice E6)
ICF	Informed Consent Form
IRB/EC	An Institutional Review Board or Independent Ethics Committee
ISO	International Standard Organization
IUO	Investigational Use Only
IVD medical device	Any medical device which is a reagent, reagent product, calibrator, control material, kit, instrument, apparatus, equipment, or system, whether used alone or in combination, intended by the manufacturer to be used *in vitro* for the examination of specimens, including blood and tissue donations, derived from the human body, solely or principally for the purpose of providing information: concerning a physiological or pathological state, or concerning a congenital abnormality, or to determine the safety and compatibility with potential recipients, or to monitor therapeutic measures.**
Predicate or Predicate Method	The previously approved or generally accepted method
PI	Principal Investigator
QC	Quality Control
UADE	Unanticipated Adverse Device Effect
WB	Peripheral Whole Blood
WBC	White cell blood counts
WHO	World Health Organization

** 98/79/EC:1998, Directive on *in vitro* diagnostic medical devices

1. INTRODUCTION

1.1. Statement of Compliance

The study is to be conducted in compliance with this protocol, Good Clinical Practice (GCP)[1], the Declaration of Helsinki, Title 21 of the Code of Federal Regulations §§ 50, 56, and 812, International Conference on Harmonization E6 and ISO Standards 14155:2011.

1.2. Background Information and Scientific Rationale

The enumeration of T lymphocytes that are positive for the CD4 antigen is used to evaluate the immune status of patients with, or suspected of developing, immune deficiencies such as AIDS. The CD4 antigen is the receptor for Human Immunodeficiency Virus (HIV) and the number of CD4+ T lymphocytes declines due to programmed cell death in an HIV infection[2]. The absolute count of CD4+ T lymphocytes (Abs CD4) and the percentage of CD4+ T lymphocytes of total lymphocytes (%CD4) are cellular parameters closely associated with HIV disease progression and patient prognosis. According to World Health Organization (WHO) guidance documents regarding HIV/AIDS patient treatment, the measurement of Abs CD4 cell counts and %CD4+ in adolescents and adults[3,4] is recommended for decision-making on initiating antiretroviral therapy (ART). Recently, the use of ART has been extended on prevention or prophylaxis.[5,6]

Anemia can be induced by ART[7] or can be one of the hematological abnormalities frequently observed in HIV/AIDS patients in developing countries[8]. Anemia is usually diagnosed by measuring the concentration of hemoglobin (Hb) and by counting the number of red cells or hematocrit in whole blood. It is common practice during the patient’s routine visit to evaluate: the presence of anemia, other hematological or functional parameters to assess HIV/AIDS disease progression or control, and adverse events induced by ART use.

The investigational-use only BD FACSPresto™ System (BD FACSPresto instrument with embedded software and BD CD4/%CD4/Hb cartridge or IUO BD cartridge) is designed to provide enumeration of CD4+ absolute counts (cells/µL) as well as the percentage of the total lymphocyte count that is CD4+ (%CD4) and Hb concentration in g/dL or g/L. Peripheral whole blood and commercialized process controls can be analyzed with this instrument.

The BD CD4/%CD4/Hb cartridge contains dried fluorochrome-conjugated antibody reagent. When whole blood reacts with the reagents, the antibodies in the reagents bind to the surface antigens on the lymphocytes and monocytes. Samples are incubated for eighteen minutes or less than two hours; then after incubation the sample is run on the instrument. The software identifies the cell populations of interest and calculates CD4 absolute counts, CD4 percentages of lymphocytes, and hemoglobin concentration.

The Linearity study design is accordance with the CLSI Guideline CLSI Guideline: EP6-A: Preliminary Evaluation of the Linearity of Quantitative Measurement Procedures: A Statistical Approach; Approved Guideline.[9]

1.3. Proposed Intended Use

The BD FACSPresto™ is an automated system for *in vitro* diagnostic use in performing the direct enumeration of CD4 absolute count, CD4 percentage of lymphocytes, and Hb concentration in human whole blood.

2. OBJECTIVES

2.1. Primary Objective(s)

The primary objective of this evaluation is to demonstrate that the assay is linear across the dynamic range of the assay for CD4+ T-lymphocytes absolute count (50-4,000 cells/µL) and total lymphocyte count (200-10,000 cell/µL) according to the specifications outlined in the Design Input Document (DID).

2.2. Secondary Objective(s)

The secondary objective of this evaluation is to demonstrate that the assay is linear across the dynamic range of the assay for Hb concentration (2-20 g/dL) according to the specifications outlined in the Design Input Document (DID).

3. STUDY DESIGN

3.1. Overall Study Design / Outcome Measures

The study design and proposed analysis for this evaluation follow recommendations given in CLSI Guideline: Evaluation of the Linearity of Quantitative Measurement Procedures: A Statistical Approach; Approved Guideline. CLSI document EP6-A [9]. Typically, Linearity testing requires of eleven samples with evenly spaced concentrations of the analyte of interest. Percent CD4 is not analyte by itself, because is not uniquely determined, the %CD4 values are derived from CD4+ and total lymphocytes measurements. In other words, %CD4+ results cannot be statistically interpolated because it is dependent on both CD4+ and total Lymphocyte populations; therefore, this protocol will evaluate the lymphocyte linearity on the FACSPresto system.

Assay linearity for the BD FACSPresto system will be evaluated for the CD4+ T- lymphocyte absolute count, total lymphocyte absolute count, and Hemoglobin concentration using 3 different lots of BD IUO cartridges as follows:

3.1.1. CD4+ T-lymphocyte absolute count

Assay linearity for CD4+ T-lymphocyte absolute count will be evaluated over 50-4000 CD4+ T lymphocytes/µL using three different lots of IUO CD4/%CD4/ Hb cartridges. To perform this evaluation, two specimen pools (a low and a high concentration) will be prepared containing known concentrations of CD4+ T-lymphocyte. These specimen pools will be proportionally mixed to achieve 11 levels of cell concentrations over a range that is 20-30% wider than the anticipated claim range. Each concentration will then be stained in triplicate and the resulting samples run on a single FACSPresto instrument with replicates run in a randomized order.

3.1.2. Total Lymphocyte Count

In ‘normal’ Complete Blood Count (CBC) the White Blood Cells (WBC) count has a range from 3,500-10,500 cells/µL. The lymphocyte population comprises is 25-35% relative value or 875-3,675 cells/µL absolute value[i]. The Linearity range of 200-10,000 cells/µL is extended well below and above the 875-3,675 cells/µL that is exhibited in a normal individual to artificially imitate the lymphocyte population of a diseased individual.

Linearity for total lymphocyte will be evaluated for the range of 200-10,000 cells/µL on the FACSPresto System using three different lots of IUO CD4/%CD4/ Hb cartridges.To perform this evaluation two specimen pools (low and high cell concentration) containing known concentrations of total lymphocytes. The specimen pools will be proportionally mixed to achieve eleven different cell concentrations over a range that is 20-30% wider than the anticipated claim range. Each concentration will then be stained in

[i] http://www.mayoclinic.com/health/complete-blood-count/MY00476/DSECTION=results; http://www.rnceus.com/cbc/cbcwbc.html

triplicate using CD4/%CD4/Hb and then run on a single FACSPresto instrument with replicate runs in a randomized order.

3.1.3. Hemoglobin concentration

Assay linearity for Hb concentration will be evaluated over 2-20 Hb in g/dL using three different lots of IUO CD4/%CD4/ Hb cartridges. Similarly, two specimen pools (low and high concentration) will be prepared containing known concentrations of hemogolobin. Eleven dilutions of cell concentrations (linearity specimens) over a range that is 20-30% wider than the anticipated claim range will be prepared by mixing high pool (concentrated red blood cells) and low pool (autologous plasma).

Each concentration will then be transferred in triplicate onto BD CD4/%CD4/Hb cartridges and then run on a single FACSPresto instrument with in a randomized order.

Sample preparation and acquisition of each replicate will involve the following:

- Transferring dilutions onto BD CD4/%CD4/Hb cartridge (three cartridges per dilution)
- Incubating cartridges for a minimum of 18 min on the incubation tray.
- Loading cartridges and starting the run within 2 hrs. of blood transfer.

3.2. Expected Study Duration

The anticipated study duration is 1-2 working weeks. Each cartridge lot test will be performed on separate days.

3.3. Study Device(s)

3.3.1. Investigational Device(s)

The investigational devices for this study are the IUO BD FACSPresto instrument with embedded software and IUO BD CD4/%CD4/Hb cartridge that will provide the absolute count of CD4+ T lymphocyte cells/µL of blood (AbsCD4), the percentage of CD4-positive T lymphocytes of total lymphocytes (%CD4) and Hb concentration in g/dL or g/L.

3.3.2. Predicate Device

Not applicable for this study.

4. STUDY POPULATION

4.1. Specimen Requirements

This study will be conducted internally at BDB MEDLab using samples prepared from normal human blood. Normal human blood will be anticoagulated with EDTA or CPT* and obtained from the BD blood collection program.

*Note: Cell Separation Tubes (CPT) will be used only for separation of mononuclear cells (PBMC or buffy coat), plasma, and red blood cells from whole blood. In the CPT tube there is gel barrier that premits cell separation during a single centrifuge. PBMC and plasme will be used as high and low pool and will be mixed together to make diultions.

4.2. Inclusion and Exclusion Criteria

Not applicable – For CD4+ T-lymphocyte and total lymphocte absolute count measurment, linearity specimens will be prepared by mixing a High Pool (concentrated buffy coat) and low pool (autologous plasma). The high and low pool specimens will be created from normal whole blood collected in blood collection tubes with CPT anti-coagulant.

For Hb concentration measurement, linearity specimens will be prepared by mixing a high pool (concentrated red blood cells) and low pool (autologous plasma). The high and low pool

specimens will be from normal whole blood collected in blood collection tubes with EDTA anti-coagulant.

4.3. Sample Size

The sample size for this linearity study is based on CLSI guidance document EP6-A, which requires 7-11 concentrations over a range that is 20 to 30% wider than the anticipated measuring range.

5. STUDY METHODS/PROCEDURES

5.1. Daily Study Activities

Daily study activities include the following:

- FACSPresto QC and Process Controls
- Specimen Enrollment
- Sample Preparation and Acquisition
- Data review and completion of case Report Forms and applicable study logs

5.1.1. Instrument Setup/ Instrument QC

On each day of study testing, turn on the instrument allowing the automatic instrument QC/setup to run, passing results must be obtained prior to running any process controls or samples.

5.1.2. Process Controls

For AbsCD4, CD Chex Plus BC normal and CD4 low will be used as process controls. For Hb concentration, Eurotrol 301 (level 1, 2, and 3) will be used as process control per following steps:

- Label five IUO BD CD4/%CD4/Hb cartridges, one for each level of the process control (CD Chex Plus BC CD4 normal and low, and Eurotrol level 1, 2, and 3) following the naming convention described in the workflow notes instructions.
- Prepare both, CD4 and Hb Process Controls per their package insert and using transfer pipette add 20-25µL well mixed process control to the well of each labeled cartridge.
- Incubate CD-Chex cartridges for a minimum of 18 minutes at room temprature on the incubation tray. Per FACSPresto IFU Eurotrol cartridges do not need to be incubated for 18 minutes and can be run after control blood addition to cartridges.
- Enter the process control information using the touch screen as instructed in the workflow note instructions.
- Once incubation is finished, load one of the IUO BD cartridges into the instrument and start the run.
- Print specimen results and verify that results (i.e., AbsCD4, %CD4, and Hb concentration) fall within the manufacturer's lot-specific ranges.
- Repeat the process with the next IUO BD cartridge.

5.2. Specimen Enrollment

On each day of testing, one whole blood specimen (one donor) will be cerntrifuged as instructed in the workflow note and two specimen pools (a high and low pool) will be prepared. Eleven linearity specimens will be prepared by mixing high and low pool samples as depicted in the workflow note instructions. The applicable CRF will be completed for every specimen dilution.

5.3. Enrolled Specimen Preparation

5.3.1. CD4+ T lymphocyte Preparation:

Assay linearity for CD4+ T-lymphocytes absolute count will be evaluated over 50-4,000 CD4+ T lymphocytes/µL. Two specimen pools will be prepared containing known concentrations of CD4+ T-lymphocytes, low and high concentration pools, and will be proportionally mixed to achieve eleven cell

dilutions over a range that is 20-30% wider than the anticipated claim range; refer to the workflow notes for detailed preparation instructions. Table 1 shows the Linearity dilution scheme.

In the order to measure CD4+ T lymphocyte in the low and high pools, these pools will be stained using Tritest (CD3/CD4/CD45) and Trucount tubes and acquire on the FACSCalibur.

Table: 1 CD4+ T lymphocyte Linearity Dilution Scheme

Dilution	Expected Concentration (cells/µL)	Mixing scheme	Mixing Volume
1	40	Low Concentration Pool (L)	500(µL) L
2	516	0.9L+0.1H	450(µL) L+ 50(µL) H
3	992	0.8L+0.2H	400(µL) L+ 100(µL) H
4	1468	0.7L+0.3H	350(µL) L+ 150(µL) H
5	1944	0.6L+0.4H	300(µL) L+ 200(µL) H
6	2420	0.5L+0.5H	250(µL) L+ 250(µL) H
7	2896	0.4L+0.6H	200(µL) L+ 300(µL) H
8	3372	0.3L+0.7H	150(µL) L+ 350(µL) H
9	3848	0.2L+0.8H	100(µL) L+ 400(µL) H
10	4324	0.1L+0.9H	50(µL) L+ 450(µL) H
11	4800	High Concentration Pool (H)	500(µL) H

5.3.2. Total Lymphocyte Preparation:

Linearity for total lymphocyte will be evaluated over 200-10,000 cells/µL using the low and high concentration cell pools of known concentrations of total lymphocytes; that will be proportionally mixed to achieve eleven cell concentrations, over a range that is 20-30% wider than the anticipated claim range. Refer to the workflow notes for detailed preparation instructions. See below the dilution scheme for Total Lymphocyte Linearity (Table 2).

In the order to measure Total lymphocyte in the low and high pools, these pools will be first stained and lyse using Tritest (CD3/CD4/CD45) and Trucount tubes, and samples will be acquired on the FACSCalibur.

Table: 2 Total Lymphocyte Linearity Dilution Scheme

Dilution	Approximate Concentration Expected (cells/µL)	Mixing scheme	Mixing Volume
1	160	Low Concentration Pool (L)	500(µL) L
2	1344	0.9L+0.1H	450(µL) L+ 50(µL) H
3	2528	0.8L+0.2H	400(µL) L+ 100(µL) H
4	3712	0.7L+0.3H	350(µL) L+ 150(µL) H
5	4896	0.6L+0.4H	300(µL) L+ 200(µL) H
6	6080	0.5L+0.5H	250(µL) L+ 250(µL) H
7	7264	0.4L+0.6H	200(µL) L+ 300(µL) H
8	8448	0.3L+0.7H	150(µL) L+ 350(µL) H
9	9632	0.2L+0.8H	100(µL) L+ 400(µL) H
10	10816	0.1L+0.9H	50(µL) L+ 450(µL) H
11	12000	High Concentration Pool (H)	500(µL) H

5.3.3. Hemoglobin Concentration Preparation:

Assay linearity for Hb concentration will be evaluated over 2-20 Hb in g/dL, using two specimen pools containing known low and high concentrations of Hb. The specimen pools will be proportionally mixed to achieve eleven cell dilutions over a range that is 20-30% wider than the anticipated claim range; refer to the workflow notes for detailed preparation instructions. The Hb Linearity dilution scheme is illustrated on Table 3.

Hb concentration in the low and high pools will be measured on the Sysmex (hematology analyzer).

Table: 3 Hb Linearity Dilution Scheme

Dilution	Expected Concentration (g/dL)	Mixing scheme	Mixing Volume
1	1.5	Low Concentration Pool (L)	500(µL) L
2	3.75	0.9L+0.1H	450(µL) L+ 50(µL) H
3	6	0.8L+0.2H	400(µL) L+ 100(µL) H
4	8.25	0.7L+0.3H	350(µL) L+ 150(µL) H
5	10.5	0.6L+0.4H	300(µL) L+ 200(µL) H
6	12.75	0.5L+0.5H	250(µL) L+ 250(µL) H
7	15	0.4L+0.6H	200(µL) L+ 300(µL) H
8	17.25	0.3L+0.7H	150(µL) L+ 350(µL) H
9	19.5	0.2L+0.8H	100(µL) L+ 400(µL) H
10	21.75	0.1L+0.9H	50(µL) L+ 450(µL) H
11	24	High Concentration Pool (H)	500(µL) H

5.3.4. Sample Testing

Prepare triplicates of each dilution as follows and run them using the IUO BD FACSPresto. Refer to the protocol-specific workflow notes for detailed instructions.

- Label three BD CD4/%CD4/ Hb cartridges for each dilution as instructed in the naming convention section of the workflow notes.
- Prior to pipetting, thoroughly mix each specimen dilution by gentle inversion.
- Using the provided transfer pipettes, transfer 20-25µL of the well-mixed specimen into the well of the appropriately-labeled cartridge.
- Incubate cartridges for a minimum of 18 minutes at room temperature on the incubation tray.

5.3.5. Enrolled Sample Acquisition and Analysis

- Each cartridge will then be run on a single FACSPresto instrument in a randomized order. Refer to the protocol-specific Workflow Notes for the randomization scheme.
- Enter the specimen ID and date information using the touch screen as instructed in the workflow note instructions.
- Once incubation is finished, load the IUO BD cartridge into the instrument and start the run within 2 hrs. from the time of blood transfer onto the IUO BD cartridge.
- The instrument will automatically read and calculate CD4 absolute count, %CD4, and Hb concentration.

Note: No total lymphocyte result will be printed on the instrument prinouts or recorded on the “Summary file Multiple table” CSV file. Total lymphocyte results will be analyzed using “Clinical Data” file.

- Print specimen results. Review the acquisition data for acceptability and check for quality controls messages, if there is quality control message, refer to IFU for troubleshooting.

The following flowchart summarizes steps for specimen preparation and acquisition:

Flowchart 1: Sample Testing

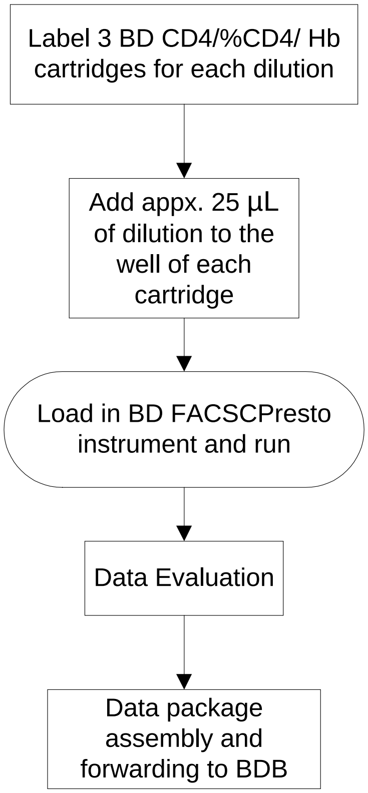

6. DISCONTINUATION OF SPECIMEN TESTING

Post-enrollment, instances may occur that will require the discontinuation of the WB specimen testing such as:

If further visual inspection of an enrolled specimen reveals clotting and/or hemolysis that was not initially apparent, this specimen would be unsuitable for study testing or, if results have been generated, these results would not be included in analysis.

If an enrolled specimen (of sufficient quantity) is broken/spilled, this “quantity not sufficient (QNS)” specimen would require discontinuation of study testing.

7. PRODUCT ERRORS/ DEFECTS/ FAILURES

Errors, defects or unanticipated failures observed for the investigation study product(s) will be documented using the study error forms and communicated immediately to the Study Monitor.

These events will be documented and escalated in accordance with BD internal procedures.

8. DATA COLLECTION AND MANAGEMENT

8.1. Data Collection and Source Documents

Study data will be collected in the form of standardized case report forms (CRFs) and other data collection forms that will be provided to the investigational site. Instrument data will also be collected in the form of instrument records (electronic files or printouts) such as from instrument set-up and sample acquisition results; these are considered source documents.

Investigators will maintain all source documents associated with the protocol, including laboratory results and instrument reports. All hard copy and electronic data will be secured to ensure confidentiality. The source documents will be used for study monitoring and data management review to verify study compliance.

In verifying data integrity, the monitor will ensure that the data in the CRFs are consistent with protocol requirements.

8.2. Submission of Data Package to Sponsor

Investigator is responsible for the collection, review, and timely submission of all required study data to BD for processing, including instrument electronic files, which will be acquired and saved in specified study archives.

All study data that include paper and electronic documentation of the study, will be submitted to BD in a weekly package by courier. The PI or designee will ensure that the data and documentation are accurate, complete, and in compliance with applicable documentation requirements, prior to assembling and submitting data packages to BD.

BD will provide a checklist during site initiation and training that can be referenced during data package compilation and review. This checklist may contain, but not be limited to:

- List of the minimum required package items

Quality assurance procedures should be followed to ensure that complete, accurate and timely data are submitted, that protocol requirements are followed, and that product failures, errors, adverse events and adverse device effects are detected, managed and reported as required, if applicable.

Upon completion of data package review, the site will forward this package to BD in regular shipments at intervals agreed upon by the CRA and PI (typically, once per week).

8.3. Data Evaluation for Inclusion/Exclusion from Analysis

After receipt of data packages from the sites, the BD CRA/study monitor or designee will review the contents to ensure that the data are accurate, complete, and in compliance with all applicable requirements.

A complete dataset is required for each sample in the study. A dataset that does not meet all requirements for evaluability as defined in this section may be excluded from analysis. All excluded samples will be justified in the study final report.

- Instrument setup results must “Pass” for each day’s data collection. Sample results obtained when the instrument setup result does not pass will be excluded from the study.
- Control results must be within range for each day’s data collection. Sample results obtained when the quality control samples fail will be excluded from the study.

- Results obtained with methods not in compliance with all applicable protocol, Instructions For Use, and User's Guide requirements will be excluded.
- Statistical outliers will be investigated and reported. Outlier results with assignable causes may be excluded pending investigation.

In instances where data is found to be missing or incomplete, the CRA will contact the site to resolve data discrepancies using a data clarification form (DCF) for query resolution. Discrepancies may be addressed via calls to the investigational site or during visits by site monitors. Based on the outcome of this review, the CRA may identify and provide a reason for specified data to be deemed not evaluable and possibly excluded from analysis (but not removed from the study database).

8.4. Data Management and Storage

BD Data Management will be responsible for the entry, processing and maintaining of study data.

All original CRFs will be sent to BD as part of the periodic submission package or be retrieved by the site monitor with a copy kept at the site. BD Data Management will process the data and will carry out edit checks and error checks, if needed. The Investigator will be queried on issues concerning data completeness and consistency.

All above-mentioned tasks will be performed according to the Sponsor's relevant internal procedures; ensuring adherence to GCPs.

9. STATISTICAL METHODS

Statistical analyses will be conducted by the BD Biostatistics group.

9.1. Sample Size Determination

The sample size for this linearity study is based on CLSI guidance document EP6-A, which requires 7-11 concentrations over a range that is 20 to 30% wider than the anticipated measuring range.

9.2. Eligibility, Exclusions, Missing Data, Interim Analysis

All specimens meeting the eligibility criteria and tested with the IUO BD FACSPresto System will be analyzed for primary and secondary endpoints. Management of dropouts and missing data will depend on their frequency and the particular outcome measure; any such adjustments will be described completely and documented. All valid data will be included in the analysis; if outliers are determined to exist, the monitor will investigate the identified outliers.

No interim analyses are planned since study duration is 1-2 working weeks.

9.3. General Statistical Considerations

All statistical analyses will be performed using commercially available statistical software. Adequate source document verification and/or audit activities will be utilized to assure the validity of study conclusions. Analysis shall be performed based on methods described in Statistical Analysis Plan, briefly described in section 9.7 Analysis Method of this protocol. All related and resulting reports, documents and data shall be produced and maintained in such a way as to ensure their control and the protection of subject privacy as far as is reasonably practicable. Data files and analytic reports will be archived according to requisite regulatory standards.

9.4. Study Endpoints / Outcome Measures

9.4.1. Primary Endpoint

The primary endpoint of this study is the evaluation of the linearity of the BD FACSPresto™ system (instrument, software, and BD CD4/%CD4/Hb Cartridge Assay) in measuring percentage and absolute counts of CD4+ and total lymphocyte.

9.4.2. Secondary Enpoints

The secondary endpoint of this study is the evaluation of the linearity of the BD FACSPresto™ system (instrument, software, and BD CD4/%CD4/Hb Cartridge Assay) in measuring Hb concentration.

9.5. Acceptance Criteria

The study design described in this protocol is based on CLSI Guidance document, EP6-A.

9.5.1. CD4+ T-Lymphocyte Absolut Count

Across the dynamic range of the assay for AbsCD4 (50 to 4,000 cells per µL), the system shall be linear if 2nd and 3rd order coefficients of the regression lines are not significant. If the coefficients from the higher order polynomial fit (2nd and 3rd) tested are statistically significant, then the difference between the first order linear fit and the higher order linear fit must be within 10% of the first order linear fit for concentration levels that are >200 cells/ µL, or within ±20 cells/µL of the first order linear fit for concentration levels that are ≤200 cells/ µL. The coefficient of determination ((R ^ 2) shall be >95% for linear fit. (Design Input Document v6.0, MICD4-DID-388)

9.5.2. Total Lymphocyte Count

Across the dynamic range of the assay for total lymphocte count (200 to 10,000 cells per µL), the system shall be linear if 2nd and 3rd order coefficients of the regression lines are not significant. If the coefficients from the higher order polynomial fit (2nd and 3rd) tested are statistically significant, then the difference between the first order linear fit and the higher order linear fit must be within 10% of the first order linear fit for concentration levels that are >200 cells/ µL, or within ±20 cells/ µL for concentration levels that are ≤200 cells/ µL. The coefficient of determination ((R ^ 2) shall be >95% for linear fit. (Design Input Document v.6.0, MICD4-DID-467)

9.5.3. Hemoglobin Concentration

Across the dynamic range of the assay for Hb concentration (2 to 20 g/dL), the system shall be linear if 2nd and 3rd order coefficients of the regression lines are not significant. If the coefficients term from higher order polynomial fit (2nd and 3rd order terms) tested are statistically significant, then the difference between the first linear fit and the higher order linear fit must be < 7% different from the linear fit, except for Hb ≤ 7.1 g/dL, at which point it must be within 0.5 g/dL from the first order linear fit at each level for Hemoglobin counts. The coefficient of determination ((R ^ 2) shall be >95% for linear fit. Design Input Document v6.0, MICD4-DID-393)

9.6. Methods to Reduce Bias

To minimize operator error or bias, all operators participating in the study will have documented training in the procedures for staining, instrument set up, sample acquisition and sample analysis.

Bias from specimen preparation will be minimized by using only one trained operator to prepare the specimen dilutions.

Instrument bias will be minimized by acquiring all data on a single device, and ensuring instrument qualification has been performed by a qualified BD Service representative prior to starting the study.

9.7. Analysis Method for the Linearity Procedure

The design and proposed analysis for this evaluation are based on recommendations given in the CLSI guidance document EP6-A[9].

Polynomial regression models (1st order, 2nd order and 3rd order) will be fitted to the results as a function of the expected values or coded levels of each concentration as independent variables, and the specified dependent variable:

- CD4 Absolute Counts
- Total Lymphocyte Count

- Hb concentration

The coefficients for the nonlinear terms (the 2nd order and 3rd order terms) of the higher order models are tested to determine statistical significance. If the higher order coefficient terms are not significant, then the data are linear with no further analysis required. If the higher order coefficients are significant, then the difference between the best higher order fit and the linear fit will be calculated and subjected to the acceptance criteria. If the difference is within the acceptance criteria for each level, then the data are adequately linear.

9.8. Biostatistics Report

A statistical report will be prepared by the biostatistician after data analysis is complete.

10. STUDY MATERIALS AND SUPPLIES

10.1. Investigational Product Labeling

The investigational products will be provided bearing “IUO” label/labeling indicating that they must only be used for investigational purposes (i.e. “For Investigational Use Only” labeling). The BD FACSPresto System (instrument, cartridges, and software) will be provided bearing labels/labeling indicating “IUO”. Additional information regarding system operation and reagent composition will be contained in IUO instructions for use and package inserts that will be provided to the site prior to or during study initiation.

Additional information regarding product composition, handling, etc. will be contained in an IUO package insert.

10.2. Study Products Shipment and Receipt

BD will include appropriate shipping log(s) in all material shipments to the sites: Study Accountability Log(s) for IUO/IVD Instrument, Reagents, Software; and/or Miscellaneous Materials Shipment Log(s).

All IUO or IVD supplies for training and for the study will be verified upon delivery by site personnel and entered on the center's Study Accountability Logs. After receipt has been documented on Study Accountability logs, the log originals are retained by the site to allow for documentation of usage and final disposition of all investigational materials (i.e., used, unused and destroyed at the site, or unused and returned to BD).

10.3. Study Product Accountability

The Sponsor will provide the Investigator with investigational product upon receipt of all necessary study documentation.

During the investigation, the Investigator is responsible for ensuring that:

- only specimens that qualify for study enrollment are run on the IUO system;
- at the testing laboratory, all investigational products and devices are maintained under controlled access storage, and the use of investigational products are strictly controlled;
- any device or components that are removed from their packaging and not used are segregated from general use for the duration of the study.

The Investigator is responsible for maintaining accurate accountability records for the use of investigational materials on the Study Accountability Logs. These records will be reviewed at all monitoring visits to ensure compliance, and will document:

- Receipt dates and quantity,
- Dates and quantity used, and
- Amount used, discarded or returned.

Study Accountability Logs will be verified by the site monitor against the Shipping Logs.

10.4. Required Study Materials and Supplies

10.4.1. Instrumentation

BD will provide IUO products:

- BD FACSCalibur
- BD FACStation workstation
- BD Multiset Software
- BD FACStation Software (includes FACSComp and Worklist Manager)
- IUO BD FACSPresto System instrument and accessories
- Sysmex (KX-21)

10.4.2. Reagents, and Controls

- BD Calibrite beads
- BD Tritest CD3/CD4/CD45 reagent
- BD Multi-Check normal and CD4 low controls for FACS Calibur
- BD Trucount tubes
- BD FACS Lysing Solution
- BD FACSFlow for the FACSCalibur
- BD CD4/%CD4/Hb cartridges or IUO BD cartridges
- Eurotrol 301 (Level 1, 2, 3)
- Streck CD-Chex CD4 Normal and Low
- Sysmex reagents (SYSPK-30L; SYSSWH-200A)
- Sysmex L, N and H controls

10.4.3. General Product Use and Study-Specific Documents

- IUO BD FACSPresto System IFU
- IUO FACSPresto Safety and Limitations Guide
- IUO BD cartridge IFU

10.4.4. Ancillary Items

BD will provide the following:

- Transfer pipettes
- CDs or USB memory sticks for archival of the BD FACSPresto result files
- BD FACSPresto printer rolls

Site should have access to the following:

- Vortex mixer
- Lab timer

10.5. Disposition

At the end of the study or upon request by the site monitor, any remaining investigational devices, used or unused, will be handled as follows:

10.6. Reagents

The study monitor will instruct whether all used IUO BD cartridges, as well as those opened but not used, will be returned or defaced and discarded at the site. No experimental devices or supplies will be used for any other purpose except for testing the specimens enrolled in this clinical study.

Unused reagents: At the end of the study or as instructed by the site monitor, the Investigator or his/her designate will return or deface and discard all remaining unused supplies to BD.

11. MONITORING

11.1. Study Monitors

The study monitor's responsibilities are to ensure that:

- the study protocol is followed,
- timely and accurate data are submitted,
- inconsistent, incomplete or inaccurate data is corrected,
- site facilities, staff and performance continue to be sufficient to ensure the validity of the scientific data, adherence to study requirements, and the protection of the health, safety and welfare of the study subjects.
- prompt reporting as required by the investigational plan and FDA regulations is achieved, especially regarding Unexpected Adverse Device Effects (21CFR part 812.50)

Study monitoring will comply with the requirements of GCP Guidelines and Section 9 of ISO 14155:2011, Clinical investigations of medical devices. Study monitoring will be performed by qualified personnel, with appropriate education and / or experience to perform all specified and necessary monitoring tasks.

11.2. Monitoring Procedures

11.2.1. Case Report Forms (CRFs) Management

The site monitor will ensure that CRFs are completed for each included specimen in a timely fashion. Electronic files and instrument printouts will be monitored on-site and in-house.

Corrections and modifications made to data already written or completed on the CRF page should be legible, initialed and dated by approved personnel. The reasons for significant changes must be provided. Correction fluid or covering label must not be used.

11.2.2. Monitoring Procedure and Documentation

Monitoring visits will be conducted in accordance with Clinical Operations Standard Operating Procedures (SOPs), Desk Procedures (DPs), the study protocol, monitoring plan and applicable regulatory requirements. Monitoring visits to the study site will be scheduled periodically during the study, to ensure that Investigators and their staff understand and accept their defined responsibilities and that all aspects of the current approved protocol/amendment(s) are followed. Data collection forms, electronic files and study data will be reviewed for cross referencing for accuracy.

The Investigator/institution guarantees access to study documents and source documents related to the study by designated BD personnel and appropriate regulatory authorities.
It is important that the Investigator and their relevant personnel are available during the monitoring visits and possible audits and that sufficient time is devoted to the process. The Site monitor will complete a report documenting completion of the each site visit.

All site visits will be recorded in a monitoring log maintained at each investigational site documenting the monitor and the date of the monitoring visit.

- <u>Site Initiation Visit:</u> Prior to a clinical investigation, the BD monitor will ensure that the Investigator and the investigational site are prepared for the study. All the appropriate training materials and the user documentation for this clinical investigation will be provided to study personnel during the site initiation visit. Documentation of all necessary approvals, agreements, and training on the study and experimental treatment will be gathered during the visit.
- <u>Interim Site Visit:</u> A BD monitor will schedule and conduct Interim Monitoring Visits to monitor the clinical investigation throughout its active phase to ensure compliance with this protocol, IUO device accountability and data accuracy. The first Monitoring Visit during the active phase of the study will be scheduled according to the study monitoring plan.
- <u>Site Close-out Visit:</u> The BD monitor will assure that all site closeout activities are addressed when the study has been completed or terminated at the clinical site as indicated in the study monitoring plan.

- Compliance visits: In the event that a study site requires additional assistance to meet study protocol or regulatory requirements, monitoring visits will be conducted at more frequent intervals and / or with additional personnel to assure compliance.
- Other visits: the study site may be inspected by appropriate regulatory authorities this includes visits by Ministry of Health representative(s).

11.3. Audits/Inspections

The site may be selected for an audit by the sponsor (e.g., BD Medical and Quality Assurance groups) or by a regulatory authority. It is responsibility of the PI to facilitate the auditing process by granting all required access to the study site, study documentation, source data and study personnel. When the PI is notified of an upcoming audit by a regulatory authority, he/she must notify BD as soon as possible.

12. SAFETY REPORTING

Injuries or safety issues related to the use of an investigational study product(s) must be reported immediately to the Sponsor. This report should provide details of any unusual event concerning an operator/user of the device/system and the steps taken to provide treatment or abatement of any injuries or correction of the event. Upon receipt, the Sponsor will evaluate each report to determine if the event could affect the integrity of the study data or the operation of the system, or if it meets criteria of an “Adverse Event” as determined by BD Medical and Regulatory Affairs. All Serious Adverse Events (SAE) must be reported by the Principal Investigator to the EC/IRB (and, if appropriate, any regulatory agency) within the specified time periods described in local/country regulations.

All adverse events occurred during the conduct of the study will be reported to Sponsor using the appropriate form.

13. STUDY SUSPENSION OR TERMINATION

Either the BDB Clinical Operations or BDB program team may terminate this study for a reasonable cause. Reasons for suspension or early termination may include the following:

- Previously unanticipated and insurmountable administrative or scientific difficulties or obstacles
- Change in BDB project priorities

14. STUDY ETHICS/ GOOD PRACTICES

The procedures set forth in this study protocol are designed to ensure that BD and clinical investigators abide by the ICH GCP Guidelines, regulations of the FDA, and the Declaration of Helsinki in the conduct, evaluation and documentation of the study.

14.1. Ethics Review and Approval

The linearity study testing will be conducted using normal human blood that has been received as a de-linked and coded specimen from the BDB Blood Donor Program. This program is responsible for ensuring that the procurement process for these coded specimens is in accordance with their applicable institutional policies and procedures and under approval/oversight by the their institution’s IRB regarding both any additional risk or harm to the subjects or risk of disclosure of confidential personal health information.

14.2. Informed Consent

For this linearity procedure, additional Informed Consent will not be required because the normal blood specimens will be procured from the BDB Blood Donor Program. This blood donor program conducts their prospective procurement of specimens for BD internal clinical studies using an approved protocol

and informed consent form under IRB oversight and, the specimens will be provided delinked and unidentified from the donors protected health information, as coded specimens.

14.3. Risks and Benefits

This study presents no medical risks or harm to subjects other than a possible mild discomfort or bruising as can be expected with normal phlebotomy procedures that will be managed under the BD Blood Donor Program procedures. There is no risk of disclosure of confidential protected health information because the peripheral blood samples will be received delinked and coded specimens. There are no direct benefits to the subject for participation in this study and no investigational testing results will be reported. The outcome of the study will be the evaluation of the system performance that is a tool for monitoring the immune status of individuals, especially those with immunodeficiency diseases and/or disorders.

14.4. Donor Identification and Confidentiality

All specimens will be provided the BD MEDLab as de-linked and coded specimens.

14.5. Donor Remuneration

This is not applicable for this study.

15. INVESTIGATOR

15.1. Clinical Investigators

The Clinical Investigator and staff will meet GCP requirements. The Investigator, approved sub-Investigators, study operators and all supporting staff at the Investigator's site must be appropriately qualified by education and / or experience to perform their tasks.

15.2. Submission and obtaining Ethics Approval

This is not applicable for this study (refer to section 14.1).

15.3. Source Documents

Case Report Forms and instrument data will be source document for this study.The Investigator agrees that the Study monitors, Sponsor's employees, contractors or designees, as well as any regulatory bodies as required, will have the right to audit and review source documents and study records relating to this clinical trial. The Investigator and staff will assist with the production, review, interpretation and / or correction of such records as required.

15.4. Device Accountability

Investigator or designee will maintain an accurate Accountability Log(s) and records of the investigational product received, used and unused. Study supplies must be used only for specimen testing described in this protocol.

Principal investigator must supervise the use of investigational device only for the purpose of this study and under the Investigator's supervision. An Investigator shall not supply an investigational device to any person not authorized to receive or use it.

15.5. Data Recording and Record Retention

The Investigator(s) will ensure that the Case Report Forms, instrument records/ printouts, and other study documents are maintained in a secure and confidential manner. The Investigator(s) will ensure that the medical records, Case Report Forms and other study documents are made available for review by the study monitor and any government regulatory bodies as required.

The Sponsor or designee will ensure accuracy and completion of CRFs and hard copies are maintained in the study files.

All essential study documentation (e.g., CRFs, other data collection forms, instrument printouts, and electronic files) must be retained by the PI for a minimum of two years after notification from BD that the study is completed, terminated, or that the investigational product has been successfully released for commercialization, whichever is the longer time period. In the instance where the study PI withdraws as the responsible party, written notification containing the contact information of the PI designee must be forwarded to BD.

As the study sponsor, BD will retain all essential study documentation indefinitely (either in-house or off-site).

15.6. Investigator Reports

This section is not applicable (refer to section 14.1).

15.7. Withdrawal of EC/IRB Approval

This section is not applicable (refer to section 14.1).

15.8. Deviation from the Study Protocol

The Investigator shall notify Sponsor of any changes in, or deviations. BDB will determine the effect of the protocol deviation on the scientific soundness of the clinical data and document according to internal procedures.

15.9. Use of Device without Informed Consent (applicable if Informed Consent is required)

This section is not applicable (refer to section 14.2).

15.10. Final Report

Not applicable for this study.

15.11. Other Reports

Not applicable for this study.

15.12. Responsibility for Investigator Reports

The Principal Investigator may delegate a qualified associate(s) to complete one or more of the above functions. However, the Principal Investigator retains the overall responsibility for the proper conduct of the study, compliance with this study plan, and the collection of all required data, as well as subject safety where applicable.

15.13. Financial Disclosure

A clinical Investigator shall disclose to the Sponsor sufficient accurate financial information to allow the applicant to submit complete and accurate certification or disclosure statements required by the regulations. The Investigator shall promptly update this information if any relevant changes occur during the course of the investigation and for 1 year following completion of the study.

16. SPONSOR

16.1. Sponsor Staff Qualifications

The BD Clinical Operations employees and contractors, including study monitors, will be appropriately qualified by education and / or experience to perform their tasks. Resources, systems, standard

operating procedures and training adequate to oversee the scientific, ethical and regulatory aspects of the clinical study will be maintained throughout the period of the clinical investigation.

16.2. Beginning the Study

Before the start of study and testing, BD must have the following documents from the clinical site:

- CVs signed and dated from the Principal investigator and site staff participating in the trial (Sub-Investigator, Technologists, etc.)
- Approved Investigator's Agreement
- Principal investigator and Sub-Investigator financial disclosure

16.3. Selecting Investigators

Sponsor shall select Investigators qualified by training and experience to investigate the Precision Evaluation of the IUO BD FACSPresto System and IUO BD FACSPresto cartridge.

16.4. Ensuring Proper Monitoring

Sponsor shall select monitor(s) qualified by training and experience to monitor the progress of the investigation.

16.5. Ensuring Proper Notification

Not applicable for this study.

16.6. Control of Device

Sponsor shall ship investigational devices only to qualified Investigators participating in the study.

16.7. Obtaining Agreements

Sponsor shall obtain a signed and dated Investigator Agreement; the Investigator's CV; a statement of the Investigator's relevant experience (if applicable), including dates, location and type of experience; explanation of circumstances leading to termination of studies previously undertaken by the Investigator (if applicable); a statement that the Investigator is committed to conduct the investigation in accordance with the agreement, the investigational plan, other applicable FDA regulations, and other conditions imposed by the Ethics Committee/FDA.

16.8. Securing Compliance

Sponsor must ensure that the Investigator continues to comply with the signed agreement, the investigational plan, other applicable FDA regulations and other conditions imposed by the Ethics Committee/FDA, or discontinue shipments of device to the Investigator, terminate the Investigator's participation in the investigation and require such an Investigator to return device, unless this action would jeopardize the rights, safety or welfare of a subject.

16.9. Resumption of Suspended or Terminated Studies

Either the BDB Clinical Operations or BDB program team may terminate this study for a reasonable cause. Reasons for suspension or early termination may include the following:

- Previously unanticipated and insurmountable administrative or scientific difficulties or obstacles
- Change in BDB project priorities

16.10. Reporting

A Final Study Report meeting the requirements of ISO 14155:2011, Annex C and relevant country regulations will be prepared by the Sponsor.

16.11. Records

Sponsor or delegate shall maintain accurate, complete, and current study documentation.

17. PUBLICATION

Publication of the study results in the medical literature will be attempted, under the authorship of the Investigators; however; BD reserves the right to comment on publications prior to submission in order to protect intellectual property rights and confidential information.

18. REFRENCES

1. E6, International Conference on Harmonization: Good Clinical Practice: Consolidated (Published in the Federal Register May 9, 1997)
2. Estaquier J, Idziorek T, de Bels F, Barré-Sinoussi F, Hurtrel B, Aubertin AM, Venet A, Mehtali M, Muchmore E, Michel P, Mouton Y, Girard M, Ameisen JC. Programmed cell death and AIDS: significance of T-cell apoptosis in pathogenic and nonpathogenic primate lentiviral infections. Proc Natl Acad Sci U S A. 1994 Sep 27;91(20):9431-5.
3. Antiretroviral Therapy of HIV Infection in Adults and Adolescents: Recommendations for a Public Health Approach. World Health Organization, 2010 revision.
4. Volberding PA, Lagakos SW, Koch MA, Pettinelli C, Myers MW, Booth DK, Balfour HH Jr, Reichman RC, Bartlett JA, Hirsch MS, Zidovudine in asymptomatic human immunodeficiency virus infection. A controlled trial in persons with fewer than 500 CD4-positive cells per cubic millimeter. The AIDS Clinical Trials Group of the National Institute of Allergy and Infectious Diseases. N Engl J Med. 1990 Apr 5;322 (14):941-9.
5. Cohen MS, Chen YQ, McCauley M, Gamble T, Hosseinipour MC, Kumarasamy N, et al. Prevention of HIV-1 infection with early antiretroviral therapy. N Engl J Med 2011; 365(6):493-505.
6. Thomas TK, Masaba R, Borkowf CB, Ndivo R, Zeh C, Misore A, et al. Triple-antiretroviral prophylaxis to prevent mother-to-child HIV transmission through breastfeeding--the Kisumu Breastfeeding Study, Kenya: a clinical trial. PLoS Med 2011; 8(3):e1001015.
7. Henry DH, Beall GN, Benson CA, Carey J, Cone LA, Eron LJ, Fiala M, Fischl MA, Gabin SJ, Gottlieb MS, et al. Recombinant human erythropoietin in the treatment of anemia associated with human immunodeficiency virus (HIV) infection and zidovudine therapy. Overview of four clinical trials. Ann Intern Med. 1992 Nov 1;117(9):739-48.
8. Firnhaber C, Smeaton L, Saukila N, Flanigan T, Gangakhedkar R, Kumwenda J, La Rosa A, Kumarasamy N, De Gruttola V, Hakim JG, Campbell TB. Comparisons of anemia, thrombocytopenia, and neutropenia at initiation of HIV antiretroviral therapy in Africa, Asia, and the Americas. Int J Infect Dis. 2010 Dec; 14(12):e1088-92. Epub 2010 Oct 18.
9. Clinical Laboratory Standards Institute. Evaluation of the Linearity of Quantitative Measurement Procedures: A Statistical Approach; Approved Guideline. CLSI document EP6-A Vol. 23, No 16. April, 2003.

19. CHANGE HISTORY

Version	Date	Name	Change Description
1.0	11NOV2013	Maryam Saleminik	Initial Version
V1.1	17DEC2013	Maryam Saleminik	Update to reflect DID changes
