# Supplementary material for: Clinical Evaluation of the BD FACSPresto™ Near-Patient CD4 Counter in Kenya
Source: PLoS One. 2016 Aug 2;11(8):e0157939. doi: 10.1371/journal.pone.0157939 (PMC4970792; doi:10.1371/journal.pone.0157939)
Supplement: S1 Table — (DOCX) [file pone.0157939.s010.docx]

| **S1 Table: Clinical Evaluation Inclusion-Exclusion Criteria final** | | | |
| --- | --- | --- | --- |
| **Accuracy and Stability** | | | |
|  | **Inclusion Criteria** | | **Exclusion Criteria** |
| **Subject** | - Has been infected with HIV and willing to provide written informed consent to draw venous and capillary blood. - If a minor, parent(s) or guardian(s) are willing to provide informed consent to draw blood from the child. - Agrees to grant access to her/his CD4 testing medical records for pre-screening. - Agrees to disclose age and gender - Agrees to disclose co-morbid conditions information: Malaria, Tuberculosis, Anemia, Sickle Cell Anemia, Infections, Thalassemia and other current medical conditions. - Agrees to disclose current medications | - Unwillingness to provide written informed consent. - Unwillingness to disclose medical information regarding previous CD4 testing results. - Unwillingness to disclose medical information regarding co-morbid conditions and current medications. - Enrolled specimens may be subsequently excluded from the study if found to be unsuitable for testing; for example, clotting or hemolysis identified by visual inspection. | |
| **Specimen** | - Venous blood collected in a blood collection tube with EDTA anticoagulant and stored at room temperature (20-25°C) and according to the collection tube manufacturer’s guidelines until enrollment. - Venous blood drawn within an adequate time to perform post-enrollment staining within 24 hours. - Venous blood of acceptable quality for flow cytometry testing (e.g., no hemolysis or clots and acceptable pre-analytical handling). - Venous blood of sufficient volume: >1mL for Sample Preparation. - Capillary blood applied onto the PEO/IUO BD CD4/%CD4/Hb cartridge. | - Enrolled specimens may be subsequently excluded from the study if found to be unsuitable for testing; for example, clotting or hemolysis identified by visual inspection. | |
| **Reference Intervals** | | | |
| **Subject** | - Hematological normal male and female subjects of 13 to 65 years of age willing to provide written informed consent - If minor, parent(s) are willing to provide informed consent to draw blood from the minor. - Agrees to disclose age and gender - Agrees to complete Donor’s Questionnaire | - Unwillingness to provide written informed consent. - Diagnosis of hypereosinophylia - Experiencing acute infections (viral or bacterial) - Diagnosis of parasite infestations - Diagnosis of chronic infectious, for example: HIV, TB or other - Diagnosis of hematopoietic disorder, for example: leukemia or myeloproliferative disorders - Anemia - Chronic administration of prescribed prednisone or other corticosteroid medication | |
| **Specimen** | - Venous blood collected in a blood collection tube with EDTA anticoagulant and stored at room temperature (20-25°C) and according to the collection tube manufacturer’s guidelines until enrollment - Venous blood drawn within an adequate time to perform post-enrollment staining within 24 hours - Of acceptable quality for flow cytometry testing (e.g., no hemolysis or clots and acceptable pre-analytical handling) - Of sufficient volume: >1,000μL for Sample Preparation - Capillary blood - Capillary blood meets requirements for specimen donation as required. | - Enrolled specimens may be subsequently excluded from the study if found to be unsuitable for testing; for example, if visual inspection prior to acquisition shows clotting or hemolysis. - If an enrolled specimen (with adequate time post-draw to enroll for study testing) cannot be tested within 24 hours due to unanticipated difficulties, this specimen would be excluded from the study testing. | |
